# Supplementary material for: Thylakoid protein FPB1 synergistically cooperates with PAM68 to promote CP47 biogenesis and Photosystem II assembly
Source: Nat Commun. 2024 Apr 10;15:3122. doi: 10.1038/s41467-024-46863-y (PMC11006888; doi:10.1038/s41467-024-46863-y)
Supplement: Supplementary file 1 — Supplementary information [file 41467_2024_46863_MOESM1_ESM.pdf]

Supplementary Material for

Thylakoid Protein FPB1 Synergistically Cooperates with PAM68 to Promote CP47 Biogenesis and Photosystem II Assembly

Lin Zhang, Junxiang Ruan, Fudan Gao, Qiang Xin, Liping Che, Lujuan Cai, Zekun Liu, Mengmeng Kong, Jean-David Rochaix, Hualing Mi, Lianwei Peng*

Correspondence to Lianwei Peng (E-mail: penglianwei@shnu.edu.cn).

This PDF file includes:

Supplementary Figures 1 to 11

Supplementary Table 1

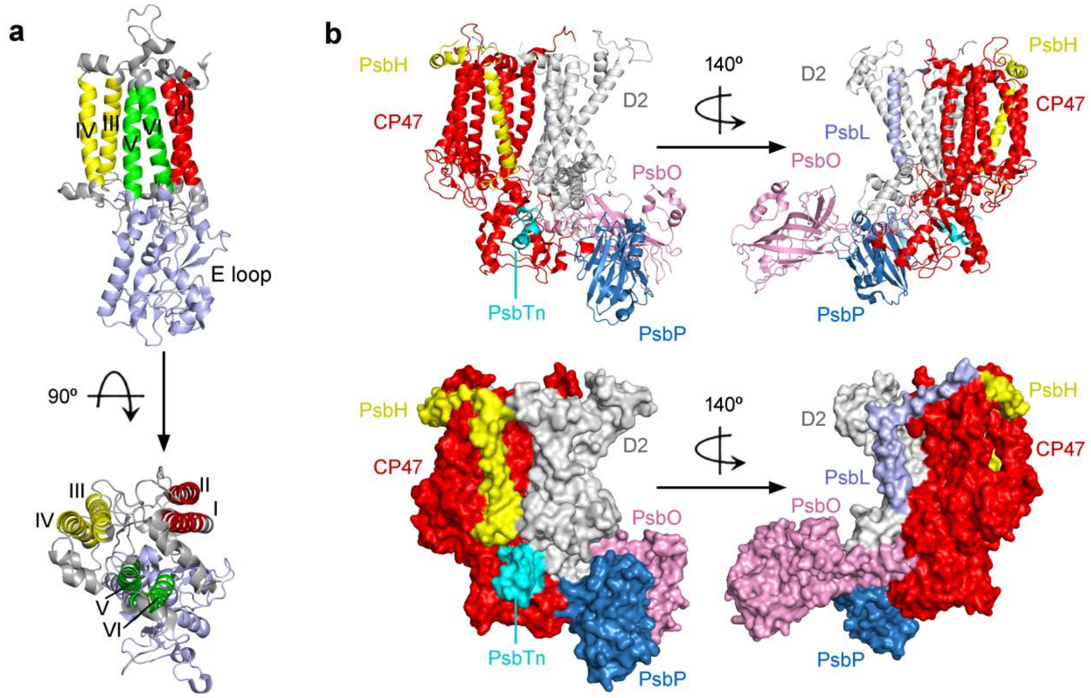

Supplementary Fig. 1 Overall structure of CP47 and its associated PSII subunits in spinach. a Structure of CP47 viewed along the membrane plane (upper) and along the membrane normal from the stromal side (bottom). The TMDs of I/II, III/IV, V/VI, and the E loop are coloured as indicated. **b** Structure of CP47 and its associated subunits viewed along the membrane plane. The proteins are shown with ribbon models (upper) and surface models (bottom) and are coloured as indicated. Protein structures are visualized using PyMOL based on PDB ID 3JCU[1].

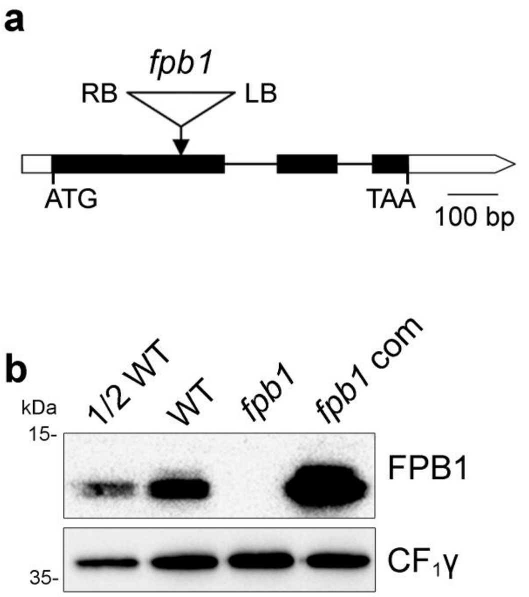

Supplementary Fig. 2 Genetic characterization of the *fpb1* mutant. a Structure of the *FPB1* gene and T-DNA insertion site in the *fpb1* mutant. White boxes, black boxes, and lines represent UTRs, exons, and introns, respectively. T-DNA insertion occurred in the first exon of *fpb1* (SALK_048033). **b** Immunoblot of FPB1 protein. Equal amount of total protein was separated by SDS-urea-PAGE and transferred onto nitrocellulose membranes for immunobloting using antibodies against FPB1 and $CF_1\gamma$ (loading control). Blots are representative of two independent experiments. Source data are provided as a Source Data file.

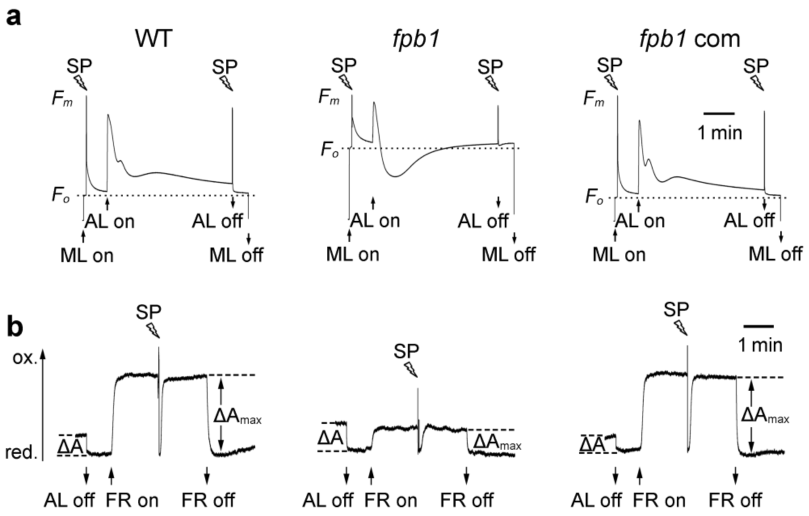

Supplementary Fig. 3 Photosynthetic characterization of *fpb1*. a Chlorophyll *a* fluorescence transients. After dark adaption for 20 min, leaves from four-week-old plants were illuminated with measuring light (ML) to determine the minimum chlorophyll *a* fluorescence (F_o). Subsequently the maximum chlorophyll *a* fluorescence (Fm) was measured by firing a saturating pulse (SP). Then the leaves were exposed to the actinic light (AL, 64 μmol photons m^{-2} s^{-1}) for 4 min. **b** P700 absorbance kinetics. After exposure to AL (209 μmol photons m^{-2} s^{-1}) for 3 min, leaves were illuminated with FR (far-red) light for 4 min to induce maximal oxidation of P700 (ΔAmax). During illumination, re-reduction of $P700^+$ was induced with a saturating pulse (SP). Source data are provided as a Source Data file.

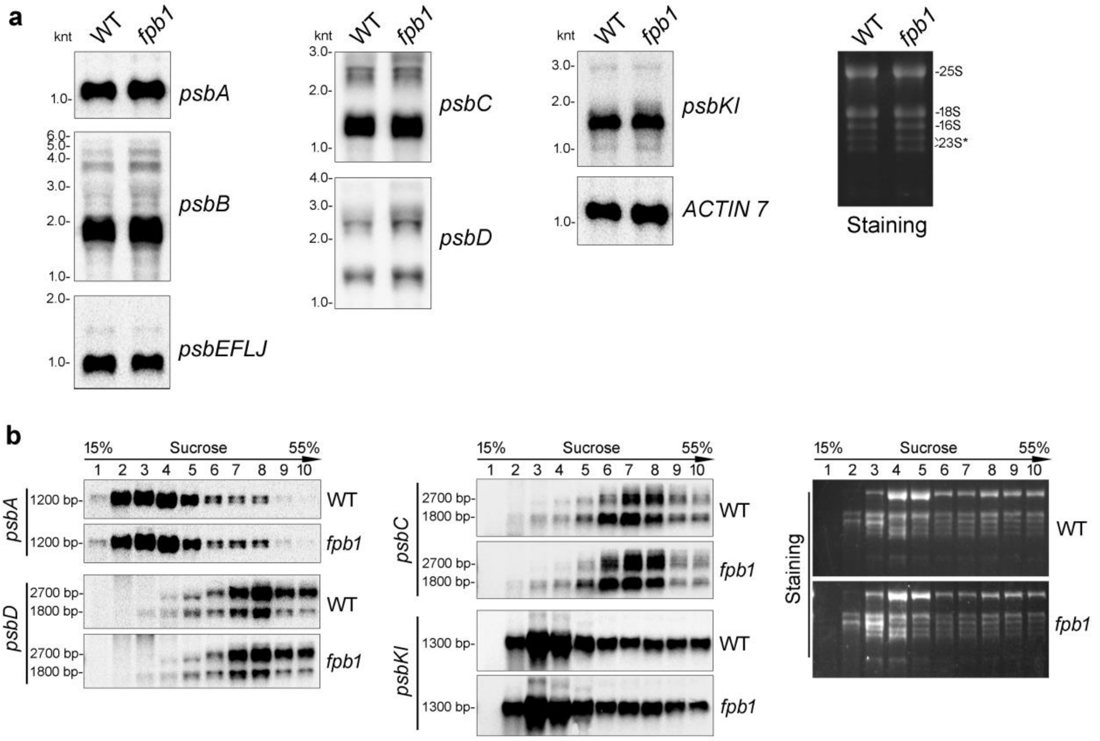

Supplementary Fig. 4 Abundance of the *psb* transcripts and their association with polysomes. a RNA gel blot analyses. Total RNA isolated from WT and *fpb1* leaves of four-week-old plants was probed with DIG-labelled specific probes as indicated. An *ACTIN 7* probe and RNA staining were used as loading controls. The sizes for the RNA lad der are indicated. **b** Polysome association studies of WT and *fpb1* plants. Whole leaf extracts from three-week-old plants were separated by centrifugation in 15-55% sucrose density gradients. The gradients were then divided into 10 fractions and RNA was isolated for gel-blot analyses with DIG-labelled DNA probes corresponding to the *psbA*, *psbD*, *psbC*, and *psbKI* mRNAs. The rRNAs were stained and used as loading controls. Approximate sizes of the main bands are shown and the sizes of the corresponding bands can found in (**a**). Blots are representative of at least two independent experiments. Source data are provided as a Source Data file.

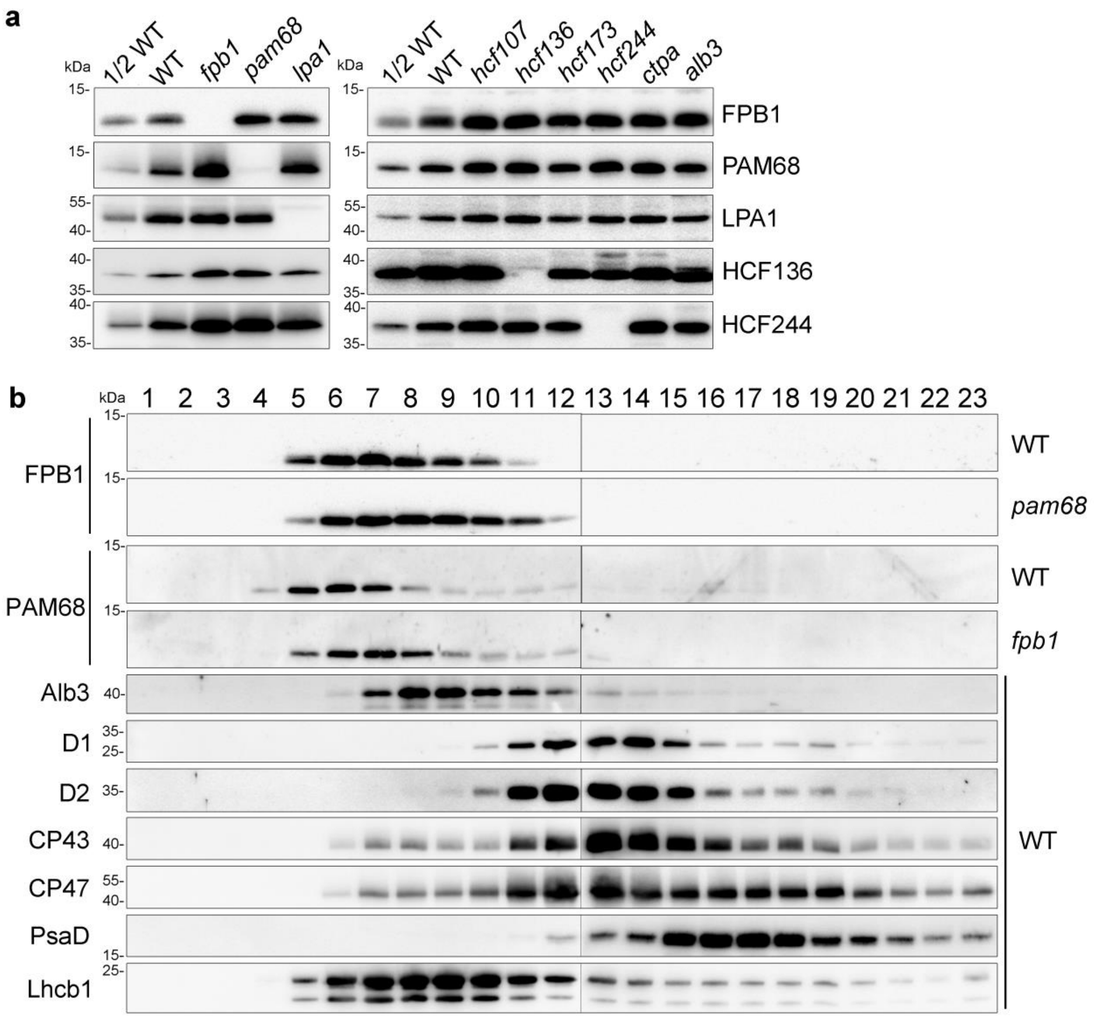

Supplementary Fig. 5 Accumulation of FPB1 and PAM68 in PSII mutants and their complex formation. a Immunoblot analysis of FPB1, PAM68, LPA1, HCF136, and HCF244 in various mutants. Thylakoid membranes isolated from four-week-old *fpb1*, *pam68*, and wild-type plants grown on soil as well as from the other mutants grown on MS medium containing 3% sucrose were subjected to SDS-urea-PAGE with equal protein loading. **b** Sucrose density gradient analyses of FPB1 and PAM68. Freshly isolated thylakoids from four-week-old plants were solubilized with 1% β-DM and protein complexes were separated on a linear sucrose gradient (0.1 M to 1.0 M). Twenty-three fractions of equal volume were collected from top to bottom of the gradients and the proteins were subjected to SDS-PAGE (for Alb3) or SDS-urea-PAGE (for others) and then probed with antibodies as indicated. Blots are representative of at least two independent experiments. Source data are provided as a Source Data file.

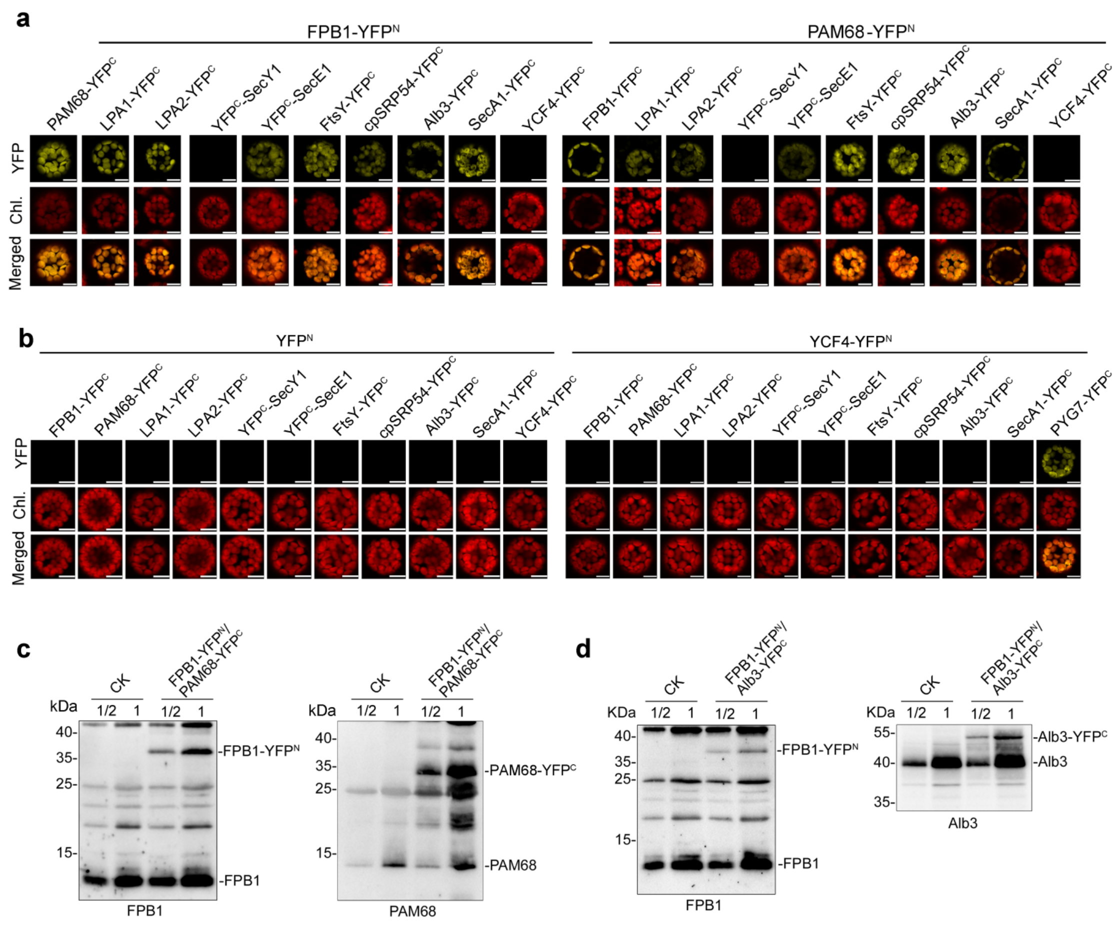

Supplementary Fig. 6 BiFC assay for the interaction of FPB1 and PAM68 with chloroplast proteins in Arabidopsis protoplasts. a Full-length FPB1 and PAM68 were fused with the N-terminal part of YFP (YFP^N) at their C-terminus. Other proteins indicated at the top were fused with the C-terminal part of YFP (YFP^C) at their N- or C-terminus to ensure that the fused YFP^C is present on the stromal side of thylakoids (See Methods). The fusions were transiently co-expressed in Arabidopsis mesophyll protoplasts and the signal was observed using confocal microscopy. Bars =10 µm. **b** Chloroplast targeting sequence of RbcS and the PSI assembly factor YCF4 were fused with the N-terminal part of YFP (YFP^N) at their C-terminus. The fusions and the proteins fused with the C-terminal part of YFP (YFP^C) were transiently co-expressed in protoplasts. Bars =10 µm. **c, d** Immunoblot analysis of the fusion proteins in protoplasts expressing FPB1-YFP^N/PAM68-YFP^C (**c**) and FPB1-YFP^N/Alb3-YFP^C (**d**). Thylakoids were isolated from protoplasts and the proteins were separated by SDS-urea-PAGE (FPB1 and PAM68) or SDS-PAGE (Alb3) and immunoblotted with antibodies. Protoplasts without plasmid transformations were used as a negative control (CK). Protein dilutions were loaded as reference in the blots. Data are representative of two independent experiments. Source data are provided as a Source Data file.

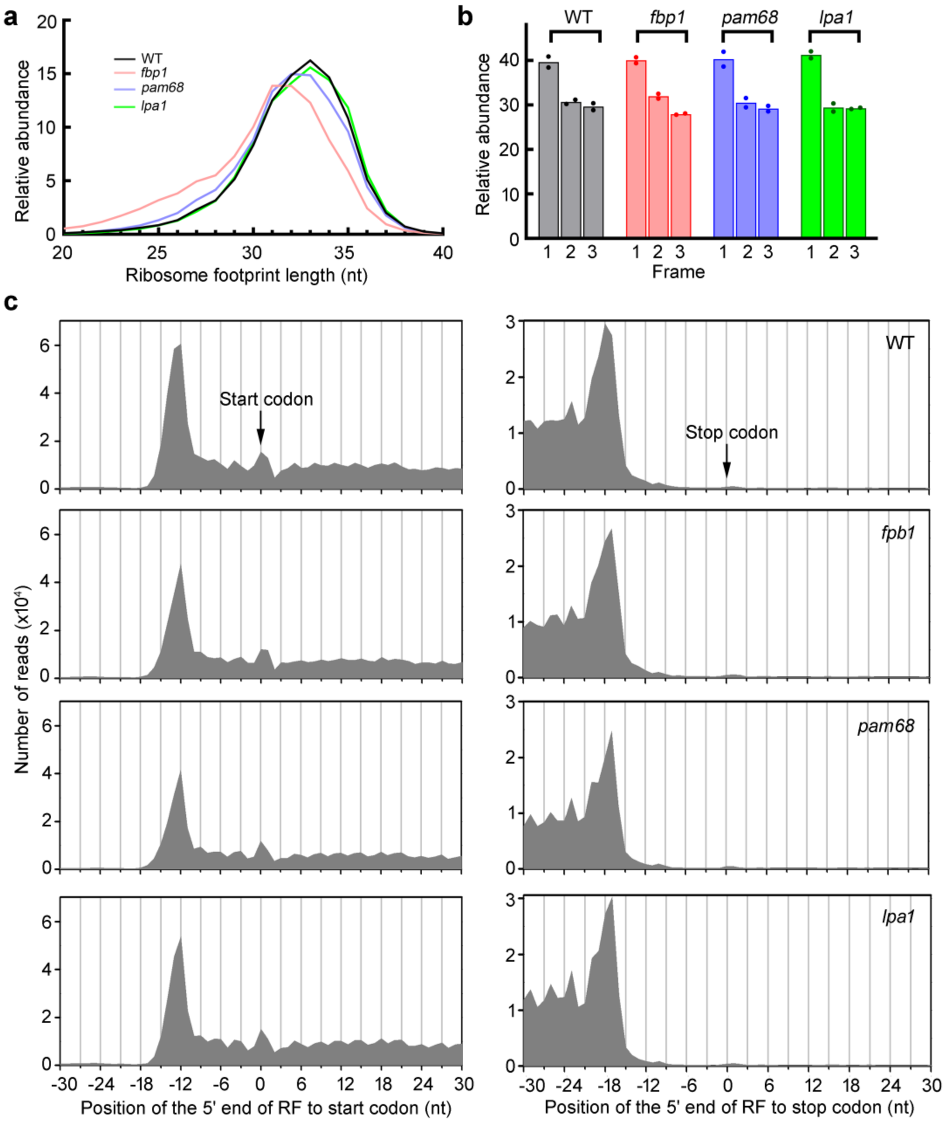

Supplementary Fig. 7 Assessments of Ribo-seq data. a Read length distribution of ribosome footprints. Values are the means from two independent replicate samples in the first experiment. **b** Three-nucleotide (nt) periodicity of the Ribo-Seq data. Fractions of 31-33 nt at the first (1), second (2), and third (3) nucleotide positions of the start codon are shown. Values are the means with single dots for two independent replicate samples in the first experiment. **c** Meta-analysis of ribosome footprints mapping near the start and stop codon. Values show the number of total footprints with 5' end at each position from the first replicate sample. All genes (22895 detected genes) with unique mapped reads of ribosome footprints were analysed. Source data are provided as a Source Data file.

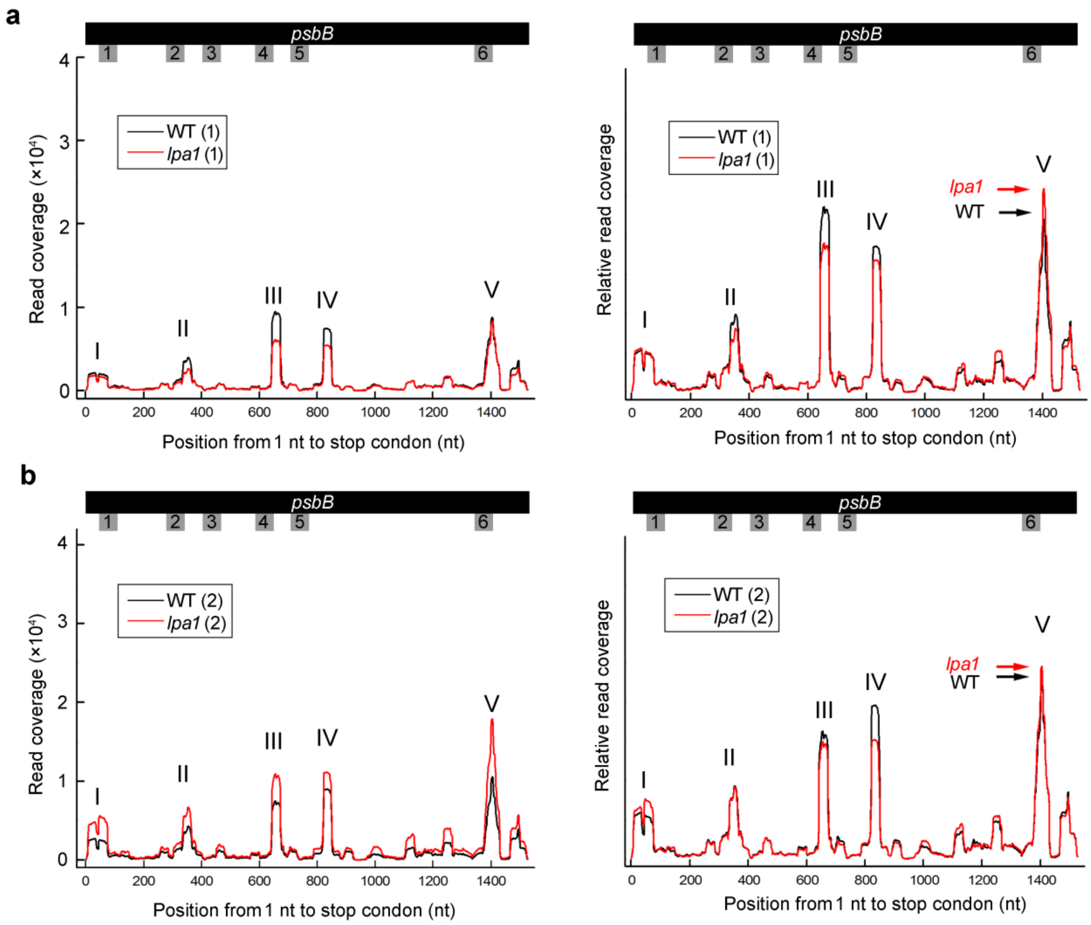

Supplementary Fig. 8 Distribution of ribosome footprints along the *psbB* ORF in WT and *lpa1* mutant plants. The Y-axis shows the total reads at each position (left) or normalized based on the total reads (right). Transmembrane domain (TMD)-encoding positions on the *psbB* mRNA are indicated from 1 to 6. The data from the first (**a**) and second (**b**) replicates of WT are the same as in Figs. 6c and d, since these genotypes were analysed in a same experiment. Source data are provided as a Source Data file.

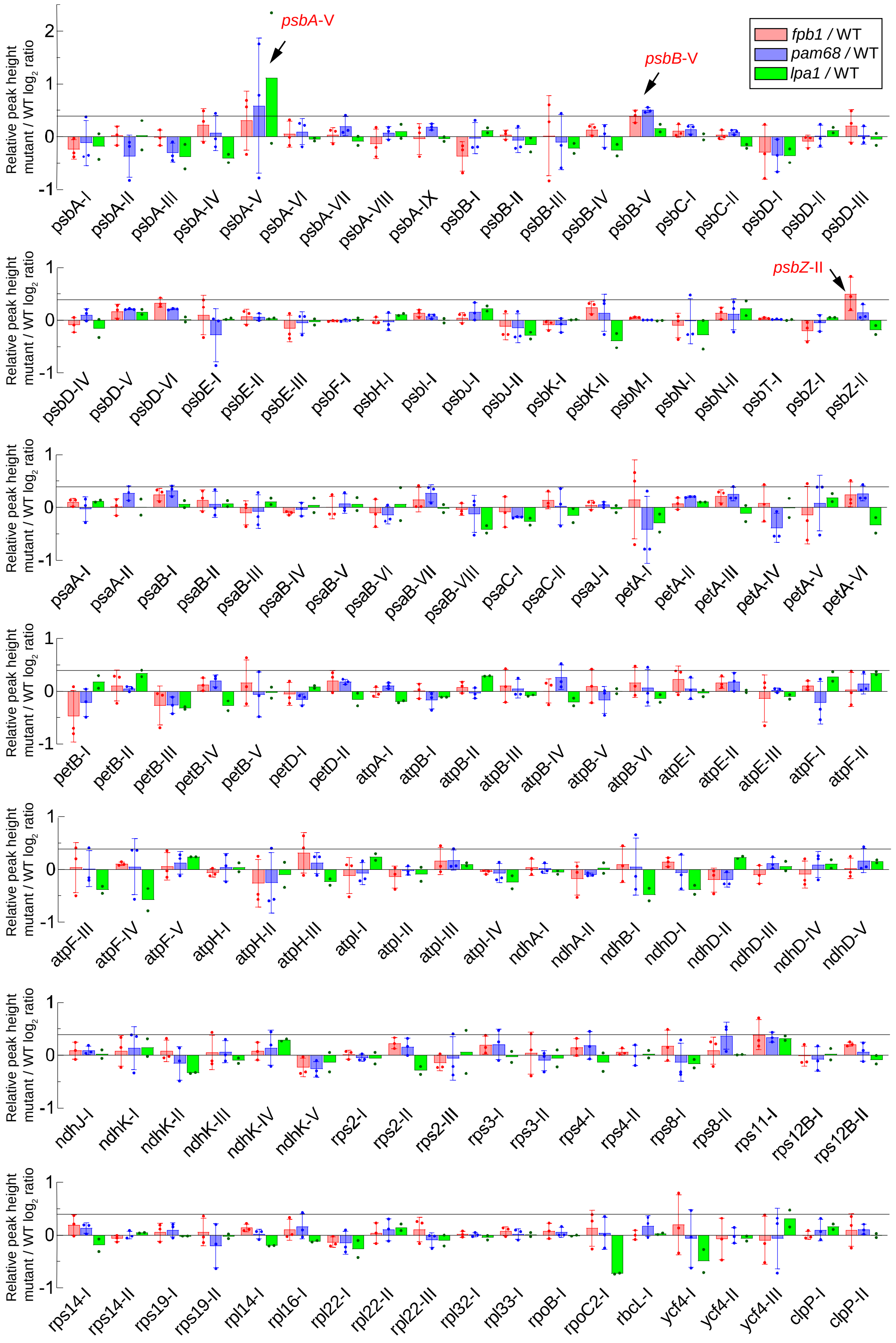

Supplementary Fig. 9 Chloroplast genome-wide analysis of ribosome pausing between the different mutants and WT. A threshold was set in this analysis, in which the ribosome footprint reads at each position along the ORF less than 500 (38

chloroplast genes) were excluded. For the remaining 50 chloroplast genes, ribosome footprints of each gene from three replicates of WT, *fpb1*, *pam68* and two replicates of *lpa1* were normalized based on the total reads (Supplementary Data 3). The relative heights of the main peaks of each gene of the mutants compared with WT were calculated and plotted in a $\log_2$ scale on the Y-axis. The numbers of major peaks of each gene are indicated as Roman numerals. Values are the means ± SD from three independent replicates of *fpb1* and *pam68* samples and the means from two replicates of *lpa1*. Lines with the value corresponding to the relative height of *psbB*-V in *fpb1* are shown. Source data are provided as a Source Data file.

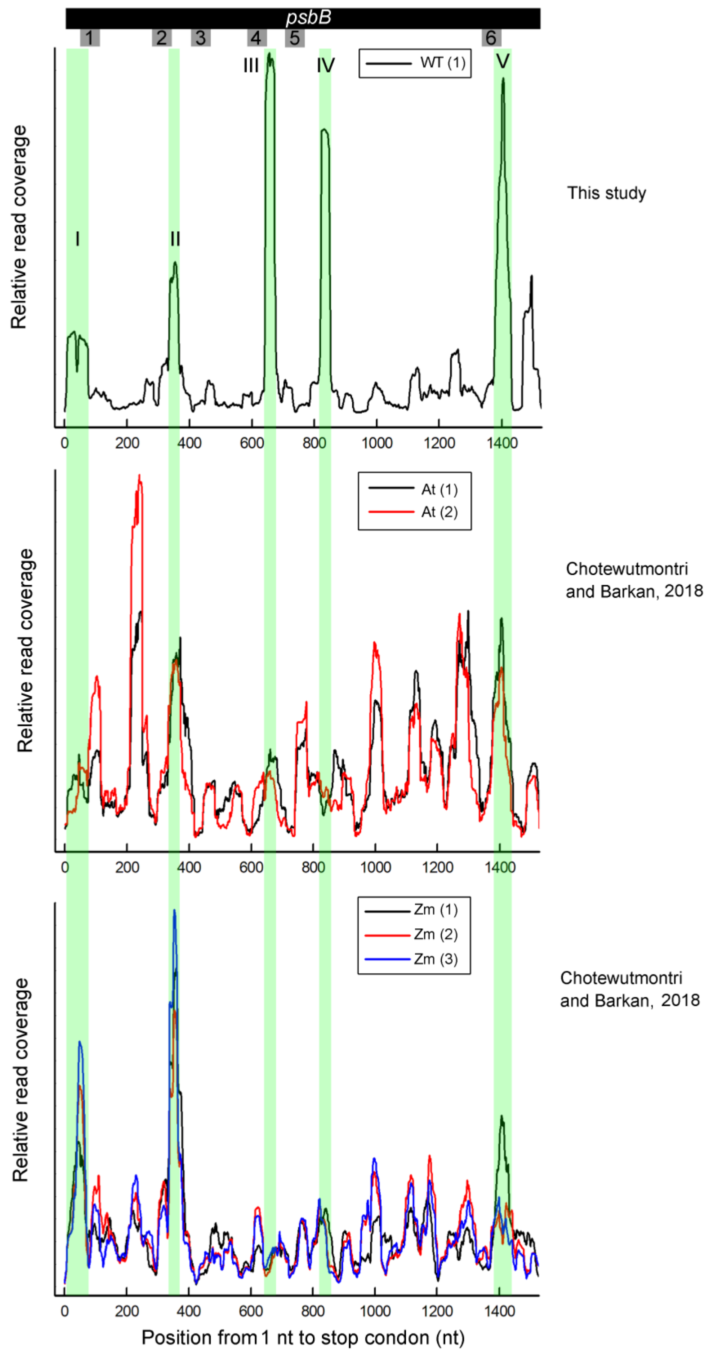

Supplementary Fig. 10 Comparison of distribution of ribosome footprints along the *psbB* ORF in Arabidopsis and maize. Distribution of ribosome footprints along the *psbB* ORF of WT (1) in this study is compared with that of Arabidopsis and maize reported by Chotewutmontri and Barkan[2]. Ribosome footprints of *psbB* from two Arabidopsis and three maize samples were normalized based on the total reads, respectively, and the relative read coverage is shown. Transmembrane domain (TMD)-encoding positions on the *psbB* mRNA are indicated from 1 to 6. Five peaks from I to V are indicated with light green shadows. Accession number for At(1), At(2), Zm(1), Zm(2) and Zm(3) are SRX3744028, SRX3744024, SRX3744002, SRX3744022 and SRX3744035, respectively. Source data are provided as a Source Data file.

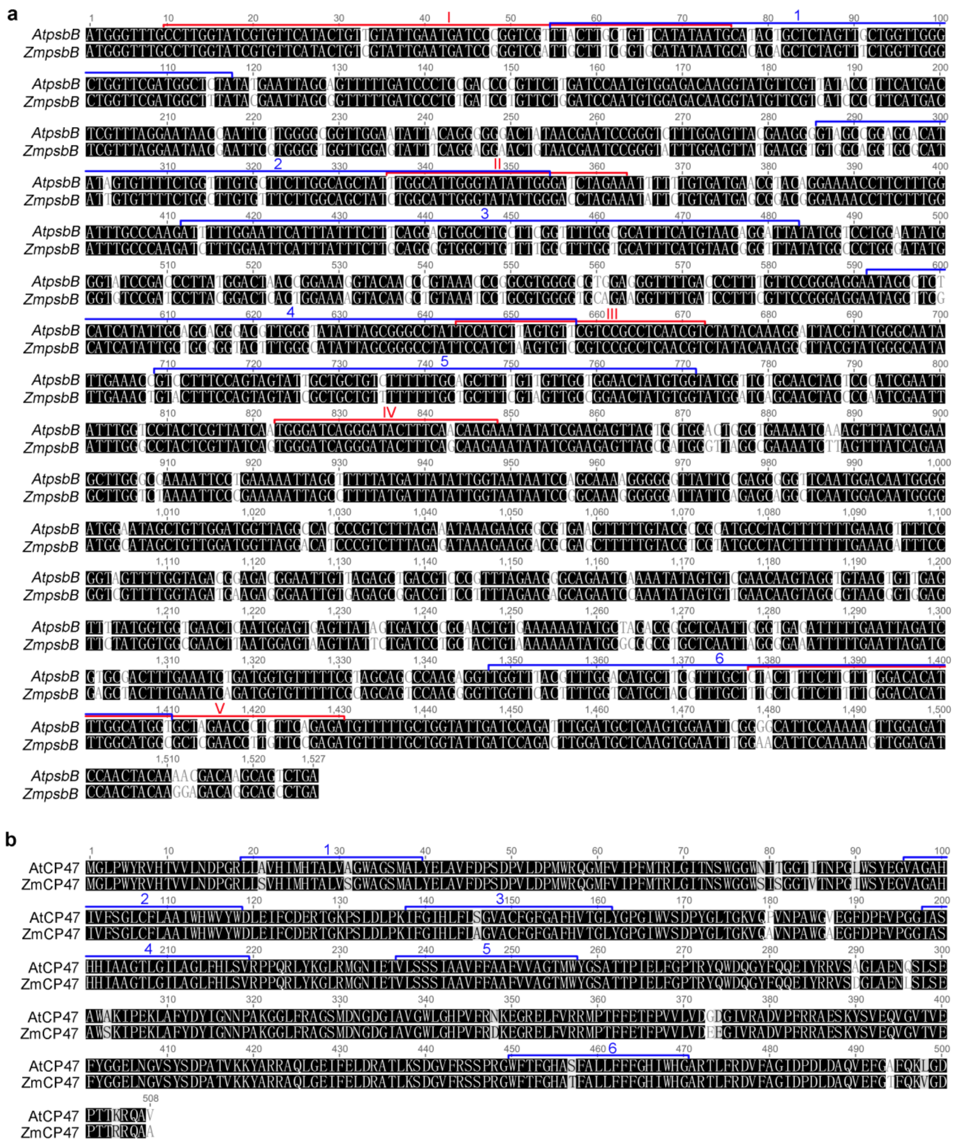

Supplementary Fig. 11 Sequence alignment of CP47 and *psbB* of Arabidopsis and maize. a Sequence alignment of *psbB* CDS from Arabidopsis and maize. The sequence of *AtpsbB* (*ATCG00680*) and *ZmpsbB* (*GRMZM5G808939_P01*) were aligned on Geneious 6.1.8 using parameters 65% similarity and global alignment with free end gaps. The sequences of *AtpsbB* and *ZmpsbB* share 87.8% sequence identity. **b** Alignment of CP47 from Arabidopsis and maize. The sequence of AtCP47 (ATCG00680) and ZmCP47 (GRMZM5G808939_P01) were aligned on Geneious 6.1.8 using Blosum62 and global alignment with free end gaps. AtCP47 shares 96.1% amino acid sequence identity with ZmCP47. Regions of the ribosome footprint peaks are indicated with red lines from I to V. The transmembrane domains (TMDs) are indicated with blue lines from 1 to 6.

Supplementary Table 1 Primers used in this work

Primer names	Sequences 5' to 3'
**Primers used for detection of T-DNA insertion sites**	
FPB1-TDNA-F	CAAAAACTATAACGTGTGGCTGC
FPB1-TDNA-R	CAGAAGTGGGGATTCCAGTAAG
PAM68-TDNA-F	CGAACTGGATTAAAGCGGTATT
PAM68-TDNA-R	GGTTTGTTTTTTGCGTGTGTG
LPA1-TDNA-F	AGATATTGGCGATAATTTCCG
LPA1-TDNA-R	CTCTACTCAATTCTAAGGTTTTACG
CtpA-TDNA-F	CCGAGATTGATGAACTAATGAG
CtpA-TDNA-R	ATGGTGGTGAATCAGTGGTAAC
SAIL-LB2	GCTTCCTATTATATCTTCCCAAATTACCAATACA
SALK-LBa1	TGGTTCACGTAGTGGGCCATCG
TLB2	ATAATAACGCTGCGGACATCTACATTTT
**Primers used for complementation vector construction**	
FPB1-com-F	CAATCTAGAGAAGATTCCTACTCCAAAACAACC
FPB1-com-R	CAACTCGAGTTAAAGAAAGCCATCTCTTTTATC
**Primers used to prepare hybridization probes for RNA gel blot analyses**	
psbA-F	TTATCCATTTGTAGATGGAGCCTCA
psbA-R	ATGACTGCAATTTTAGAGAGACGCG
psbB-F	GCAAGGATCCATGGGTTTGCCTTGG
psbB-R	GCAACTCGAGATCAGACTGCTTGTCG
psbC-F	GCGGGATCCATGAAAACCTTATATTCC
psbC-R	GGCCTCGAGTTAGTTAAGAGGAGTCATG
psbD-F	TGTTCGGAAATGGTTGAAGTAGATG
psbD-R	GGTAGAACCTCCTCAGGGAATATAA
psbEFLJ-F	CGCCTCGAGAATTTCTACAGGGATG
psbEFLJ-R	GCGGGATCCATGTCTGGAAGCACAGGAG
psbKI-F	CCGCTCGAGTATCCACAAGAACACC
psbKI-R	CTTGGATCCTTATTCTTCACGTCCCGG
psbH-F	ATGGCTACACAAACTGTTGAAG
psbH-R	GAAATTCCATCCAGTAGAACAG
ACT7-F	GGTGTCATGGTTGGTATGGGTC
ACT7-R	CCTCTGTGAGTAGAACTGGGTGC
**Primers used for protein expression vectors construction**	
FPB1-GFP-F	TAATCTAGAATGGCCGCGTCTCTAACATCTC
FPB1-GFP-R	AAGGGATCCAAAGAAAGCCATCTCTTTTATCTTG
FPB1-pET28a-ab1-F	CATGAATTCGCTGGGAGGAGGAAGGGTC
FPB1-pET28a-ab1-R	AAACTCGAGAACCTCAAAGCTCTCTTGGG
FPB1-pET28a-ab2-F	CATGGATCCGCTGGGAGGAGGAAGGGTC
FPB1-pET28a-ab2-R	AAAGAATTCAACCTCAAAGCTCTCTTGGG
Alb3-pET28a-F	TGGGGATCCAATAATGTACTTAGTACCGCCG
Alb3-pET28a-R	TTACTCGAGTACAGTGCGTTTCCGCTTCGATC
**Primers used for vector construction of split-ubiquitin assay**	

51510-NCW-F	TTACTGCAGGGCTGGGAGGAGGAAG
51510-NCW-R	GCACCATGGTTAAAGAAAGCCATCTC
PAM68-NCW-F	TTACTGCAGAGATAAAACGAAGAGTC
PAM68-NCW-R	GCACCATGGCTATCTCTTGTCTGAG
51510-Nx-F	AGTGGATCCGCTGGGAGGAGGAAG
51510-Nx-R	GCTGAATTCTTAAAGAAAGCCATCTC
PAM68-Nx-F	AGTGGCCATTACGGCCGATAAAACGAAGAGTC
PAM68-Nx-R	GCTGGCCGAGGCGGCCCTATCTCTTGTCTGAG
PsbB-Nx-F	ATGGATCCGGTTTGCCTTGGTATCGTGTTC
PsbB-Nx-R	ATCTCGAGTCAGACTGCTTGTCGTTTTGTA
PsbC-Nx-F	ATGGATCCAAAACCTTATATTCCCTGAGGAG
PsbC-Nx-R	GCGAATTCGCTTAGTTAAGAGGAGTCATGGAA
LPA1-Nx-F	ATGGATCCGATGCTCTTGTTCAGTTTGA
LPA1-Nx-R	GCGAATTCGCTCATCTTTCTAACTTGCTGAGA
LPA2-Nx-F	ATGGATCCTCAAAGAATTCAAGCTCTTCCG
LPA2-Nx-R	ATCTCGAGTCACTCTTGACCCTTCATTTTC
PsaA-Nx-sfiI-F	CGCAGAGTGGCCATTACGGCCATGATTATTCGTTCGCCGG
PsaA-Nx-sfiI-R	CTCGAGAGGCCGCCACGGCCTTATCCTACTGCAATAATTCTTGC
YCF4-2Nx-sfiI-F	GGCCATTACGGCCGTTGGAACTTCCAGTTATC
YCF4-2Nx-sfiI-R	GGCCGAGGCGGCCTTTCAAAATACTTCAATTGG
Alb3-CCW-F	ATGGCCATTACGGCCTTCAGCTTAAACGAGATTCCTC
Alb3-CCW-R	CGGGCCGAGGCGGCCAATACAGTGCGTTTCCGCTTCGATC
SCY1-CCW-F	TATGGCCATTACGGCCAGCTCTGAGGCTTCGGTTTTTG
SCY1-CCW-R	ATAGGCCGAGGCGGCCCATGGATCATACTTGTCAAGC
**Primers used for BiFC assay vector construction**	
FPB1-BiFC-F	TAAACTAGTATGGCCGCGTCTCTAAC
FPB1-BiFC-R	AGTGTCGACAAGAAAGCCATCTCTTTT
PAM68-BiFC-F	GACACTAGTATGGCTTCTGTACCATG
PAM68-BiFC-R	TATGTCGACTCTCTTGTCTGAGGA
LPA1-BiFC-F	TAAACTAGTATGGCTGTGGCTACAG
LPA1-BiFC-R	ACAGTCGACTCTTTCTAACTTGCTG
LPA2-BiFC-F	CAAACTAGTATGGCGCTACAAATCCAC
LPA2-BiFC-R	ATAGTCGACCTCTTGACCCTTCATTTTC
Alb3-BiFC-F	AGAACTAGTATGGCGAGAGTTCTAGTCTC
Alb3-BiFC-R	TTTGTCGACTACAGTGCGTTTCCG
SecY1-BiFC-F	ATCTCGAGAGCTCTGAGGCTTCGGTTTTTG
SecY1-BiFC-R	ATGGATCCTCATGGATCATACTTGTCAAGC
SecE1-BiFC-F	GAGCTCGAGACTATGACGACGAGTAATC
SecE1-BiFC-R	TTCGGATCCTCAGCTGAAGAAGTCTTGAAC
FtsY-BiFC-F	ACAACTAGTATGGCAACTTCTTCTGCTC
FtsY-BiFC-R	GCTGTCGACAGAGAATATAGCATTCAC
cpSRP54-BiFC-F	TCGGGATCCATGGAGGCTCTTCAATTTTCC
cpSRP54-BiFC-R	ATAGTCGACGTTACCAGAGCCGAAGC
SecA1-BiFC-F	CCGACTAGTATGATGGTGTCTCCACTC

SecA1-BiFC-R	ATAGGATCCGGCGCTTGCAATTGAG
YCF4-BiFC-F	TAGGGATCCATGAGTTGGCGATCAGAATC
YCF4-BiFC-R	CCGCTCGAGAAATACTTCAATTGGTACACG
PYG7-BiFC-F	GCCGGATCCATGTTCGAATCGAACATGGTTC
PYG7-BiFC-R	GCCCTCGAGCCGTTTCTTAGACTTAAC
RbcS-TP-BiFC-F	TGCTCTAGAATGGCTTCCTCTATGCTCTCTTCC
RbcS-TP-BiFC-R	CGGGGTACCAGGCCACACCTGCATGCAG

Supplementary References

1. Wei, X. Su, X. Cao, P. Liu, X. Chang, W. Li, M. Zhang, X.Z. & Liu, Z. Structure of spinach photosystem II–LHCII supercomplex at 3.2 Å resolution. Nature **534,** 69–74 (2016).

2. Chotewutmontri P. & Barkan A. Multilevel effects of light on ribosome dynamics in chloroplasts program genome-wide and *psbA*-specific changes in translation. PLOS Genetics, **14,** e1007555 (2018).
