# Supplementary material for: Thylakoid protein FPB1 synergistically cooperates with PAM68 to promote CP47 biogenesis and Photosystem II assembly
Source: Nat Commun. 2024 Apr 10;15:3122. doi: 10.1038/s41467-024-46863-y (PMC11006888; doi:10.1038/s41467-024-46863-y)
Supplement: Supplementary file 3 — Description of Additional Supplementary Files [file 41467_2024_46863_MOESM3_ESM.pdf]

Description of Additional Supplementary Files

Supplementary Data Legends

Supplementary Data 1: Summary for the proteins detected in the Co-IP samples by mass spectrometry.

Supplementary Data 2: RNA-seq and Ribo-seq read counts statistics.

Supplementary Data 3: Genome-wide analysis of the ribosome footprints distribution along the chloroplast ORFs in different mutants and WT
