# Supplementary material for: Thylakoid protein FPB1 synergistically cooperates with PAM68 to promote CP47 biogenesis and Photosystem II assembly
Source: Nat Commun. 2024 Apr 10;15:3122. doi: 10.1038/s41467-024-46863-y (PMC11006888; doi:10.1038/s41467-024-46863-y)
Supplement: Supplementary file 7 — Reporting Summary [file 41467_2024_46863_MOESM7_ESM.pdf]

nature portfolio

Corresponding author(s): Lianwei Peng

Last updated by author(s): Feb 27, 2024

Reporting Summary

Nature Portfolio wishes to improve the reproducibility of the work that we publish. This form provides structure for consistency and transparency in reporting. For further information on Nature Portfolio policies, see our Editorial Policies and the Editorial Policy Checklist.

Statistics

For all statistical analyses, confirm that the following items are present in the figure legend, table legend, main text, or Methods section.

n/a	Confirmed	
☐	☒	The exact sample size (*n*) for each experimental group/condition, given as a discrete number and unit of measurement
☐	☒	A statement on whether measurements were taken from distinct samples or whether the same sample was measured repeatedly
☒	☐	The statistical test(s) used AND whether they are one- or two-sided<br>*Only common tests should be described solely by name; describe more complex techniques in the Methods section.*
☒	☐	A description of all covariates tested
☒	☐	A description of any assumptions or corrections, such as tests of normality and adjustment for multiple comparisons
☐	☒	A full description of the statistical parameters including central tendency (e.g. means) or other basic estimates (e.g. regression coefficient) AND variation (e.g. standard deviation) or associated estimates of uncertainty (e.g. confidence intervals)
☒	☐	For null hypothesis testing, the test statistic (e.g. *F*, *t*, *r*) with confidence intervals, effect sizes, degrees of freedom and *P* value noted<br>*Give P values as exact values whenever suitable.*
☒	☐	For Bayesian analysis, information on the choice of priors and Markov chain Monte Carlo settings
☒	☐	For hierarchical and complex designs, identification of the appropriate level for tests and full reporting of outcomes
☒	☐	Estimates of effect sizes (e.g. Cohen's *d*, Pearson's *r*), indicating how they were calculated

Our web collection on statistics for biologists contains articles on many of the points above.

Software and code

Policy information about availability of computer code

Data collection	Western blot and RNA gel blot signal were visualized with ImageSaver6 LuminoGraph.<br>Chlorophyll fluorescence and P700 were measured with ImagingWin v2.40b, PamWin v3.22d and Dual PAM v1.19.<br>RNA-Seq and Ribo-seq were sequenced with Illumina HiSeqTM X10 and data were collected with bcl2fastq (v2.20.0.422).<br>Fluorescence signals were observed with FV31S-SW v2.3.2.169.<br>Raw datas of MS/MS spectra were collected by Q Exactive.
Data analysis	Data analyses were done with Microsoft Excel and graph data analyses were done with OriginPro 2019b and GraphPad Prism 10.1.2.<br>Ribo-Seq and RNA-Seq data analysis were done with fastP v0.18.0, Bowtie2 v2.2.8, STAR v2.5.1b, RSEM v1.2.19, StringTie v1.3.1 and HISAT v2.2.4.<br>Sequences alignment was done with DNAMAN 8 and Geneious R6.<br>Raw data of MS/MS spectra were analyzed with maxquant software (version 2.3).<br>Image processing was performed by Photoshop 7.0 and Adobe Illustrator 2023.

For manuscripts utilizing custom algorithms or software that are central to the research but not yet described in published literature, software must be made available to editors and reviewers. We strongly encourage code deposition in a community repository (e.g. GitHub). See the Nature Portfolio guidelines for submitting code & software for further information.

Data

Policy information about availability of data

All manuscripts must include a data availability statement. This statement should provide the following information, where applicable:

- Accession codes, unique identifiers, or web links for publicly available datasets
- A description of any restrictions on data availability
- For clinical datasets or third party data, please ensure that the statement adheres to our policy

All data supporting the findings of this study are available within the paper and the Supplementary Information. All other data that support the findings of this study are available from the corresponding author upon request. The raw data of RNA-Seq and ribosome profiling generated in this study have been deposited in the GenBank (NCBI) Sequence Read Archive (SRA) with accession number PRJNA948060 (https://www.ncbi.nlm.nih.gov/sra/PRJNA948060). The MS raw data are available through the MassIVE repository with accession ID: MSV000094131 (https://massive.ucsd.edu/ProteoSAFe/dataset.jsp?task=c74dd58b934141f6a87b92f496983664). Source data are provided with this paper.

Research involving human participants, their data, or biological material

Policy information about studies with human participants or human data. See also policy information about sex, gender (identity/presentation), and sexual orientation and race, ethnicity and racism.

Reporting on sex and gender	N/A
Reporting on race, ethnicity, or other socially relevant groupings	N/A
Population characteristics	N/A
Recruitment	N/A
Ethics oversight	N/A

Note that full information on the approval of the study protocol must also be provided in the manuscript.

Field-specific reporting

Please select the one below that is the best fit for your research. If you are not sure, read the appropriate sections before making your selection.

☒ Life sciences ☐ Behavioural & social sciences ☐ Ecological, evolutionary & environmental sciences

For a reference copy of the document with all sections, see nature.com/documents/nr-reporting-summary-flat.pdf

Life sciences study design

All studies must disclose on these points even when the disclosure is negative.

Sample size	No sample size was performed. In this study, we used pools of at least 100 seedlings and 6-10 plants per each biological replicate.
Data exclusions	No data were excluded from the analyses.
Replication	For RNA-Seq and Ribo-Seq analyses, the experiments were conducted with 2 (lpa1) and 3 (WT, fpb1, and pam68) biological replicates. For immunoblots and CoIP, all experiments were performed at least twice. For RNA blots, polysome, BN-PAGE analyses, yeast two hybrid assays, BiFC and protein labelling analyses were performed twice. All independent replicates involved in the same assay produced similar results.
Randomization	N/A
Blinding	Blinding was not applicable due to the technical procedures employed.

Reporting for specific materials, systems and methods

We require information from authors about some types of materials, experimental systems and methods used in many studies. Here, indicate whether each material, system or method listed is relevant to your study. If you are not sure if a list item applies to your research, read the appropriate section before selecting a response.

Materials & experimental systems

n/a	Involved in the study
☐	☒ Antibodies
☒	☐ Eukaryotic cell lines
☒	☐ Palaeontology and archaeology
☒	☐ Animals and other organisms
☒	☐ Clinical data
☒	☐ Dual use research of concern
☐	☒ Plants

Methods

n/a	Involved in the study
☒	☐ ChIP-seq
☒	☐ Flow cytometry
☒	☐ MRI-based neuroimaging

Antibodies

Antibodies used

Commercially available antibodies were obtained from PhytoAB (USA) and are listed below: PAM68 (PHY2289A, 1:1000), D1 (PHY0103A, 1:1000), D2 (PHY0060, 1:1000), CP43 (PHY0318, 1:2000), CP47 (PHY0319, 1:2000), PsbE (PHY3491S, 1:1000), PsbI (PHY0132A, 1:5000), PsbO (PHY0344, 1:5000), PsaA (PHY0342, 1:2000), PsaD (PHY0343, 1:1000), Cyt b6 (PHY0020S, 1:1000), CF1β (PHY0312, 1:1000), CF1γ (PHY0313, 1:1000), CF1ε (PHY0315, 1:1000), SBPase (PHY0410S, 1:1000), LPA1 (PHY0481S, 1:1000), HCF244 (PHY0327, 1:1000), YCF4 (PHY1363A, 1:1000). The Antibodies against pD1 (9 amino acid residues in the C terminus of pD1, 1:5000) and CtpA (1:1000) were obtained from Prof. Aigen Fu (Northwest University, China). The antibodies of FPB1 and Alb3 were generated by PhytoAB company (USA) in rabbits. Lhcb1 was used in our previously published article (Li et al., Plant physiology, 2019).

Validation

All commercially available antibodies were validated by the supplier (https://www.phytoab.com/). Antibodies against FPB1 and Alb3 were confirmed in this study. pD1, CtpA and Lhcb1 were used in the previously published articles (Chang et al., Front Plant Sci., 2021; Che et al., PNAS, 2013; Li et al., Plant physiology, 2019).

Plants

Seed stocks

fpb1, SALK_048033; lpa1, GABI_655D01; alb3, SALK_070924; hcf173, GABI_246C02; hcf244 , GABI_088C04; pam68, isolated from a T-DNA insertion pool (Stock No.: CS31400); hcf107 (hcf107-2), hcf136, and ctpa (atctpa-1) were provided by Prof. Jörg Meurer and Aigen Fu, respectively (Felder et al., Plant Cell, 2001; Meurer et al., EMBO, 1998; Che et al., PNAS, 2013).

Novel plant genotypes

Double mutant fpb1 pam68 and fpb1 lpa1 were generated by hybridization. For complementation of the fpb1 mutant, genomic DNA fragments of wild-type At3g51510 were cloned into the pBIN19 vector. The vector was transferred into Agrobacterium tumefaciens C58C and the bacteria were used to transform fpb1 plants by the floral dipping method. At least ten complemented lines were obtained and one representative line was used for further analysis.

Authentication

Sequencing of the PCR products was used to authenticate genotypes of fpb1 and pam68.
