# Supplementary material for: Thylakoid protein FPB1 synergistically cooperates with PAM68 to promote CP47 biogenesis and Photosystem II assembly
Source: Nat Commun. 2024 Apr 10;15:3122. doi: 10.1038/s41467-024-46863-y (PMC11006888; doi:10.1038/s41467-024-46863-y)
Supplement: Supplementary file 8 — Source Data [file 41467_2024_46863_MOESM8_ESM.zip › 7-Source data/7-source data for images.pdf]

Figure 1

Figure 1a

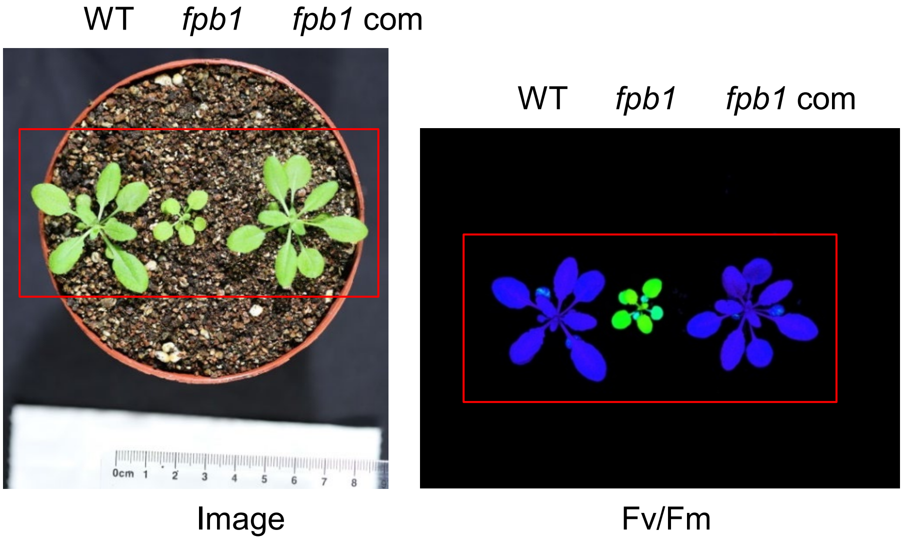

Figure 1b

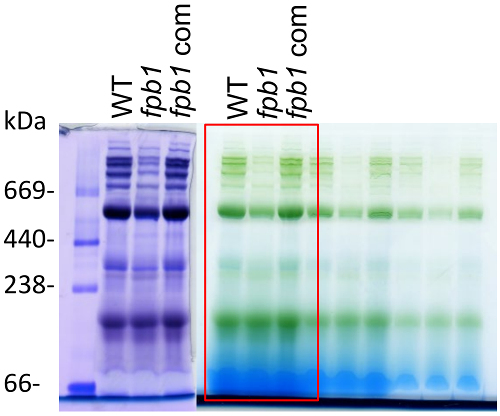

Figure 1c

Please note that: Protein ladder from ThermoFisher (Catalog Number 26616) was used throughout the whole study. An unknown band between 15 and 25 kDa was found in SDS-urea-PAGE but not in SDS-PAGE and Tricine-SDS-PAGE.

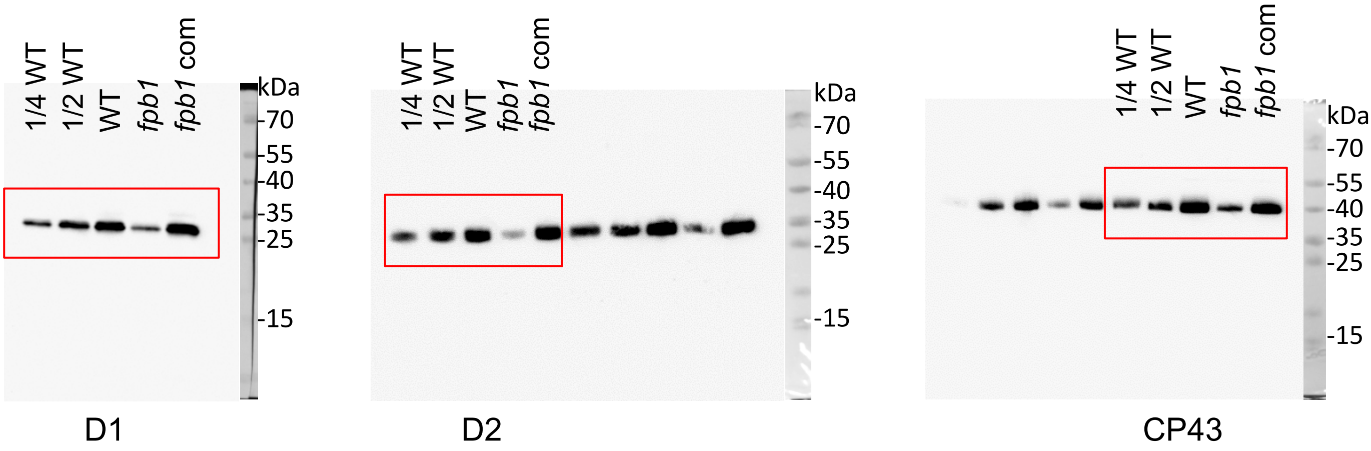

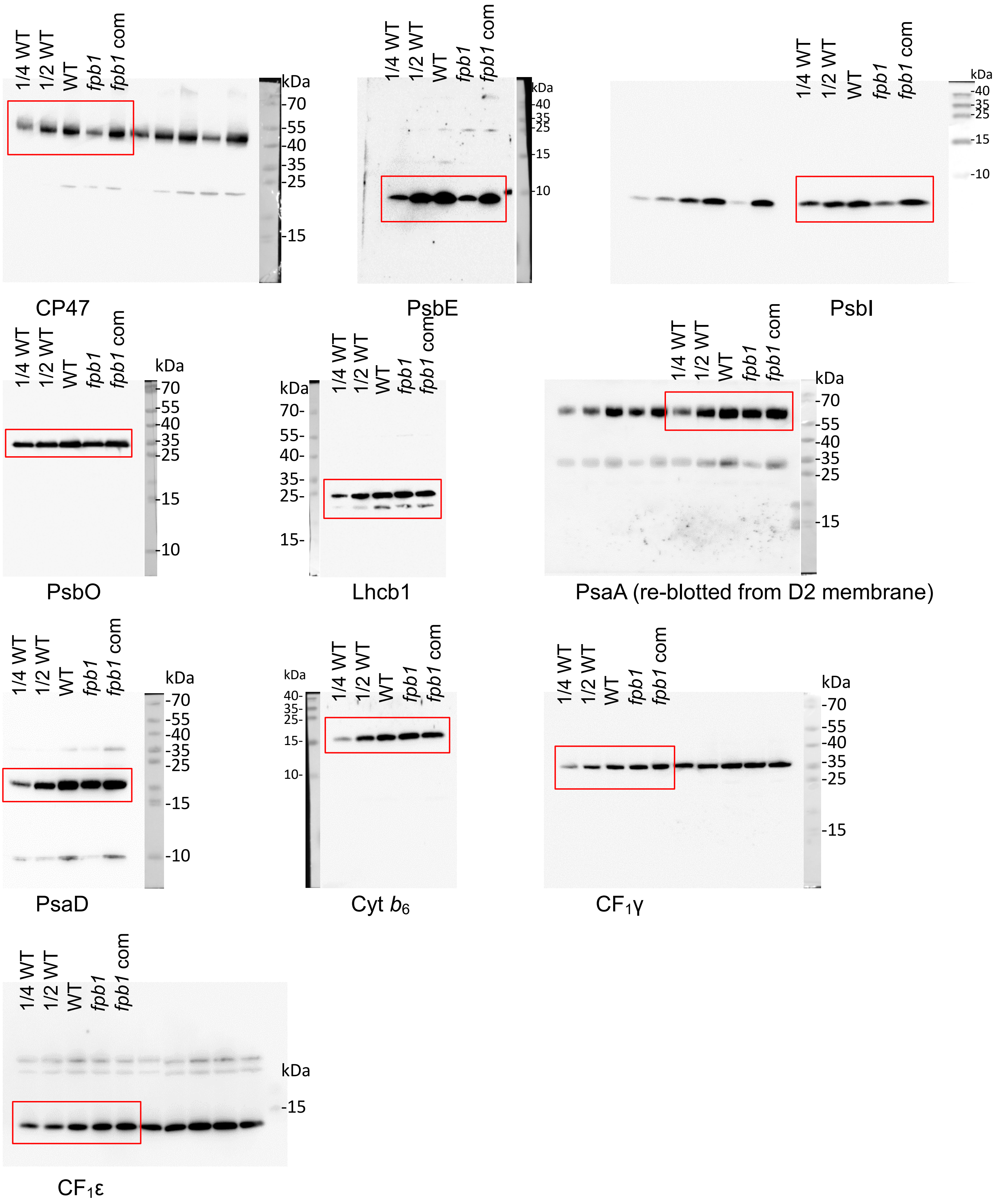
1/4 WT
1/2 WT
WT
fpb1
fpb1 com
kDa
CP47
PsbE
PsbI
PsbO
Lhcb1
PsaA (re-blotted from D2 membrane)
PsaD
Cyt b_6
$CF_1γ$
$CF_1ε$

Figure 1d

WT *fpb1* *fpb1* com

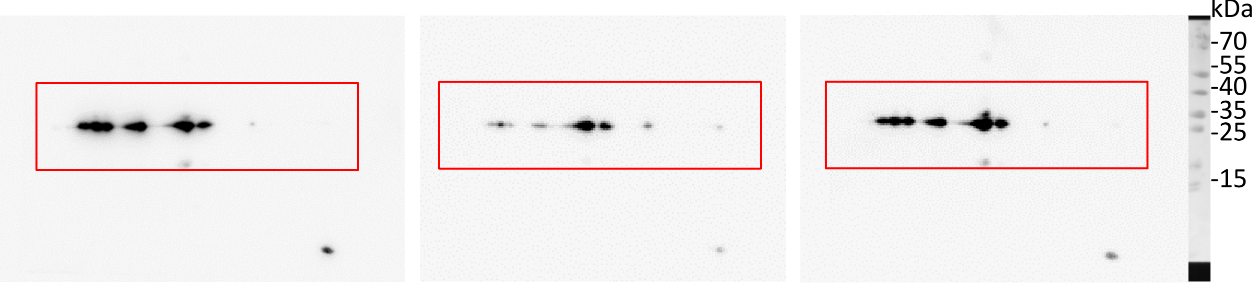

D1

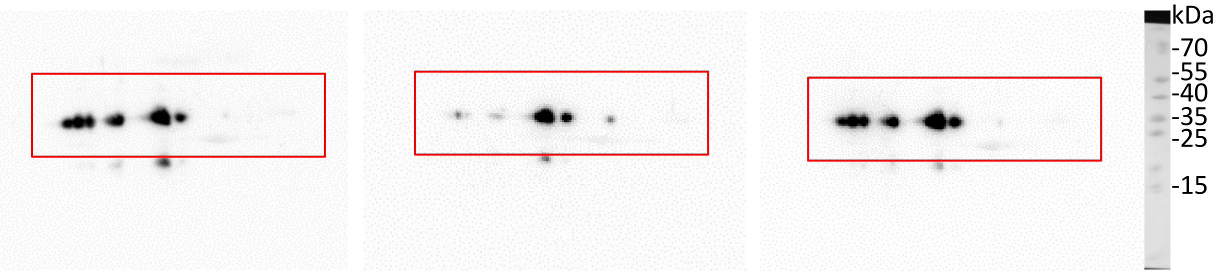

D2

WT *fpb1* *fpb1* com

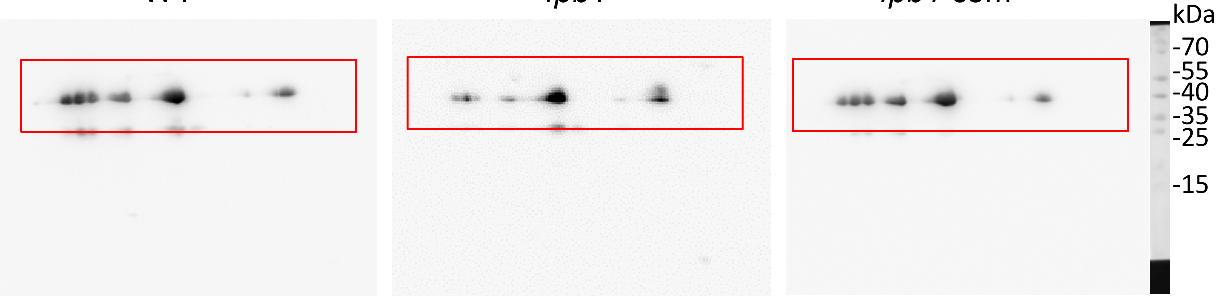

CP43 (re-blotted from D1 membrane (top))

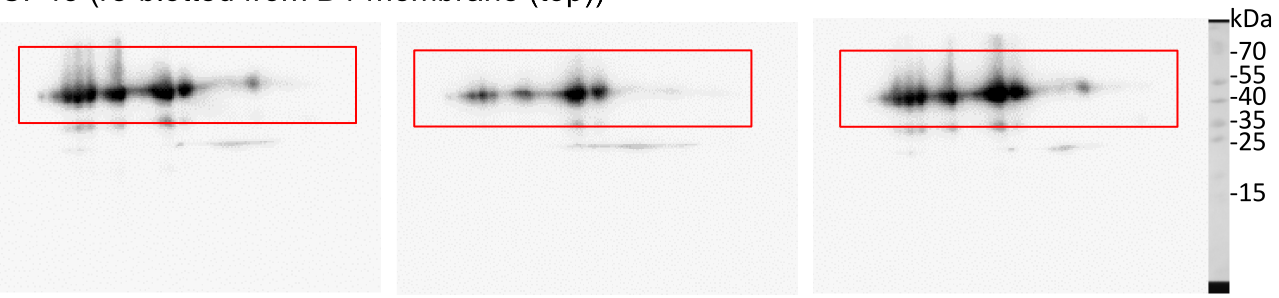

CP47 (re-blotted from D2 membrane (top))

Figure 2

Figure 2a

Left –Puromycin

WT *fpb1* *pam68*

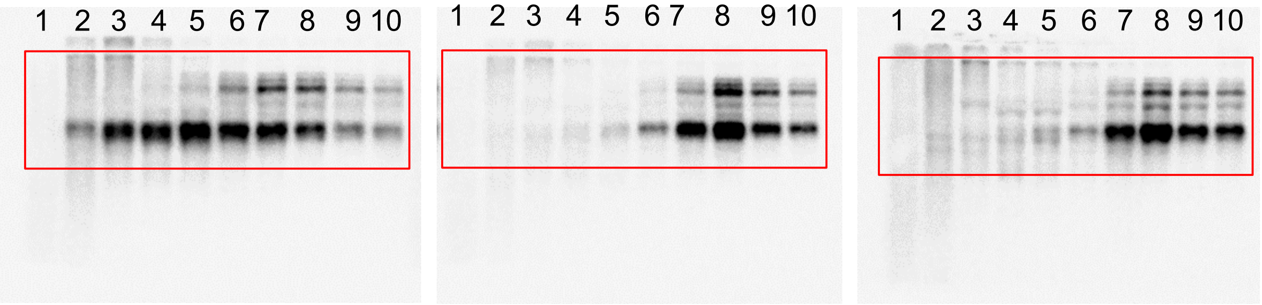

psbB

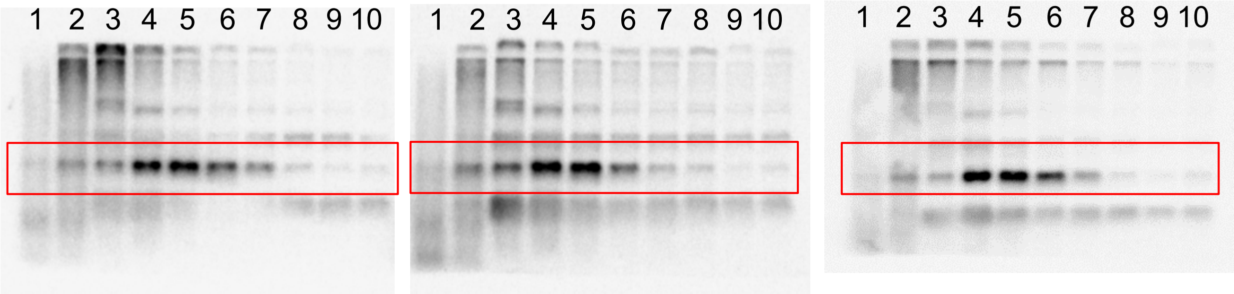

psbEFLJ

Right +Puromycin

WT *fpb1* *pam68*

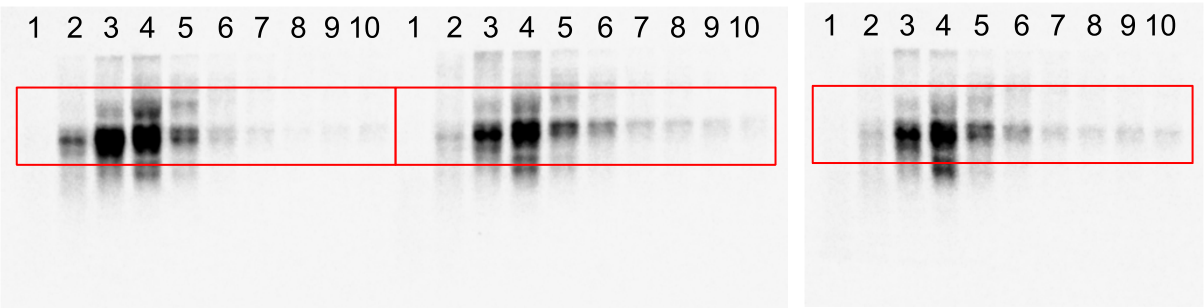

psbB

psbEFLJ

WT *fpb1*

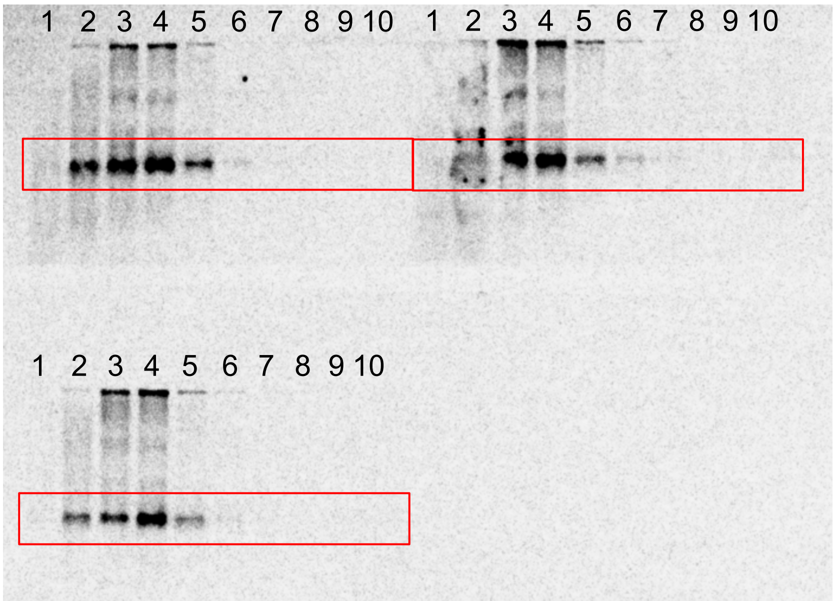

pam68

Figure 2b

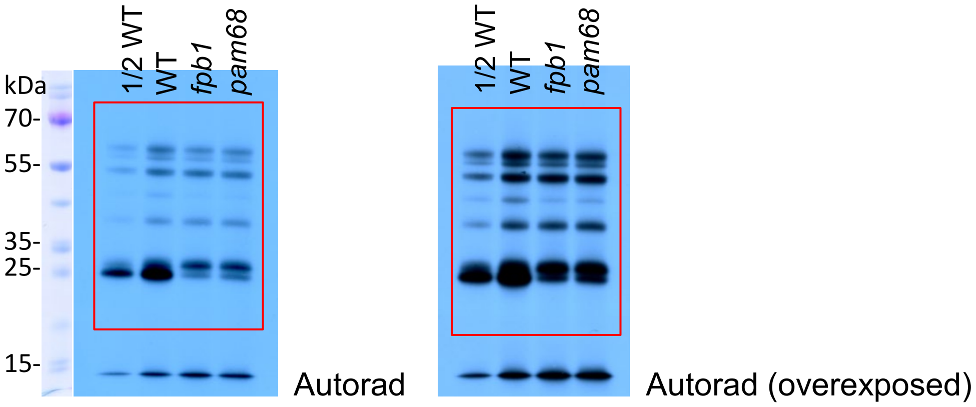

Figure 2c

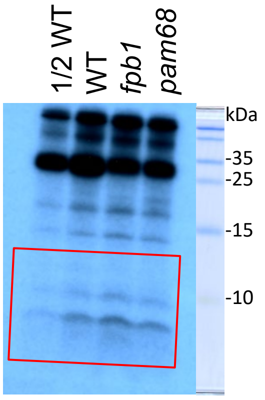

Figure 2d

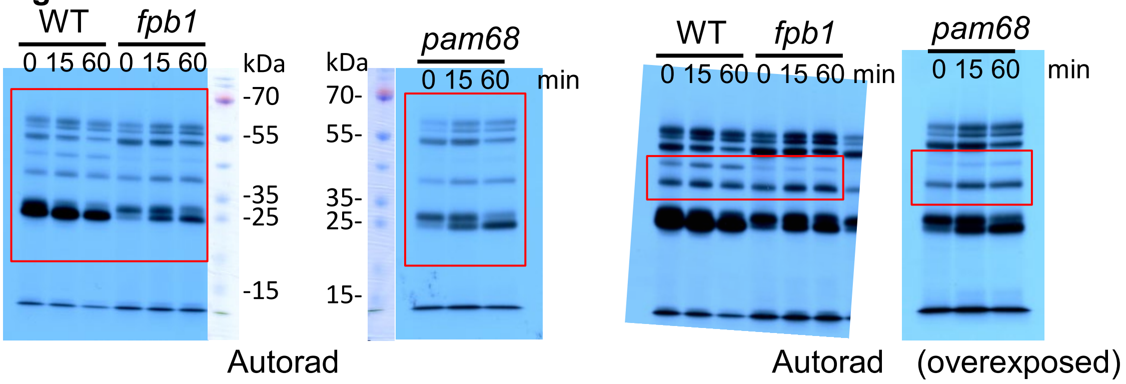

Figure 2e

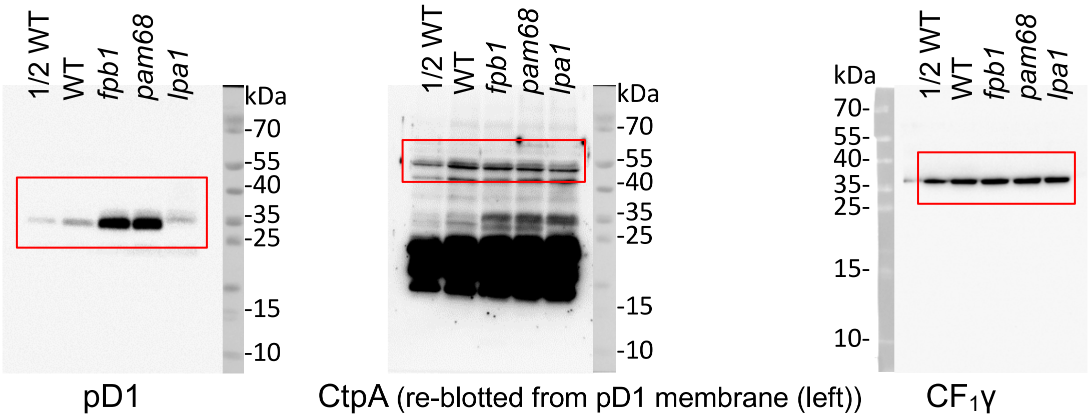

Figure 2f

WT

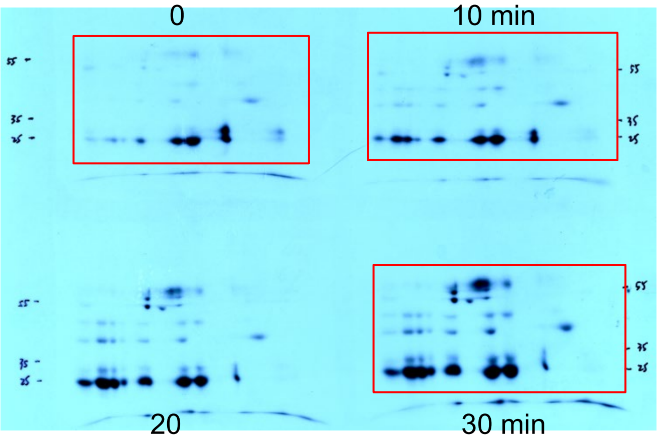

fpb1

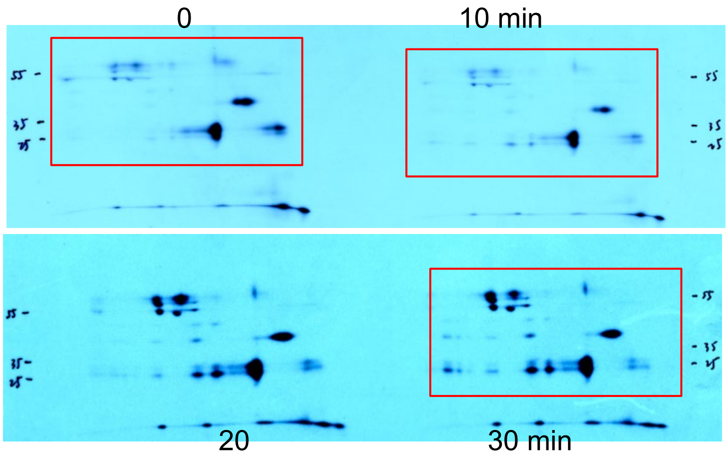

pam68

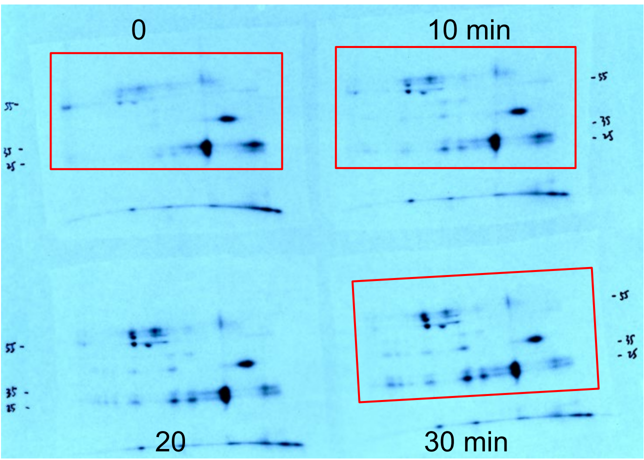

Figure 3

Figure 3a

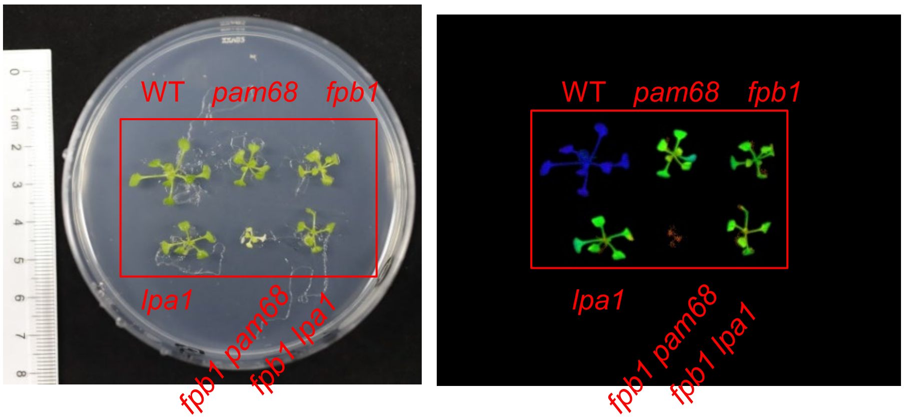

Figure 3b

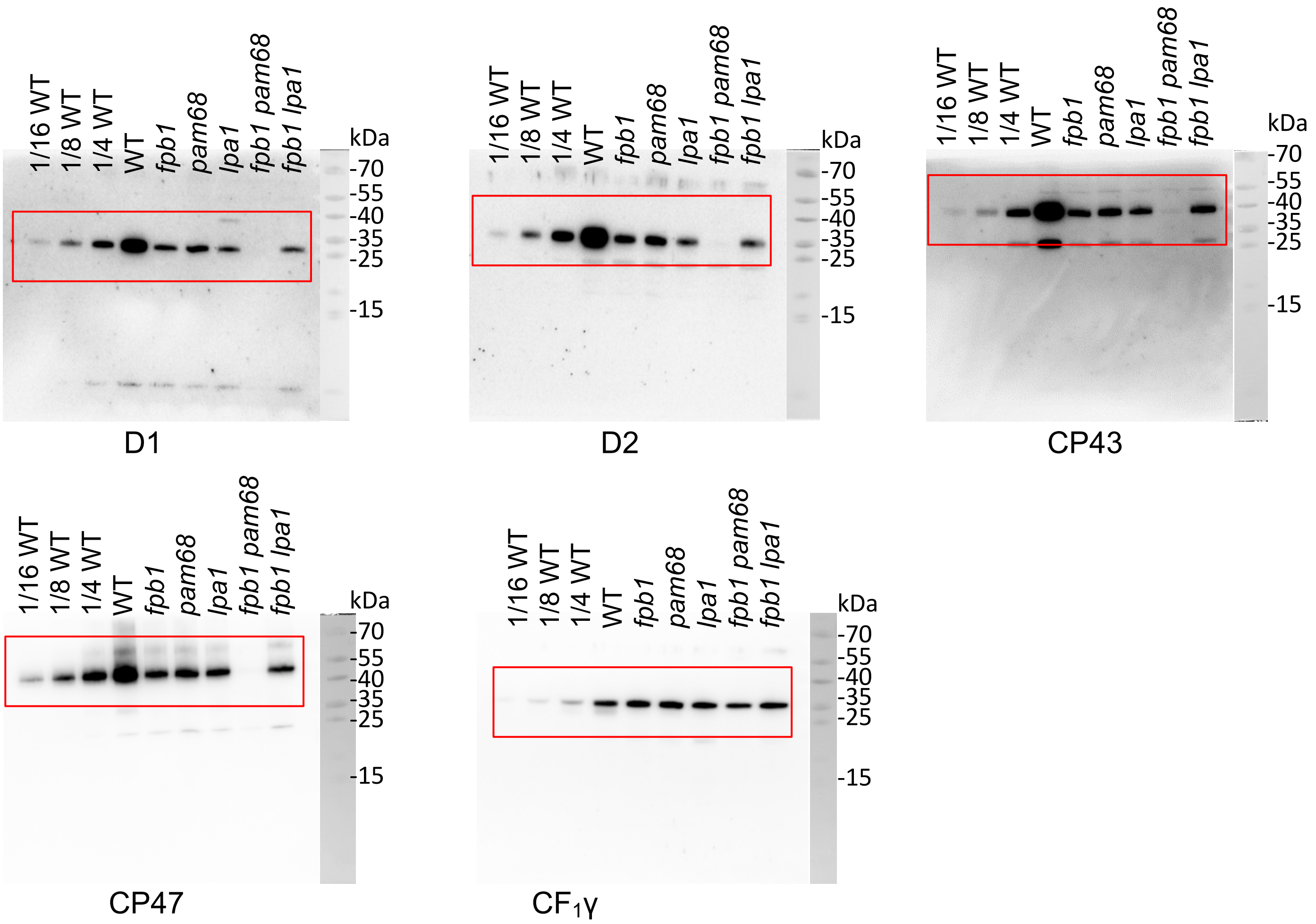

Figure 3c

D1

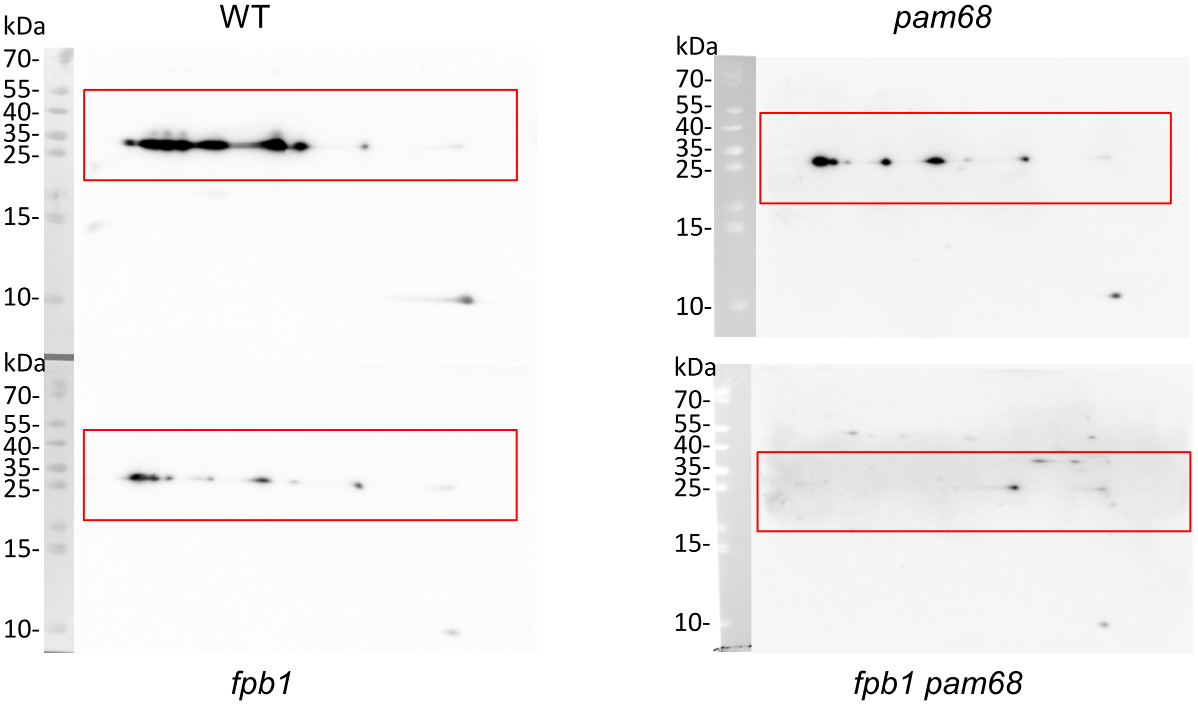

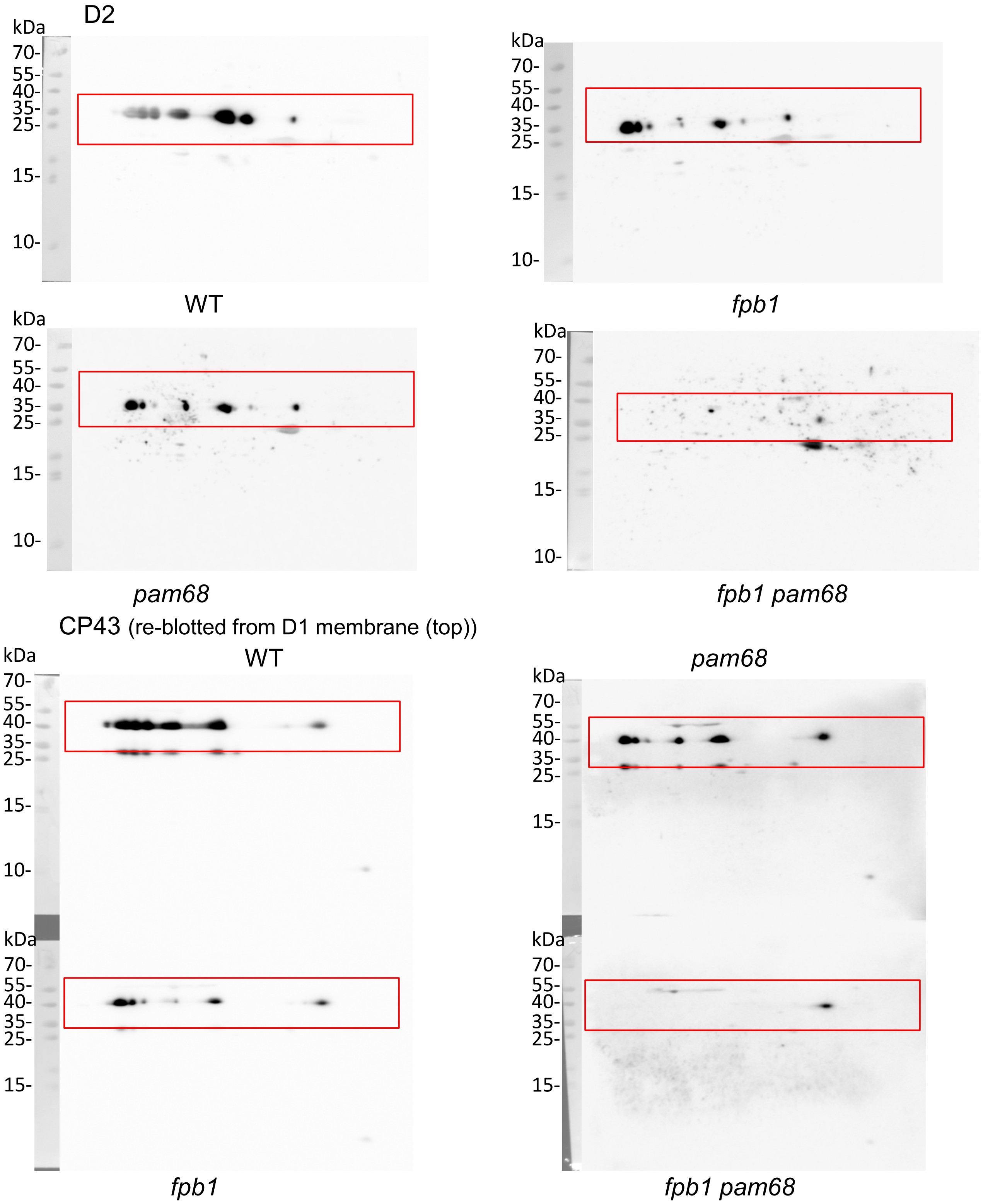
D2
kDa
70-
55-
40-
35-
25-
15-
10-
WT
fpb1
pam68
fpb1 pam68
CP43 (re-blotted from D1 membrane (top))
WT
pam68
fpb1
fpb1 pam68

CP47 (re-blotted from D2 membrane (top))

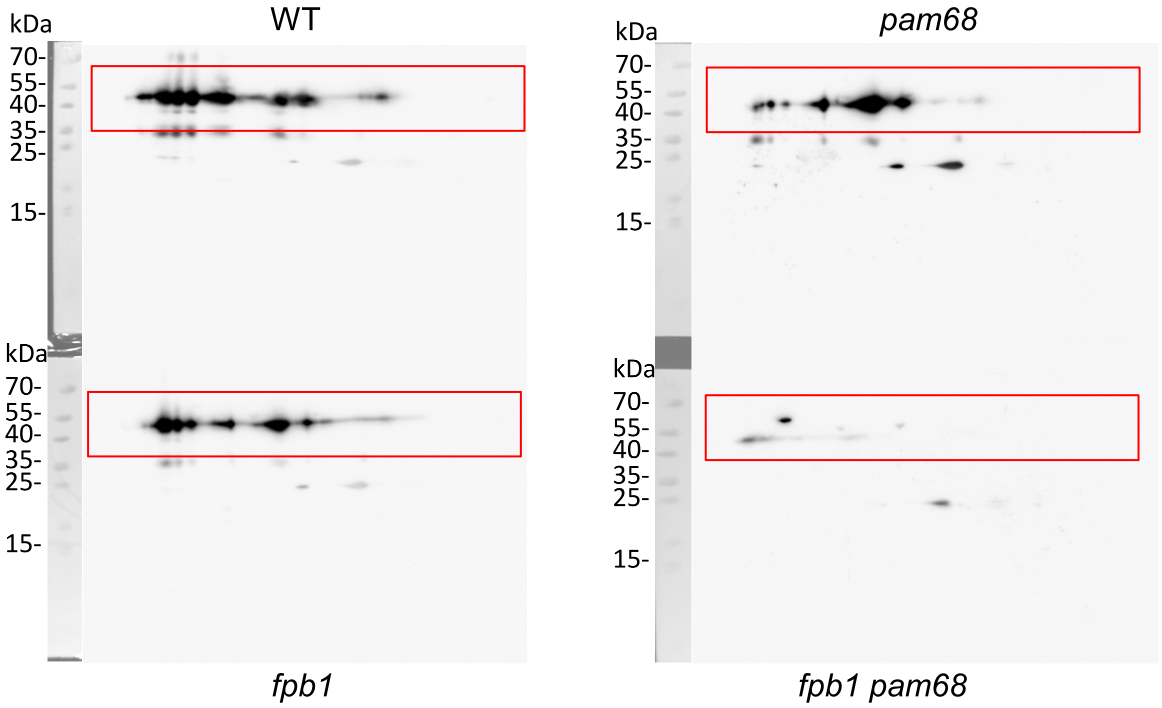

Figure 4

Figure 4a

Sequences of FPB1 and its orthologs

>AT3G51510 Arabidopsis thaliana

MAASLTSLPTGFCLSHGDECCNRSPTKSPFPGHHPLAGRRKGHLLHYERSTVRRLVVTAATEGSKKSKESEPSWANPDS
DEPPPWARNEGRSSTSQESFEVPFFVYLLASAITAIAAIGSVFEYTSKNPVFGILESDSIFYTPVLGFFALTGIPTSVFLWFKS
VEAANKEAQEQDKRDGFL

>Potri.001G455900.1 Populus trichocarpa

MAAATLLCWCNSDLIIHGSCSRLFVRNKVMDSNQSTVKSRGSYYAYQKQKKFFVCAATEGSAKSSKSEETIPSWAKPDSD
EPPPWAKGEGKENSSKQNFEVPFFVYLLASAITAIAAIGSIFEYVNQRPVFGVVNPDSIFYAPLLGFFAFTGIPFSAFLWFKSV
QAANKEAEEQDRRDGFF

>GRMZM2G117558_T01 Zea mays

MAAAAVAISTSVCTSSRRSTGCPNVRPQARPLLGMPSLRRRLQTALAATGKTPGEAEEQVPAWAKPGADEPPPWEREGG
AARGQEARQVPFYAYLLASTITAIAAIGSIFEYTNQRPVFGIVGPDSALYAPLLGFFVFTGIPTSAFLWFKAVQTANREAEEQD
RRDGFL

>LOC_Os05g41190.1 Oryza sativa

MAAAVSACSSRCLRLILGRPPRARLASVVEVVEKRRRGGLVAVAATEGSAKSSGEADEQVPSWARPGSDEPPPWAREGG
GGGGQQEPGAVELPFFAYLLASAITAIAAIGSIFEYANQRPVFGVVSPDSALYAPLLGFFVFTGIPTSGFLWFKAVQTANKEA
EEQDRRDGFS

>69021 Selaginella moellendorffii

PEWAKPGSDVPPPWASGEKKQVSSEGFQDLPYIVYLVASCLVAIAAVGSIFEYFNKNPVFGVIQPDSPFYTPVLGFFSITGIP
VSAFLWFRAIKLANKDAERQDKEDGY

>Pp3c12_25230V3.1 Physcomitrella patens

MTFAAALAASLAGPHLSIPSFSSSRTKVRAQSSSSCALPLTSRRRDIQRQWRVQSQSDQAPGSSKTEDKPTENEPTPSWA
KPGTEELPPWARNEAAAPVDSSGDLPFPVYLIGSCLVAIAAVGSIFEYFNQNPIFGVVQPDSPLWAPILGVFAITGFPSAGFL
FYKAISLANKASEEADRADGFDP

>Cre16.g679300.t1.2 Chlamydomonas reinhardtii

MCVTIGRFSSGVSARSLQTQVRAQPEQKTPPTVPGSEDELPPWVRREKERELQAKDGVSGLPWGLCLLFSVFTAIAAVGS
IFEFVDRNAIFGVIQPDSPLWAPILLFFGVTGFPTAGYLFIKGVNGFNEEAERQDKLDGYL

Prediction of transmembrane domains

AT3G51510

TMHMM result

WEBSEQUENCE Length: 181
WEBSEQUENCE Number of predicted TMHs: 2
WEBSEQUENCE Exp number of AAs in TMHs: 45.07206
WEBSEQUENCE Exp number, first 60 AAs: 0
WEBSEQUENCE Total prob of N-in: 0.97536

WEBSEQUENCE	TMHMM2.0	inside	1	101
WEBSEQUENCE	TMHMM2.0	TMhelix	102	124
WEBSEQUENCE	TMHMM2.0	outside	125	138
WEBSEQUENCE	TMHMM2.0	TMhelix	139	161
WEBSEQUENCE	TMHMM2.0	inside	162	181

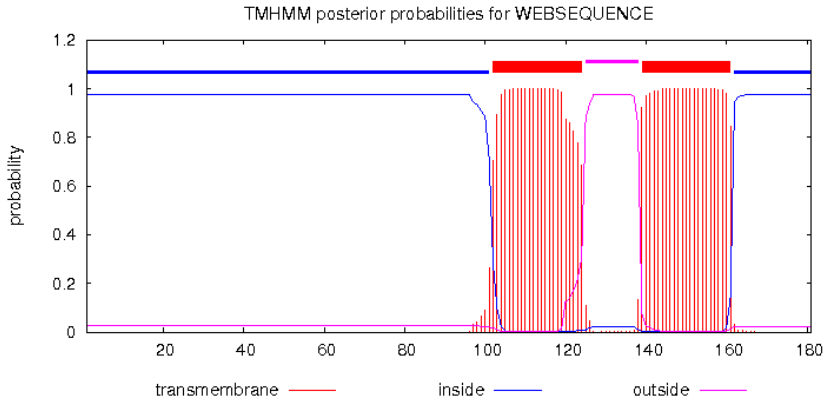

Prediction of cleavage site

AT3G51510

chlorop v1.1 prediction results

Number of query sequences: 1

Name	Length	Score	cTP	CS-score	cTP-length
Sequence	181	0.479	-	2.999	36

Figure 4b

GFP Chlorophyll Overlay

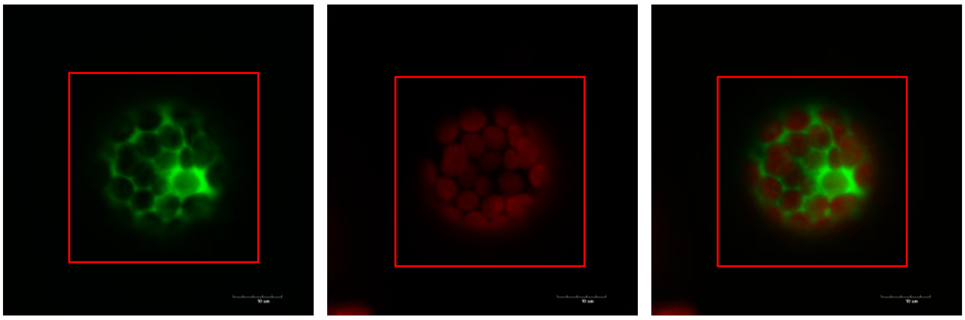

Vec-GFP

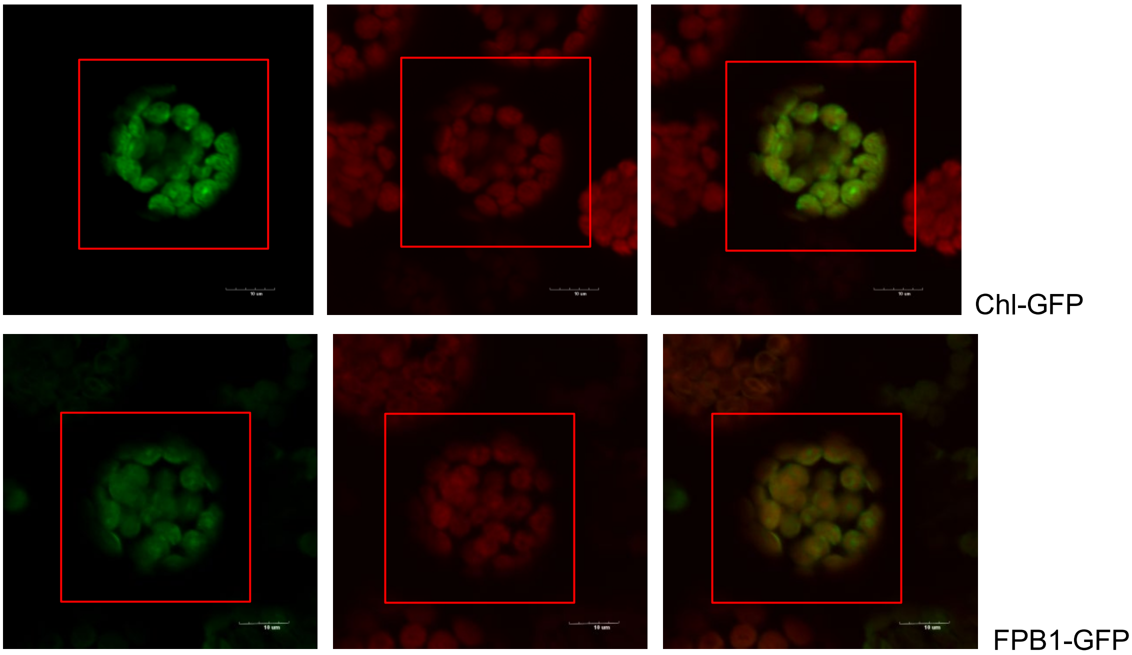

Figure 4c

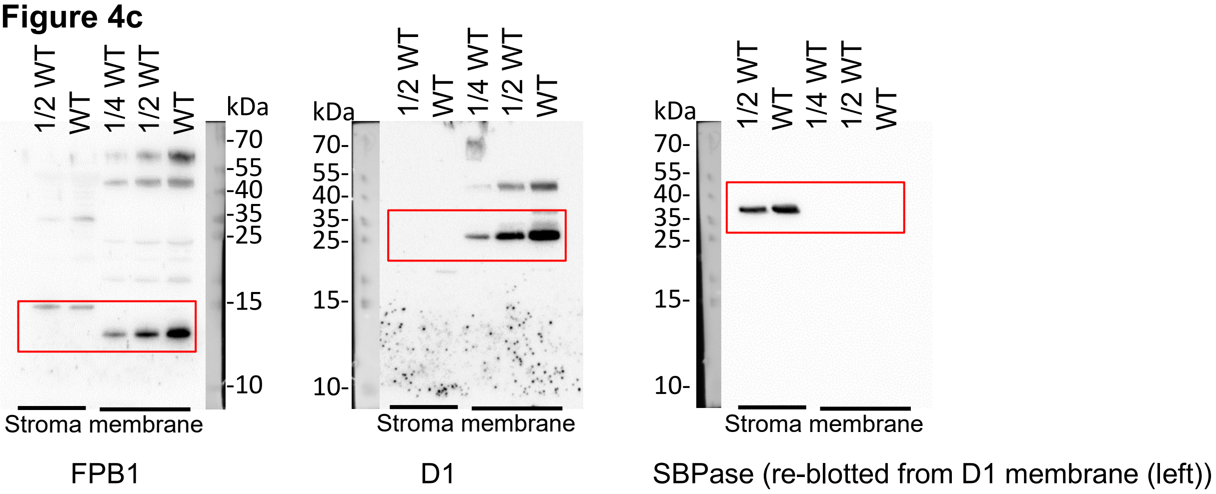

Figure 4d

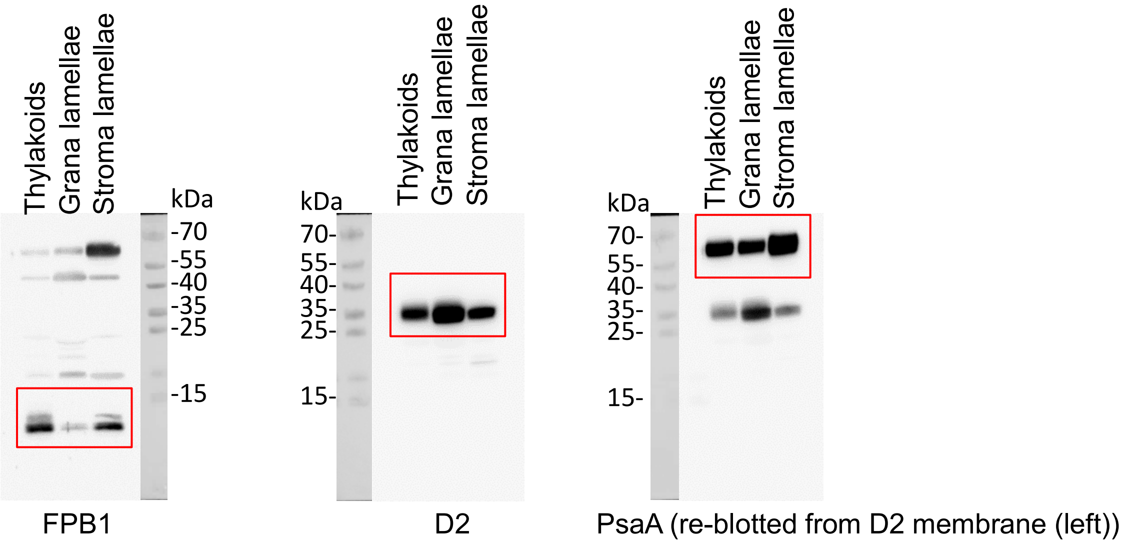

Figure 4e

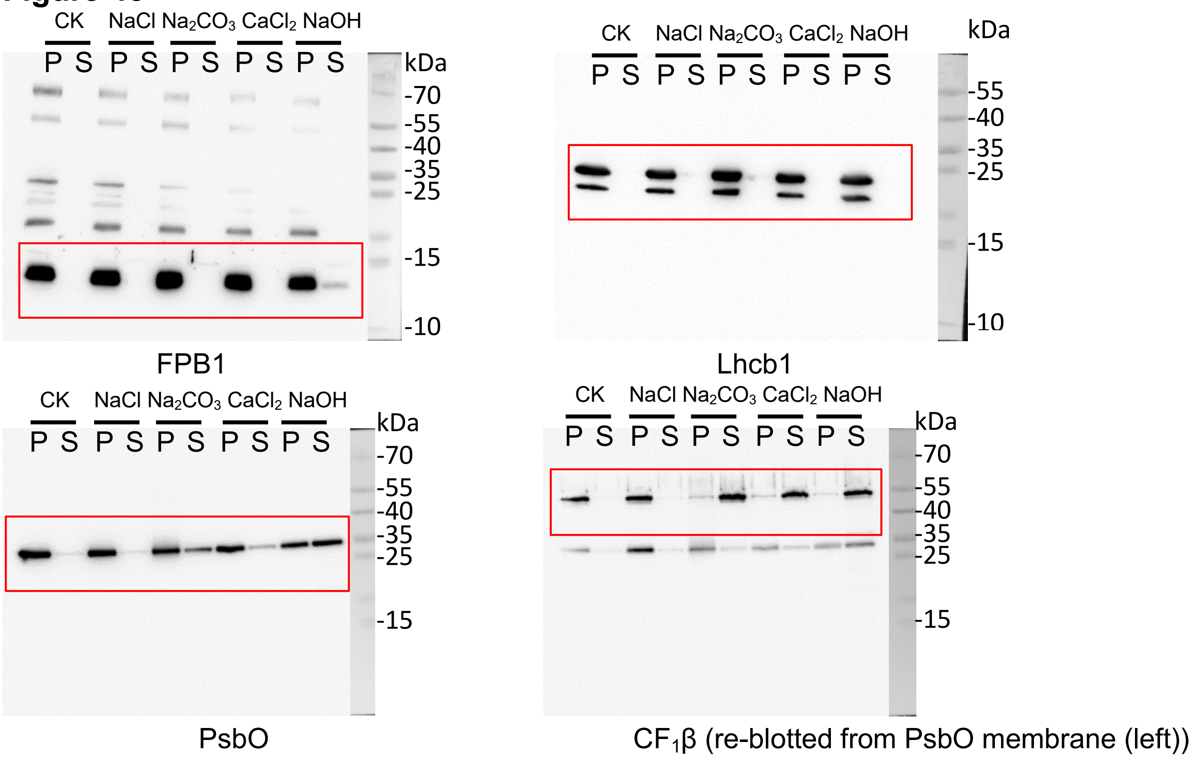

Figure 4f

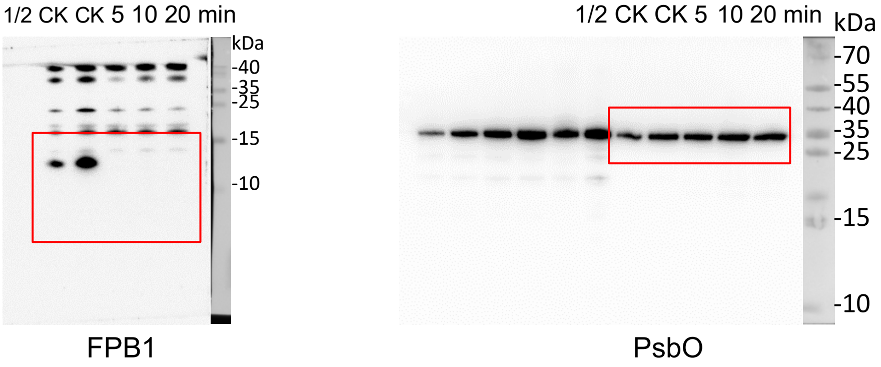

Figure 5

Figure 5a

Left FPB1

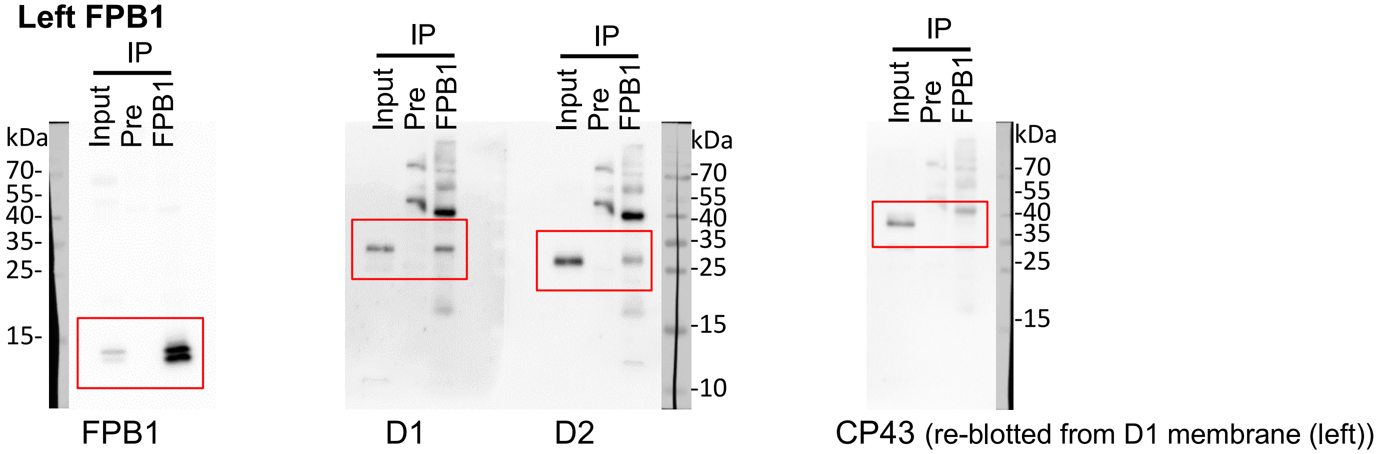

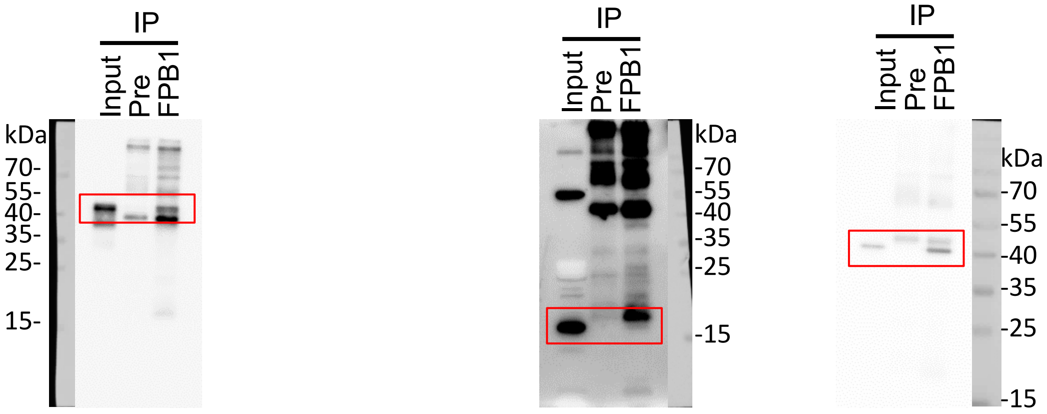

CP47 (re-blotted from D2 membrane (top)) PAM68 Alb3

Right PAM68

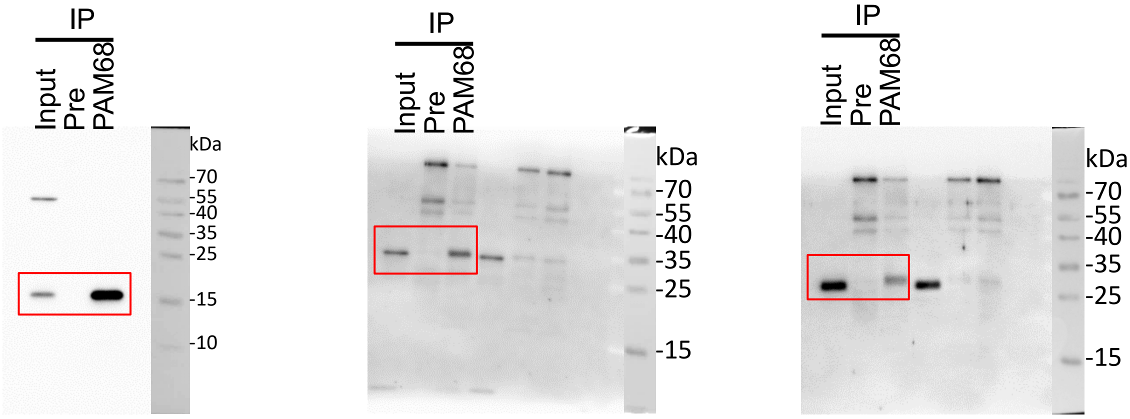

PAM68 D1 D2

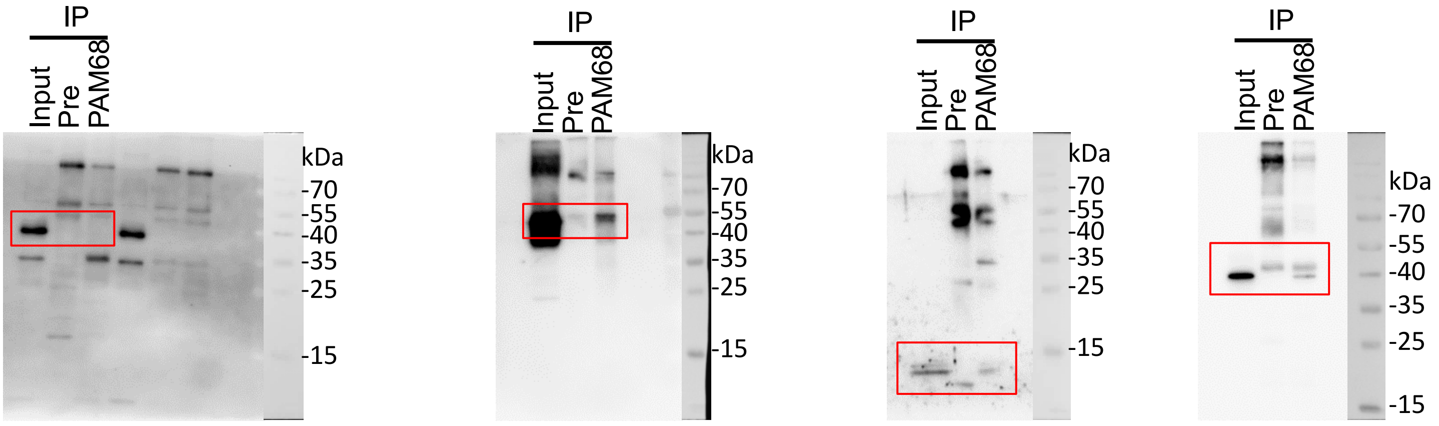

CP43 (re-blotted from D1 membrane (top)) CP47 FPB1 Alb3

Figure 5b

Left FPB1

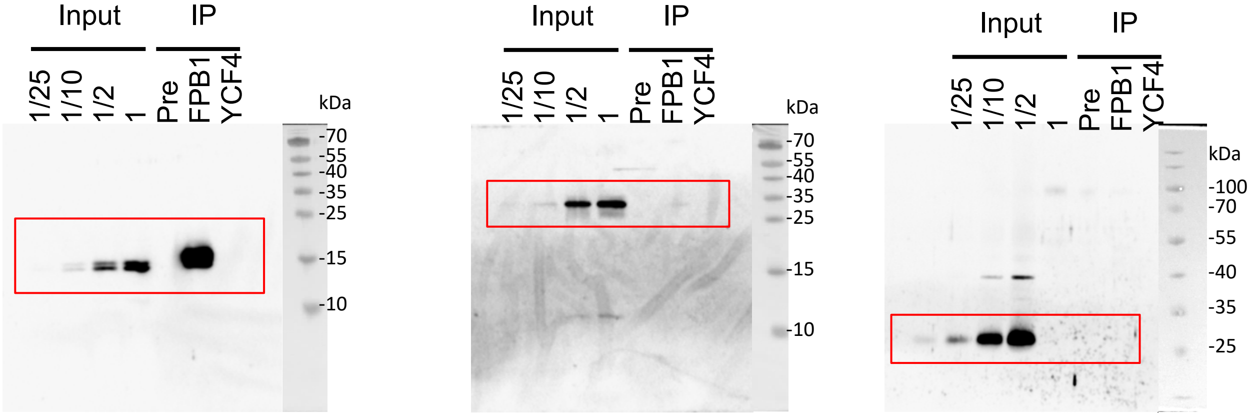

FPB1 D1 D2

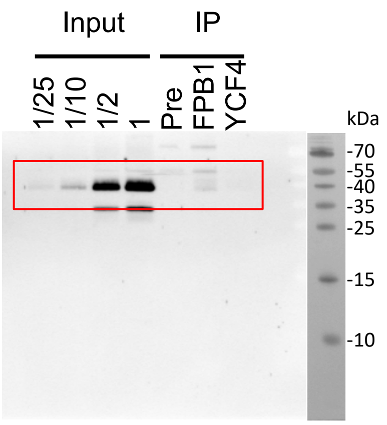

CP43 (re-blotted from D1 membrane (top))

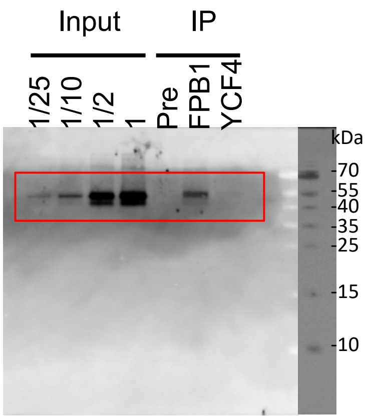

CP47

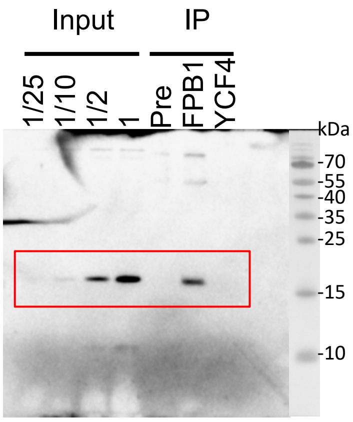

PAM68

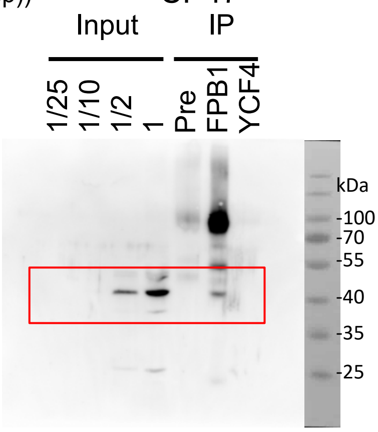

Alb3

Right PAM68

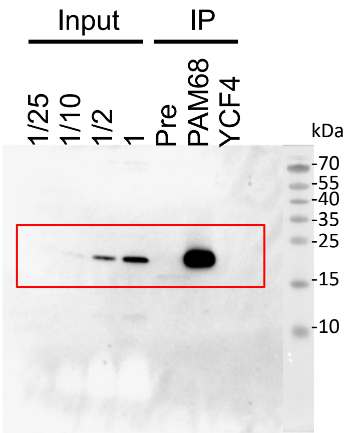

PAM68

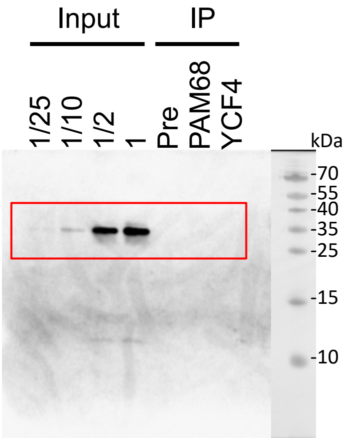

D1

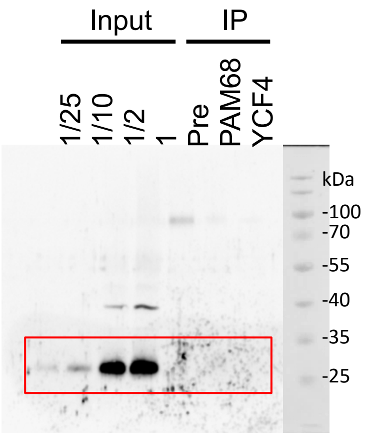

D2

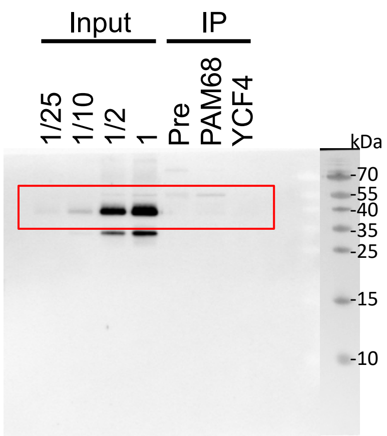

CP43 (re-blotted from D1 membrane (top))

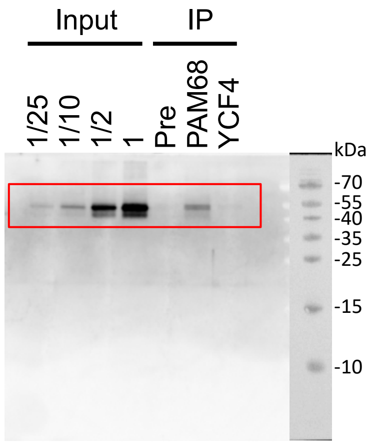

CP47

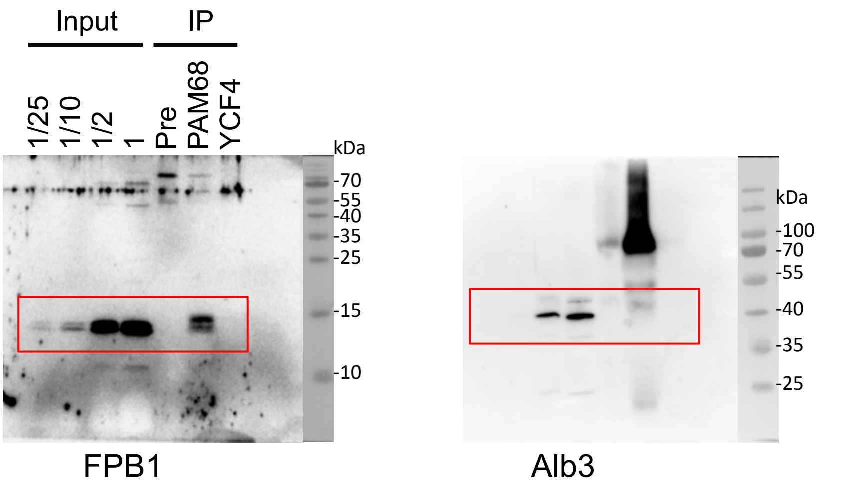
Input
IP
1/25
1/10
1/2
1
Pre
PAM68
YCF4
kDa
-70
-55
-40
-35
-25
-15
-10
FPB1
kDa
-100
-70
-55
-40
-35
-25
Alb3

Figure 5c

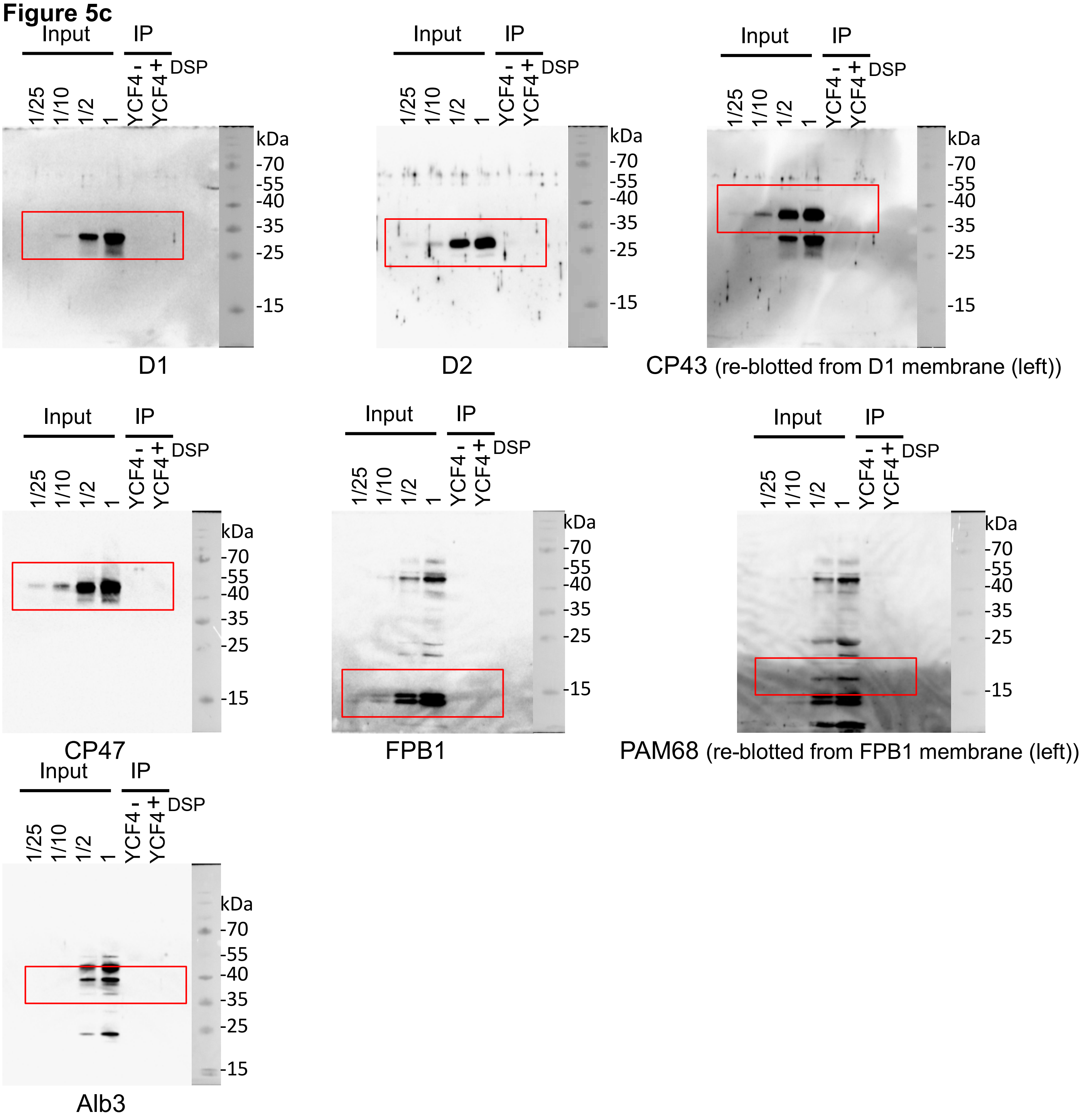
Input
IP
1/25
1/10
1/2
1
YCF4 -
YCF4 +
DSP
kDa
-70
-55
-40
-35
-25
-15
D1
D2
CP43 (re-blotted from D1 membrane (left))
CP47
FPB1
PAM68 (re-blotted from FPB1 membrane (left))
Alb3

Figure 5d

Cub-FPB1

$Alg5^{NubI}$ $Alg5^{NubG}$ $^{NubG}CP47$ $^{NubG}CP43$ $^{NubG}PsaA$ $^{NubG}PAM68$ $^{NubG}LPA1$ $^{NubG}LPA2$ $YCF4^{NubG}$

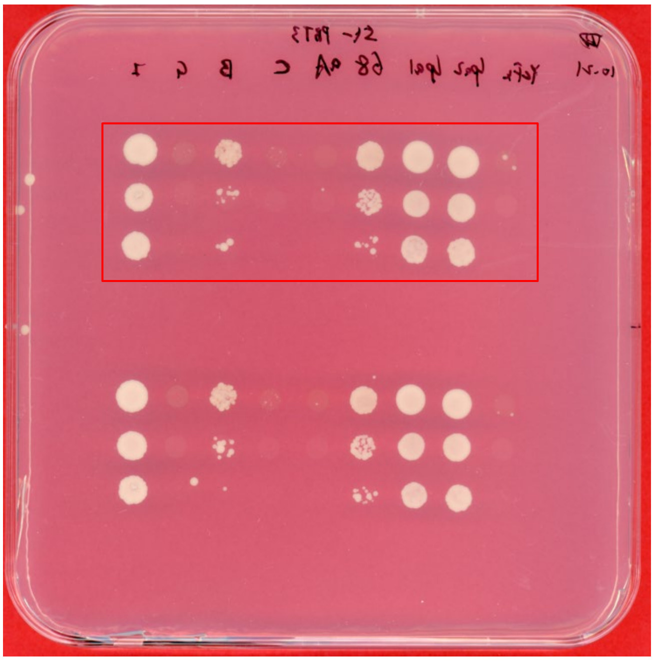

Alb3-Cub

$Alg5^{NubI}$ $Alg5^{NubG}$ $^{NubG}FPB1$ $^{NubG}PAM68$ $YCF4^{NubG}$

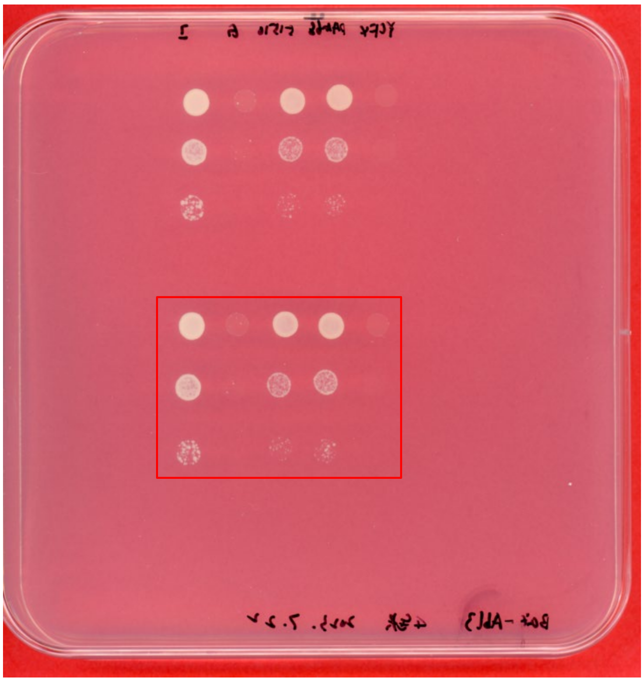

SecY1-Cub

$Alg5^{NubI}$ $Alg5^{NubG}$ $^{NubG}FPB1$ $^{NubG}PAM68$ $YCF4^{NubG}$

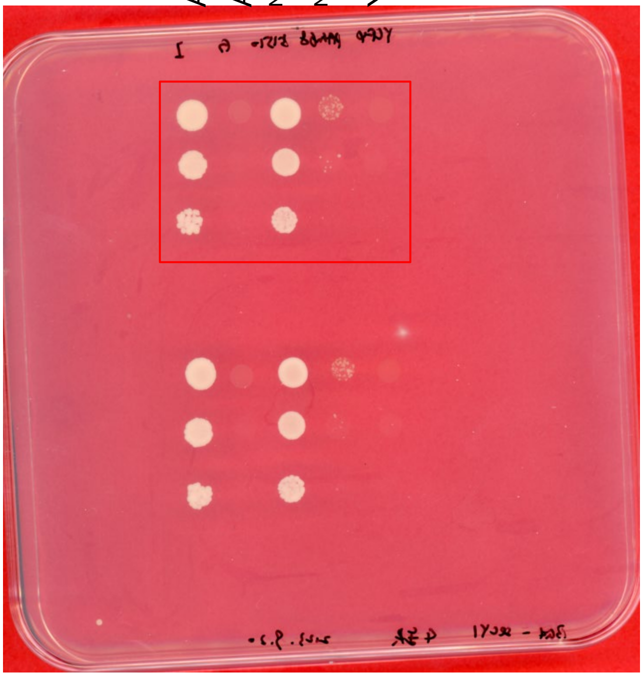

Figure S2

Figure S2a

Sequencing with SALK-LBa1 primer:

AGATTCGCTTTGAGTTGGAGTCCACGTTCTTTAATAGTGGACTCTTGTTCCAAACTGGAACAACAC
TCAACCCTATCTCGGGCTATTCTTTTGATTTATAAGGGATTTTGCCGATTTCGGAACCACCATCAAA
CAGGATTTTCGCCTGCTGGGGCAAACCAGCGTGGACCGCTTGCTGCAACTCTCTCAGGGCCAG
GCGGTGAAGGGCAATCAGCTGTTGCCCGTCTCACTGGTGAAAAGAAAAACCACCCCAGTACATT
AAAAACGTCCGCAATGTGTTATTAAGTTGTCTAAGCGTCAATTTGTTTACACCACAATATAGCTGTT
GCGTCATTATTAAGTTTAA//ACCTCCTTGGGCTAGAAACGAAGGTCGTTCTTCTACGTCCCAAGAG
AGCTTTGAGGTTCCTTTCTTTGTTTATCTGCTAGCTTCCGCGATTACTGCCATTGCTGCTGTATGC
TTCTTCTTCTCTCTCTTTCACTCCTTTTGTTGTTTGCATTGCTGATTCTCATATCAAAGATGTTATCT
TAATTATGCATCGTTTGGTCACTGTAGATTGGTTCTGTTTTCGAGTACACAAGCAAGAATCCAGTTT
TCGGGATCTTGGAATCTGACAGCATCTTTTATACTCCTGTGCTTGGATTCTTTGCTCTTACTGGAAT
CCCCCACTTCTGA

Blast:

>AT3G51510.1 | Symbols: no symbol available | no full name available | chr3:19108990-19110372

FORWARD LENGTH=1383

Length=1383

Score = 581 bits (643), Expect = 2e-164

Identities = 325/326 (99%), Gaps = 1/326 (0%)

Strand=Plus/Plus

```
Query  347  ACCTCCTTGGGCTAGAAACGAAGGTCGTTCTTCTACGTCCCAAGAGAGCTTTGAGGTTCC  406
            ||||||||||||||||||||||||||||||||||||||||||||||||||||||||||||
Sbjct  374  ACCTCCTTGGGCTAGAAACGAAGGTCGTTCTTCTACGTCCCAAGAGAGCTTTGAGGTTCC  433

Query  407  TTTCTTTGTTTATCTGCTAGCTTCCGCGATTACTGCCATTGCTGCTGTATGCTTCTTCTT  466
            ||||||||||||||||||||||||||||||||||||||||||||||||||||||||||||
Sbjct  434  TTTCTTTGTTTATCTGCTAGCTTCCGCGATTACTGCCATTGCTGCTGTATGCTTCTTCTT  493

Query  467  CTCTCTCTTTCACTCCTTTTGTTGTTTGCATTGCTGATTCTCATATCAAAGATGTTATCT  526
            ||||||||||||||||||||||||||||||||||||||||||||||||||||||||||||
Sbjct  494  CTCTCTCTTTCACTCCTTTTGTTGTTTGCATTGCTGATTCTCATATCAAAGATGTTATCT  553

Query  527  TAATTATGCATCGTTTGGTCACTGTAGATTGGTTCTGTTTTCGAGTACACAAGCAAGAAT  586
            ||||||||||||||||||||||||||||||||||||||||||||||||||||||||||||
Sbjct  554  TAATTATGCATCGTTTGGTCACTGTAGATTGGTTCTGTTTTCGAGTACACAAGCAAGAAT  613

Query  587  CCAGTTTTCGGGATCTTGGAATCTGACAGCATCTTTTATACTCCTGTGCTTGGATTCTTT  646
            ||||||||||||||||||||||||||||||||||||||||||||||||||||||||||||
Sbjct  614  CCAGTTTTCGGGATCTTGGAATCTGACAGCATCTTTTATACTCCTGTGCTTGGATTCTTT  673

Query  647  GCTCTTACTGGAATCCCCCACTTCTG  672
            ||||||||||||| ||||||||||||
Sbjct  674  GCTCTTACTGGAAT-CCCCACTTCTG  698
```

Figure S2b

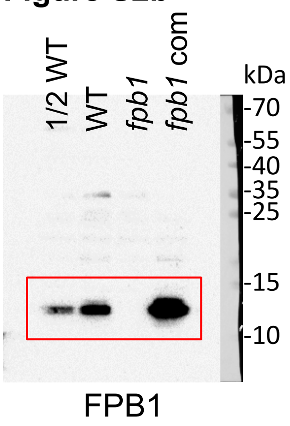

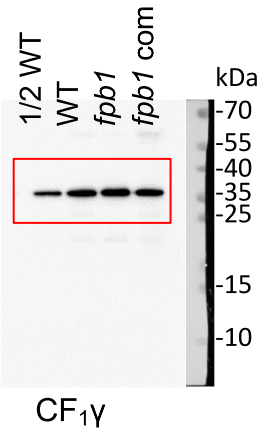

Figure S4

Figure S4a

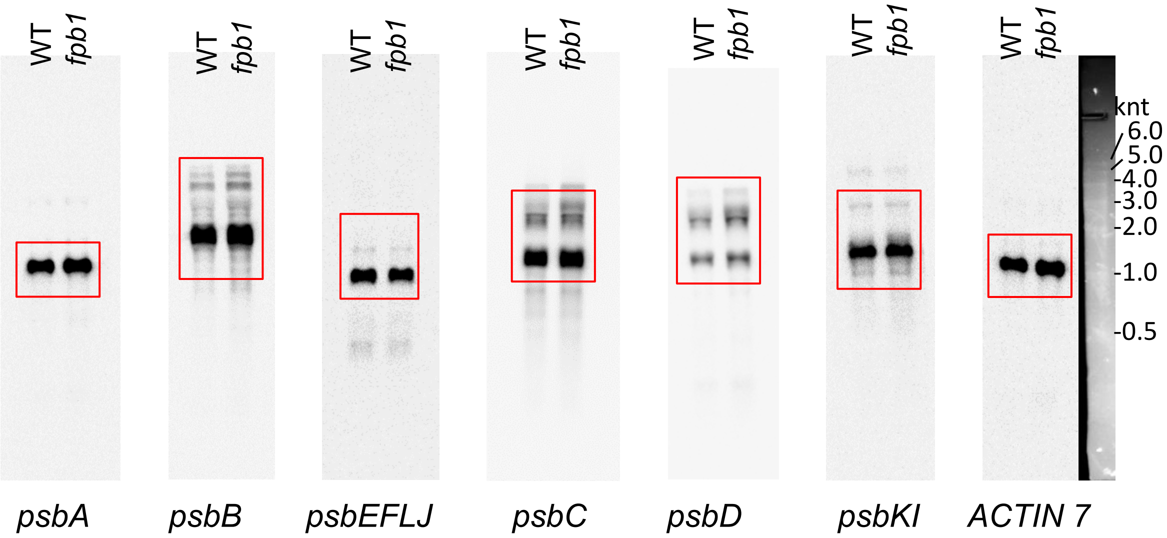

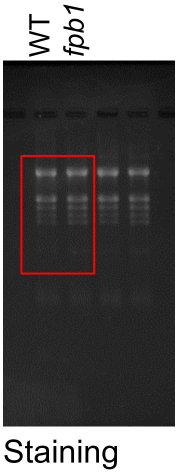

Figure S4b

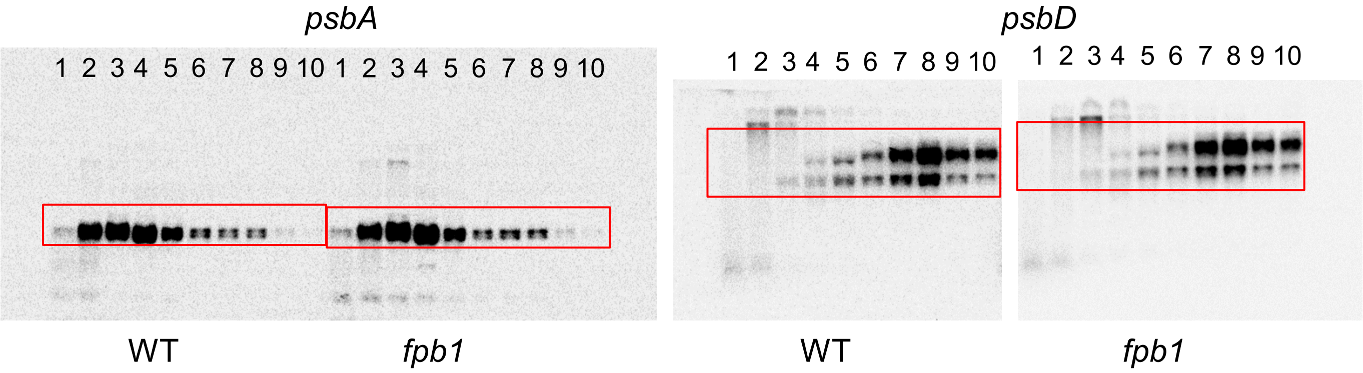

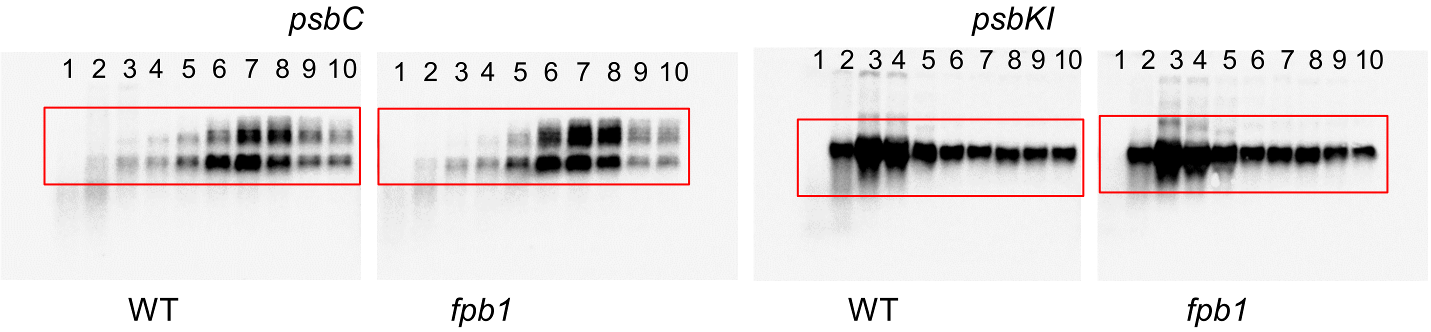

Staining

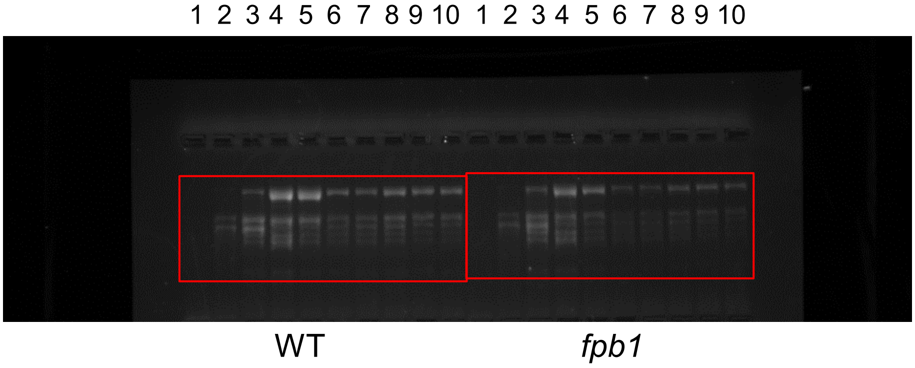

Figure S5

Figure S5a

FPB1

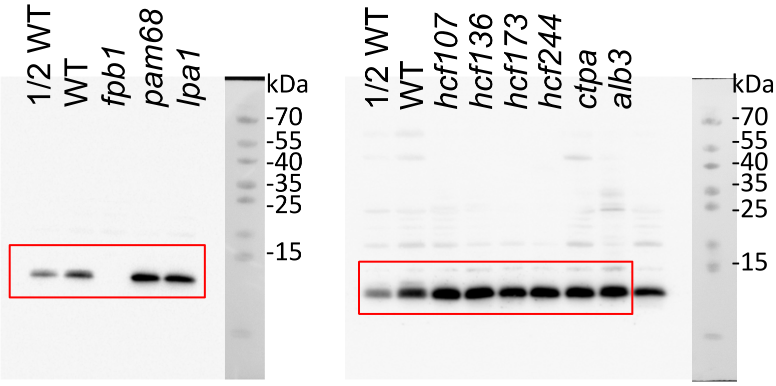

PAM68

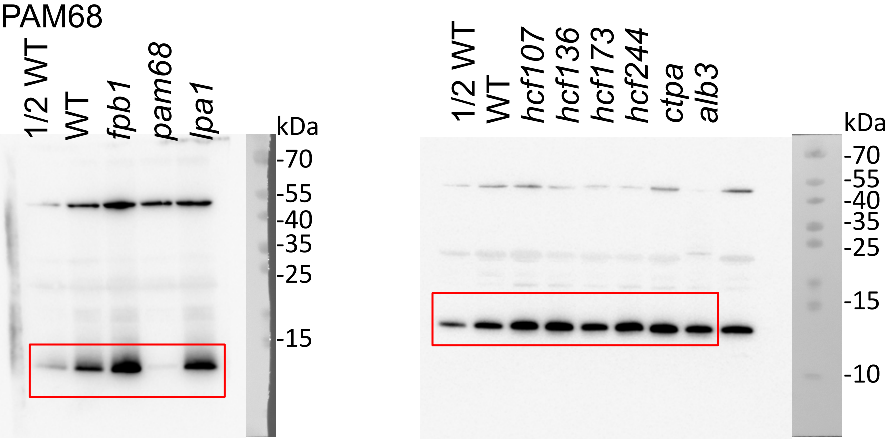

LPA1

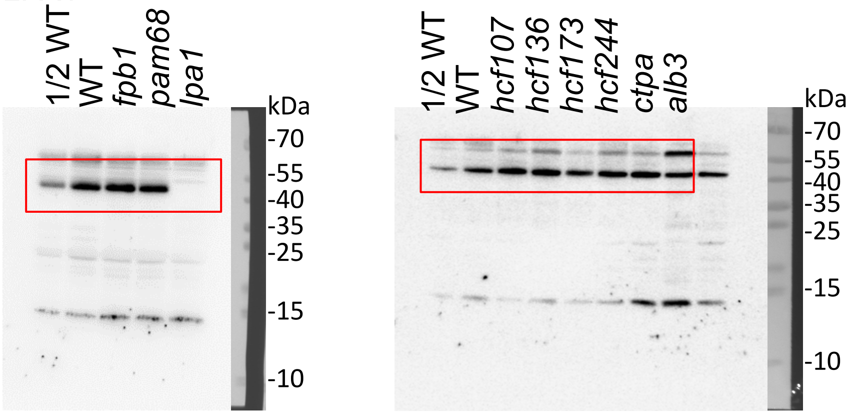

HCF136

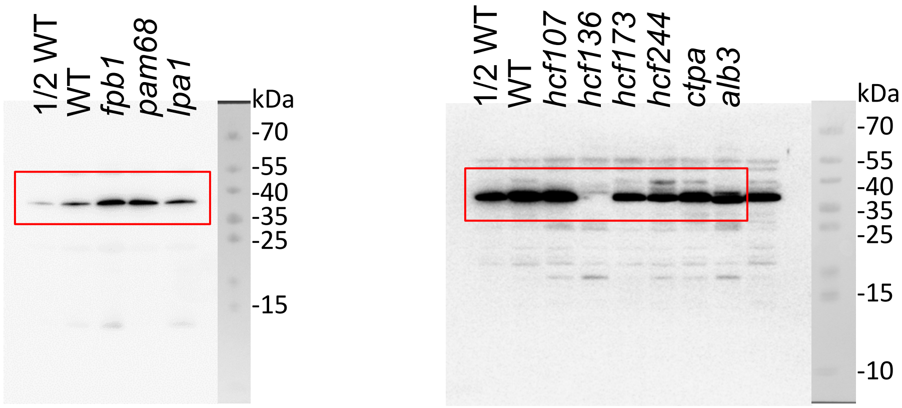

HCF244

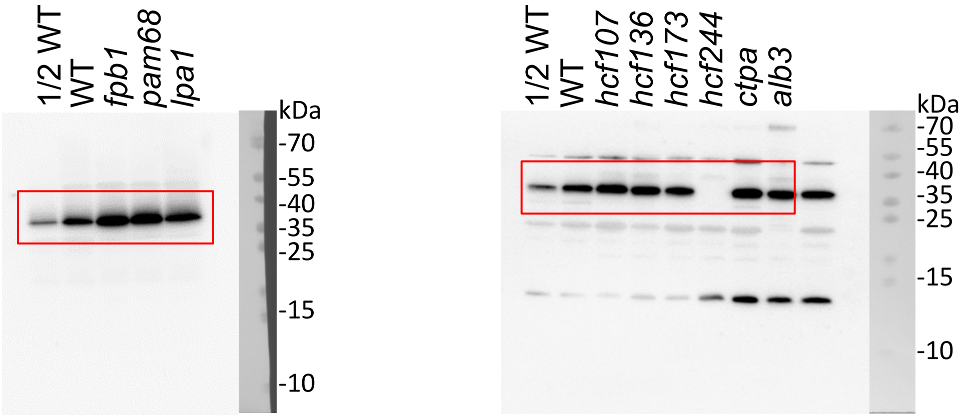

Figure S5b

FPB1

WT

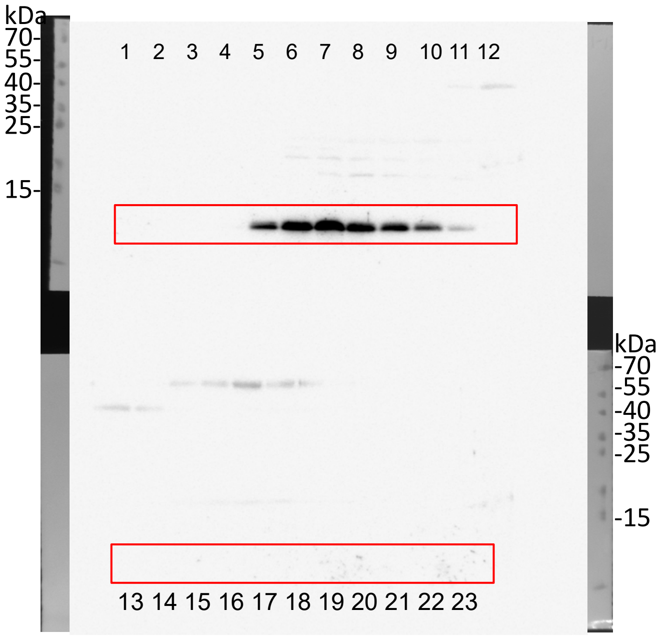

pam68

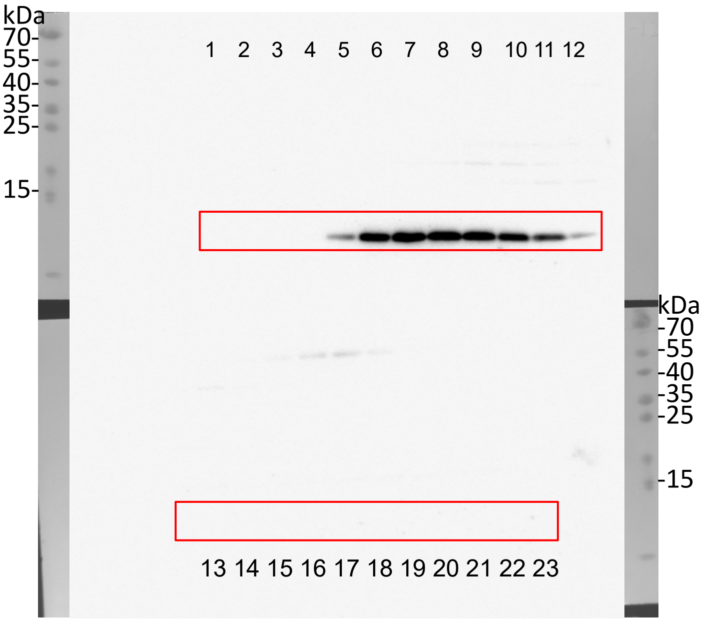

PAM68

WT

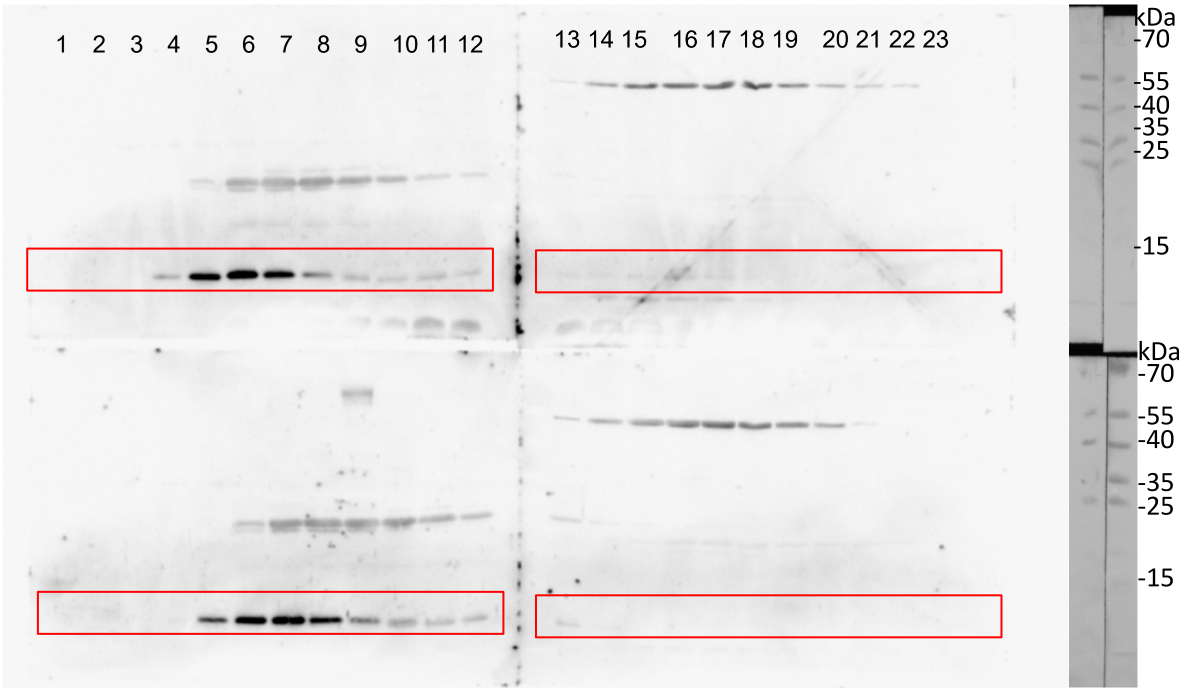

fpb1

WT

Alb3

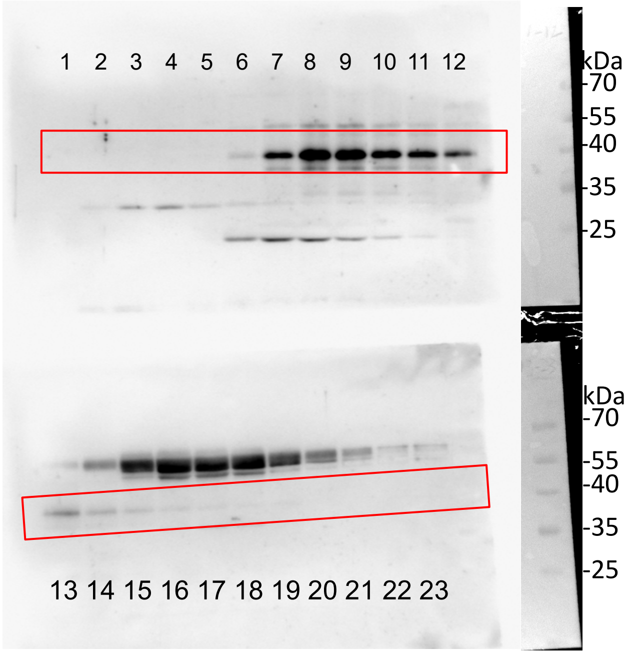

D1

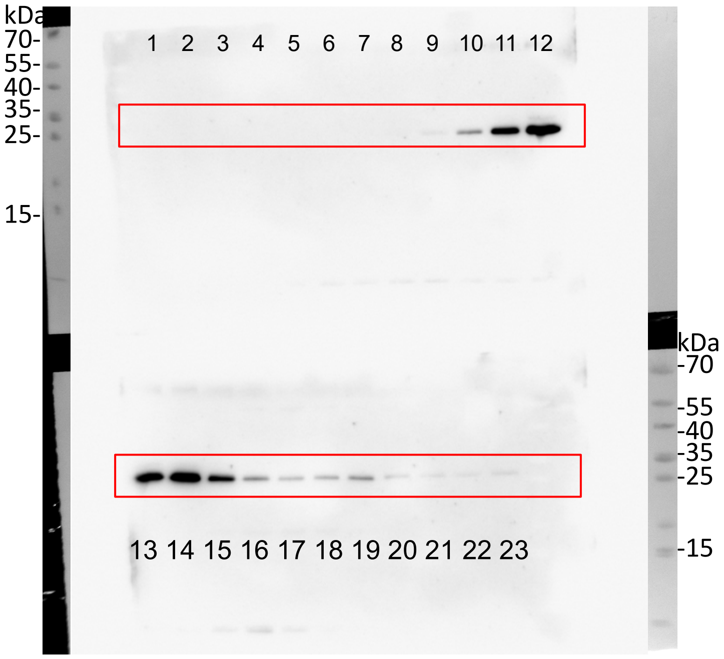

D2

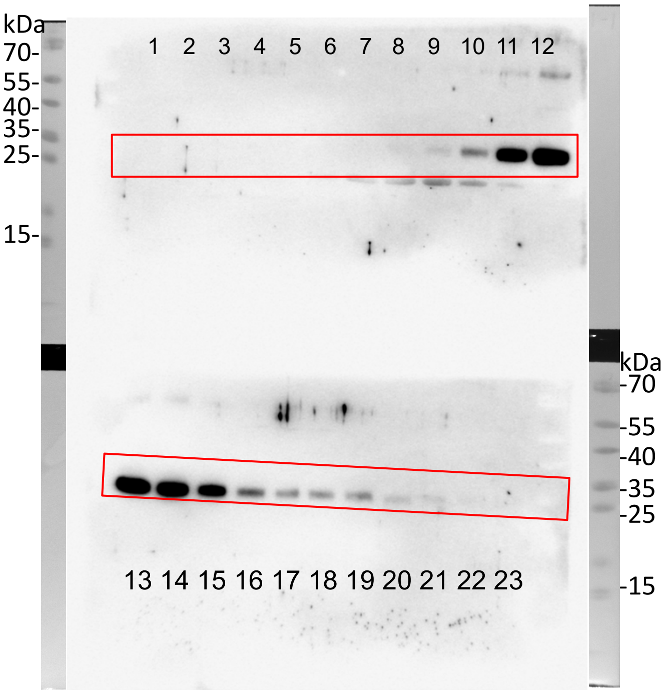

CP43 (re-blotted from D1 membrane (top))

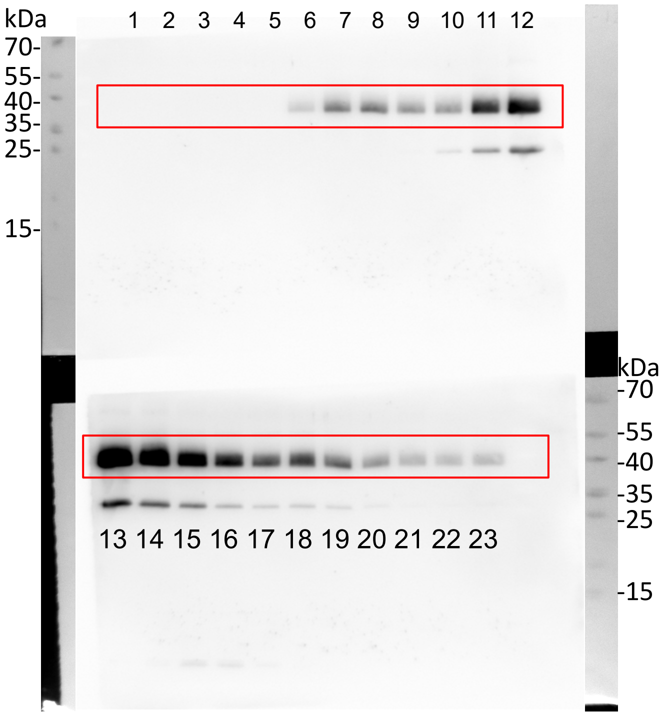

CP47 (re-blotted from D2 membrane (top))

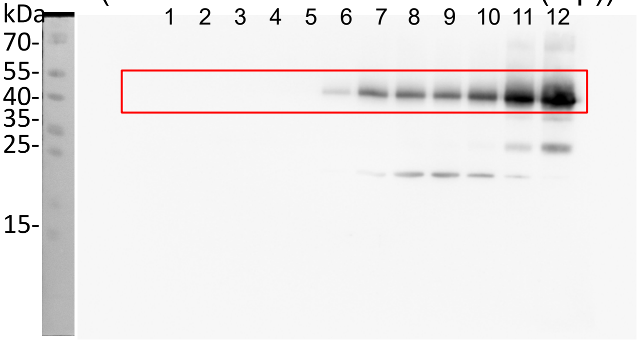

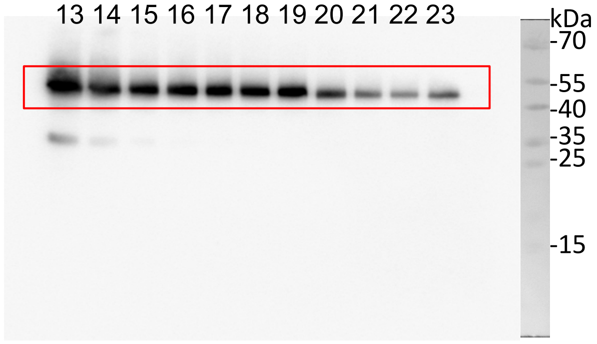

PsaD (re-blotted from CP43 membrane (top))

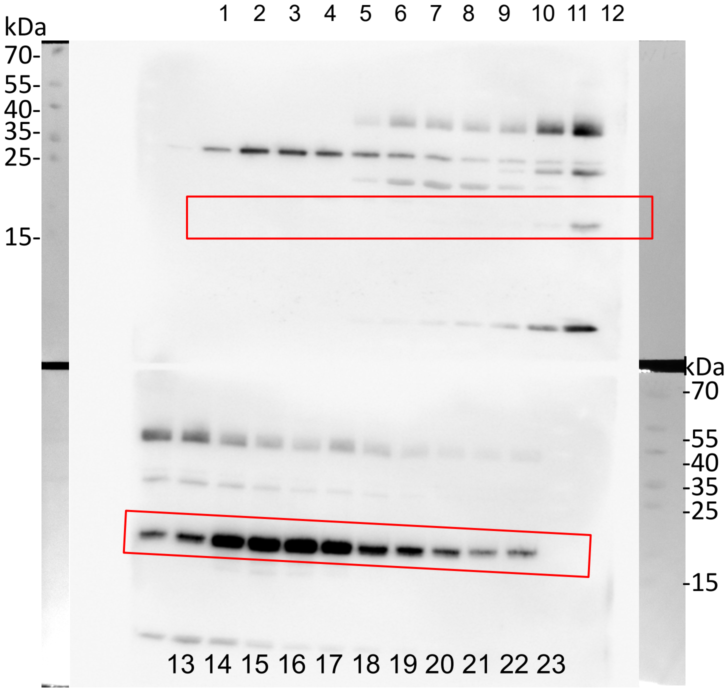

LHCII

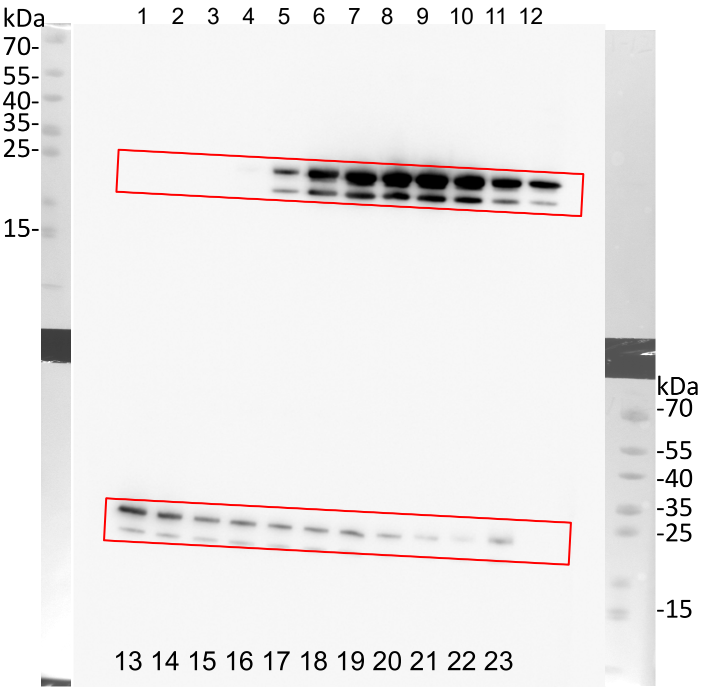

Figure S6a

Left

YFP Chl. Merged

FPB1-YFP^N+PAM68-YFP^C

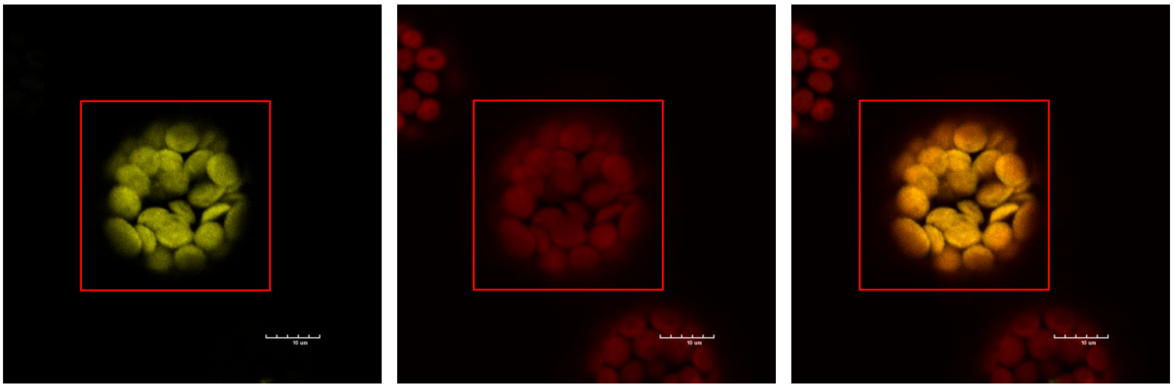

FPB1-YFP^N+LPA1-YFP^C

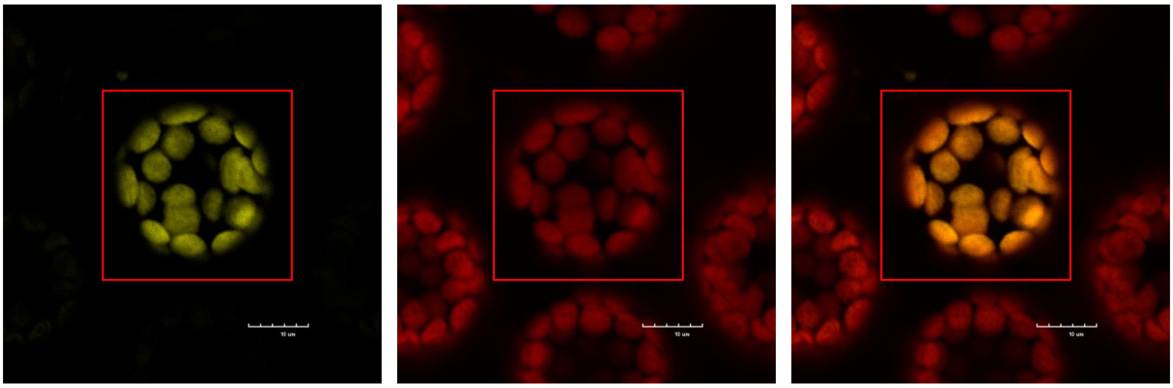

FPB1-YFP^{N}+LPA2-YFP^{C}

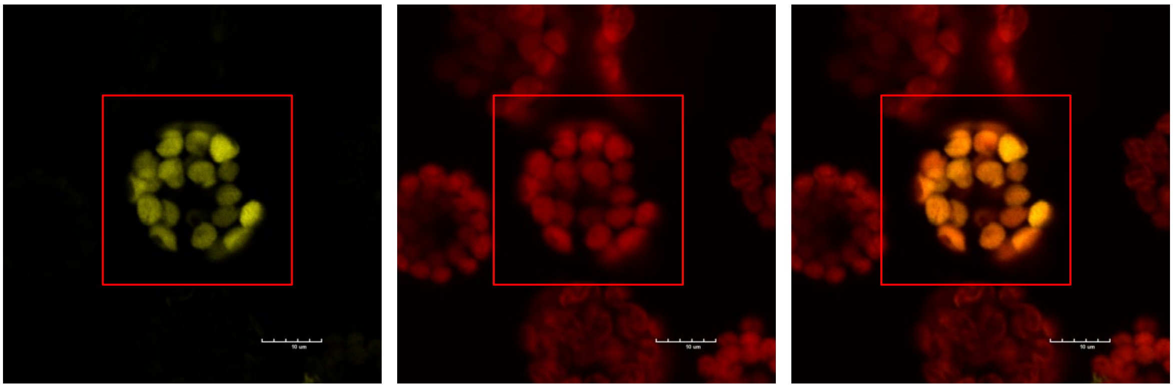

FPB1-YFP^{N}+YFP^{C}-SecY1

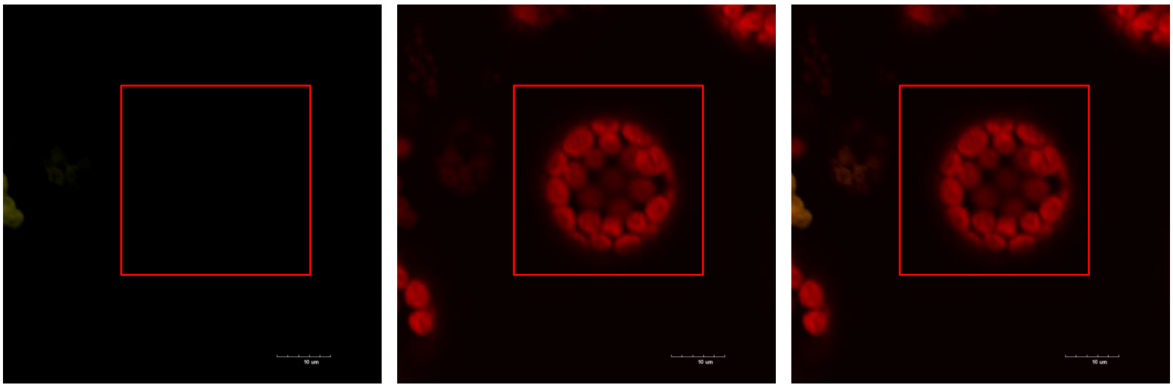

FPB1-YFP^{N}+YFP^{C}-SecE1

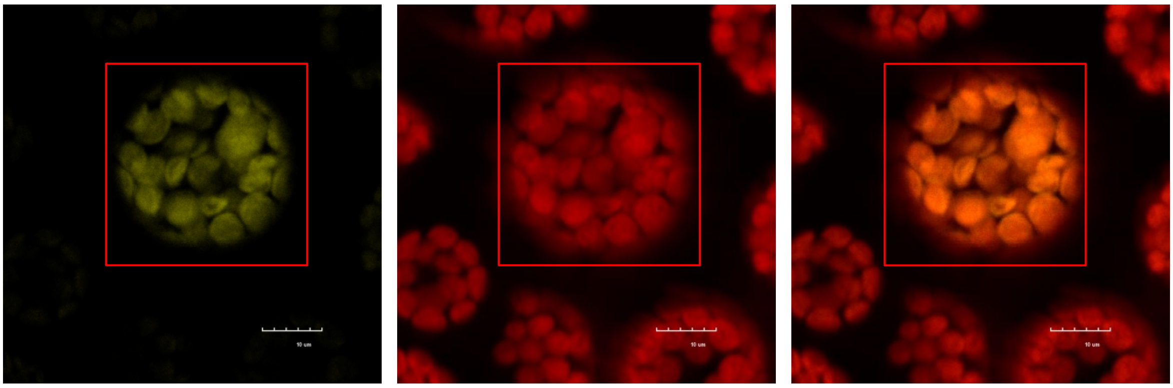

FPB1-YFP^{N}+FtsY-YFP^{C}

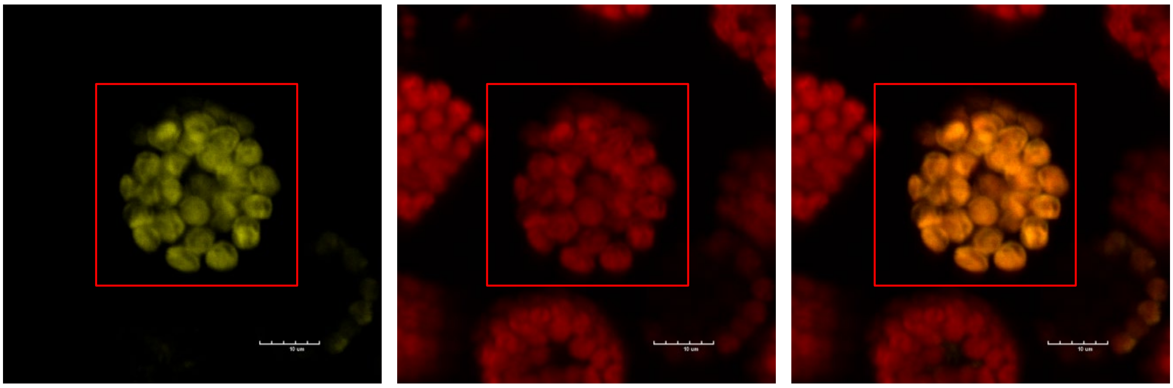

FPB1-YFP^{N}+ cpSRP54-YFP^{C}

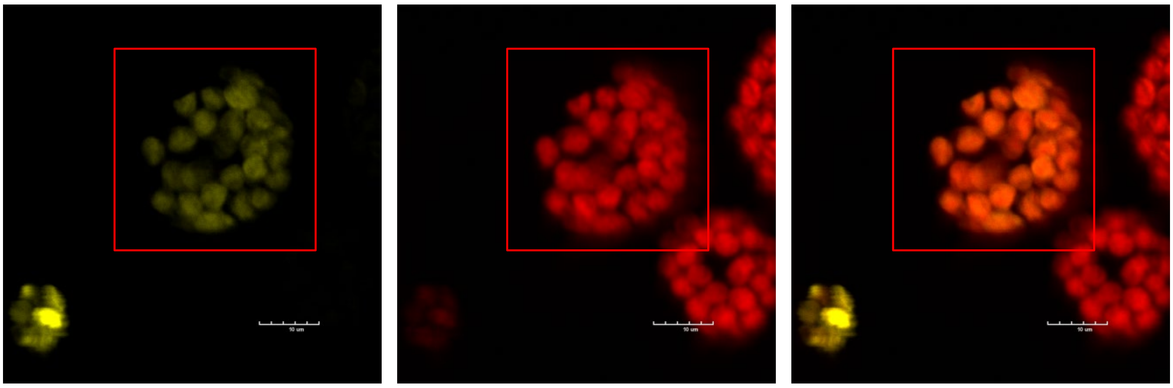

FPB1-YFP^{N}+Alb3-YFP^{C}

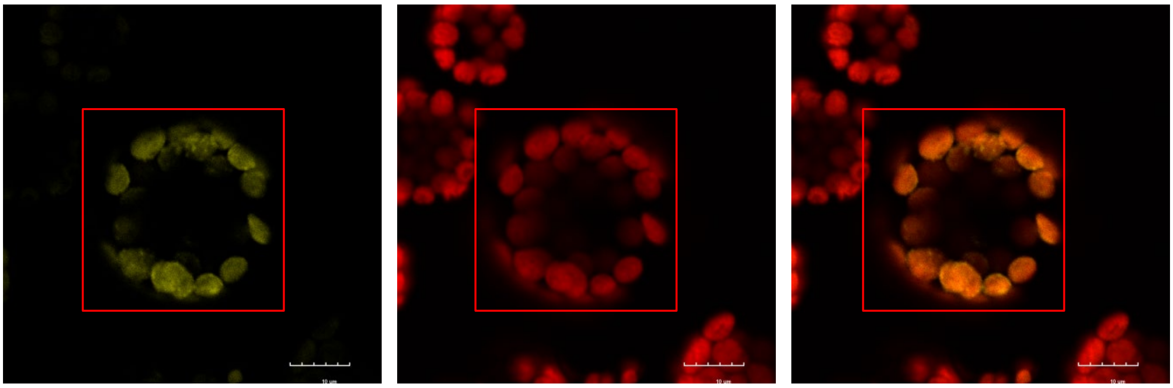

FPB1-YFP^{N}+SecA1-YFP^{C}

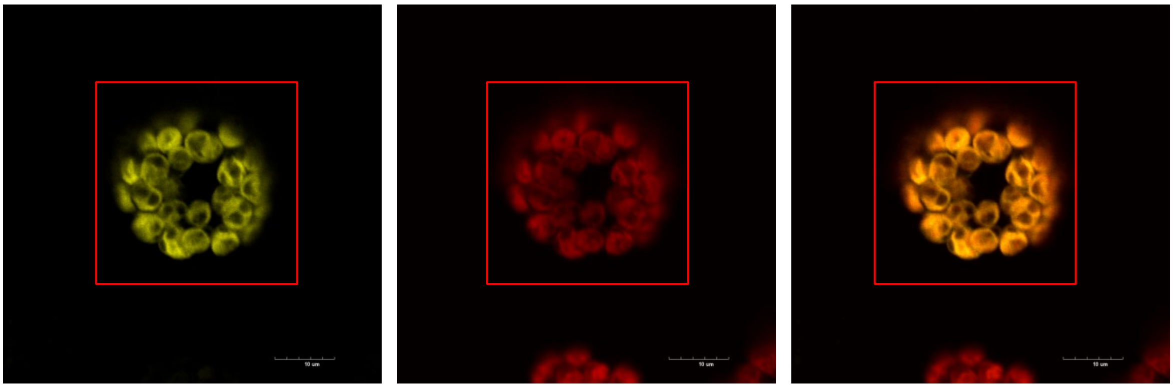

FPB1-YFP^{N}+YCF4-YFP^{C}

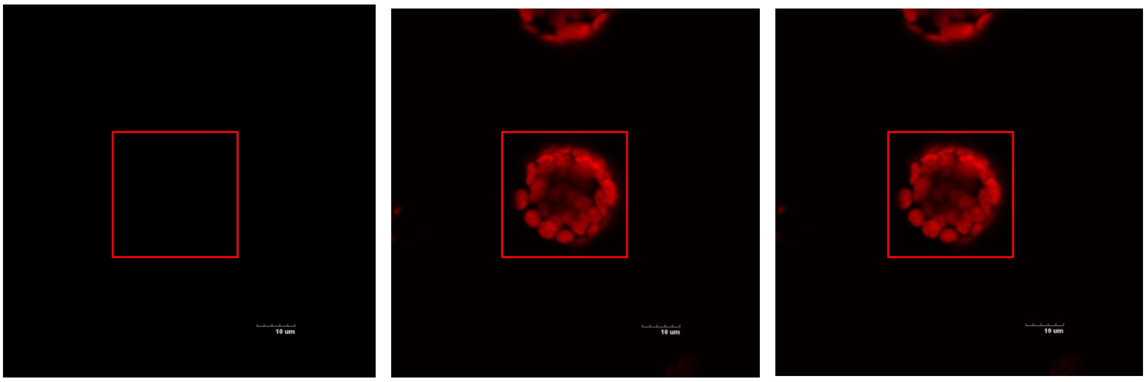

Figure S6a
Right

YFP Chl. Merged

PAM68-YFP^{N}+ FPB1-YFP^{C}

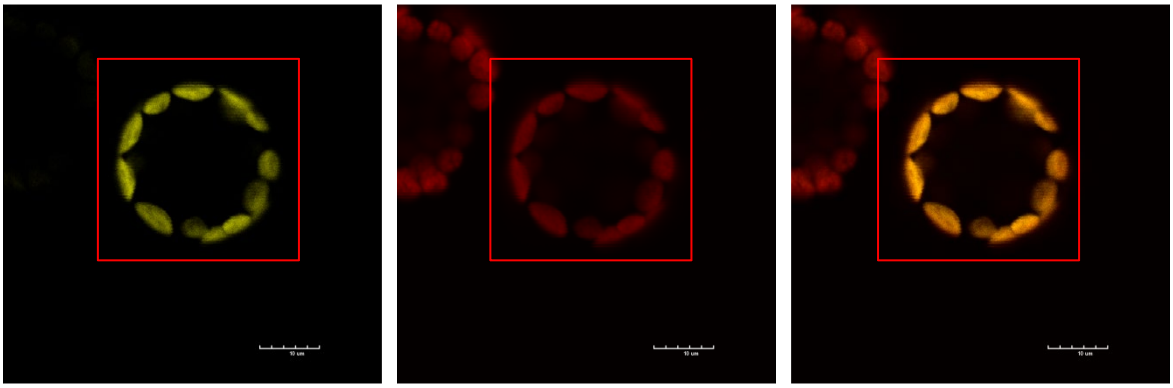

PAM68-YFP^{N}+LPA1-YFP^{C}

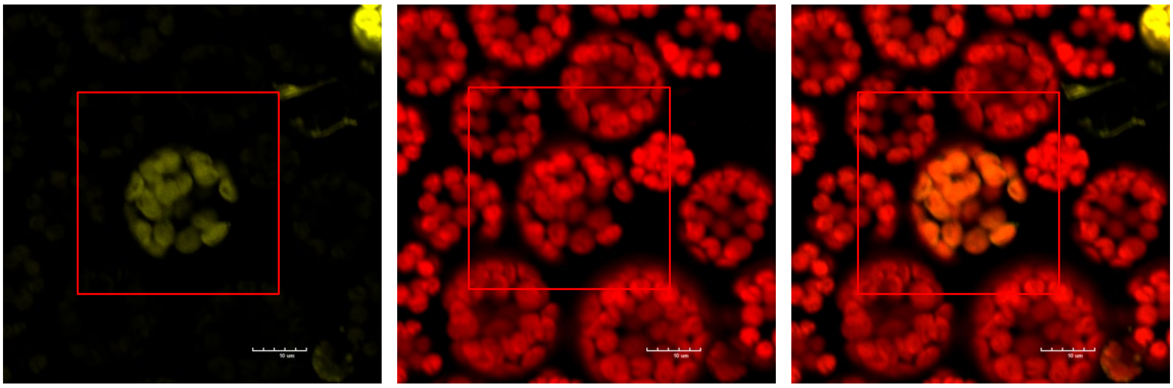

PAM68-YFP^{N}+LPA2-YFP^{C}

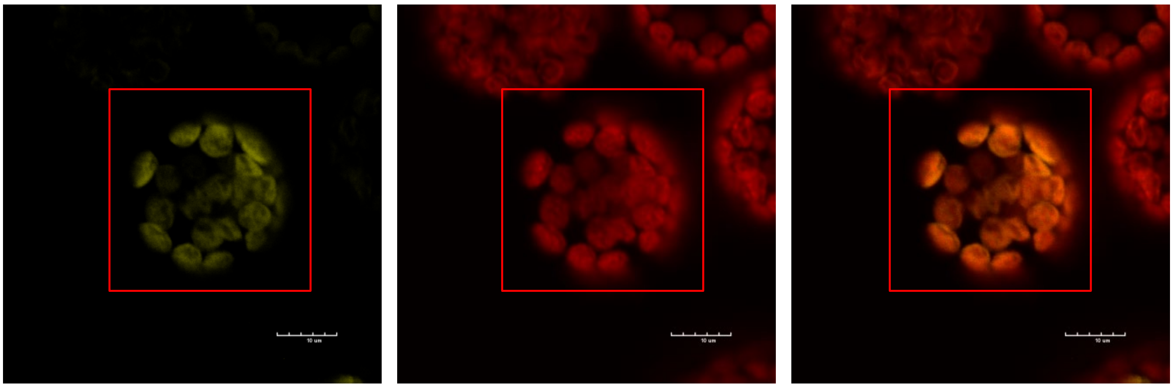

PAM68-YFP^{N}+YFP^{C}-SecY1

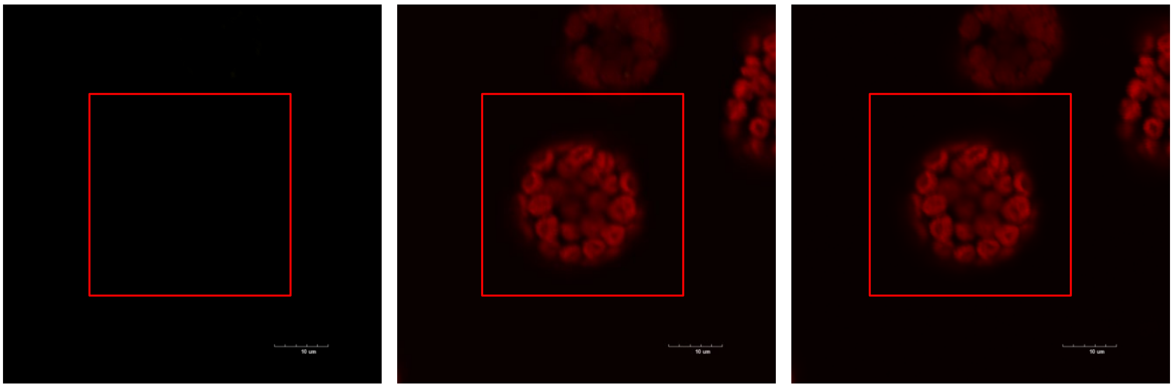

PAM68-YFP^{N}+YFP^{C}-SecE1

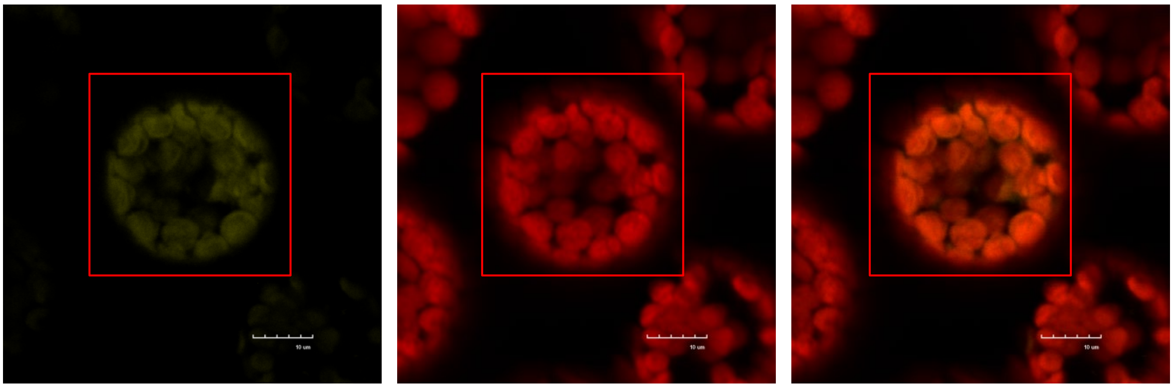

PAM68-YFP^{N}+FtsY-YFP^{C}

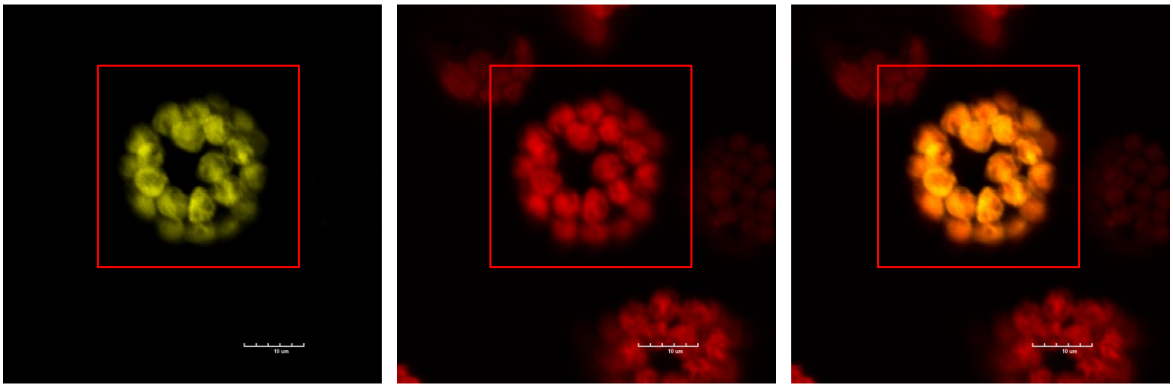

PAM68-YFP^{N}+ cpSRP54-YFP^{C}

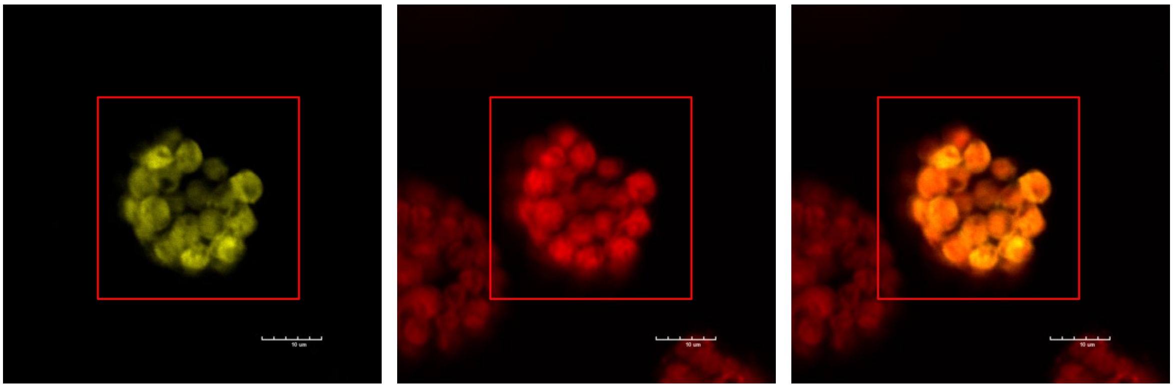

PAM68-YFP^{N}+Alb3-YFP^{C}

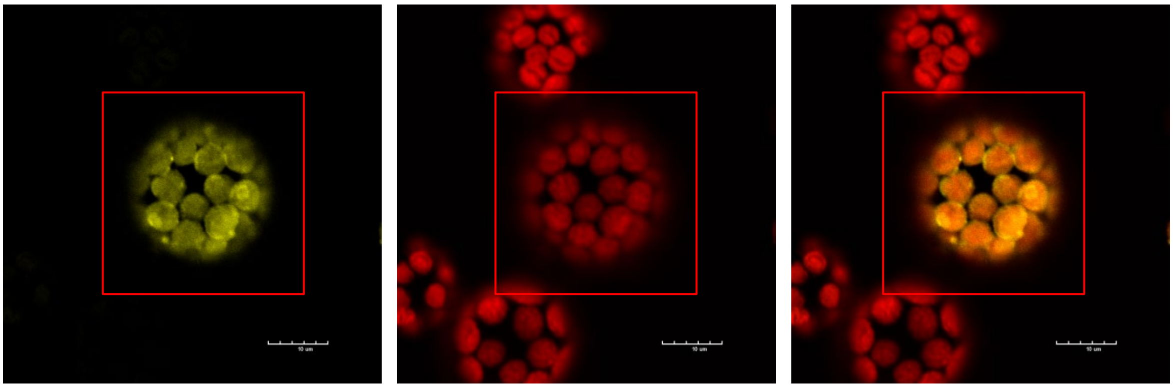

PAM68-YFP^{N}+SecA1-YFP^{C}

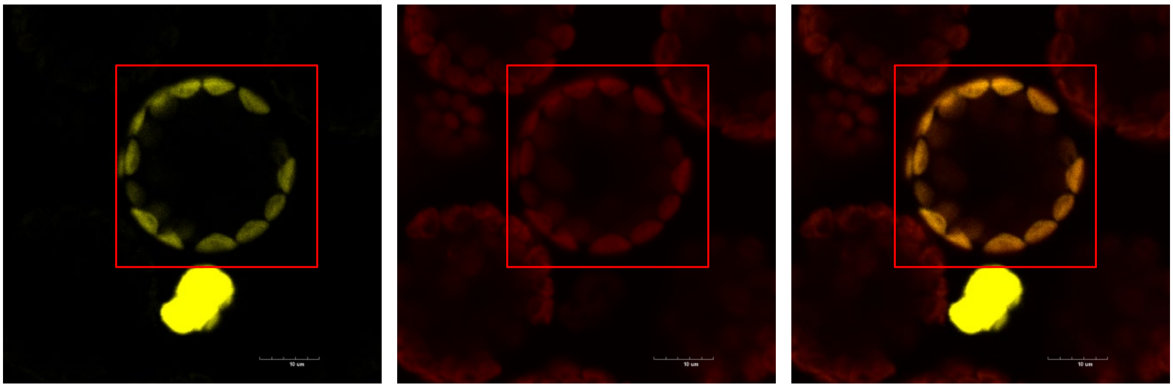

PAM68-YFP^{N}+YCF4-YFP^{C}

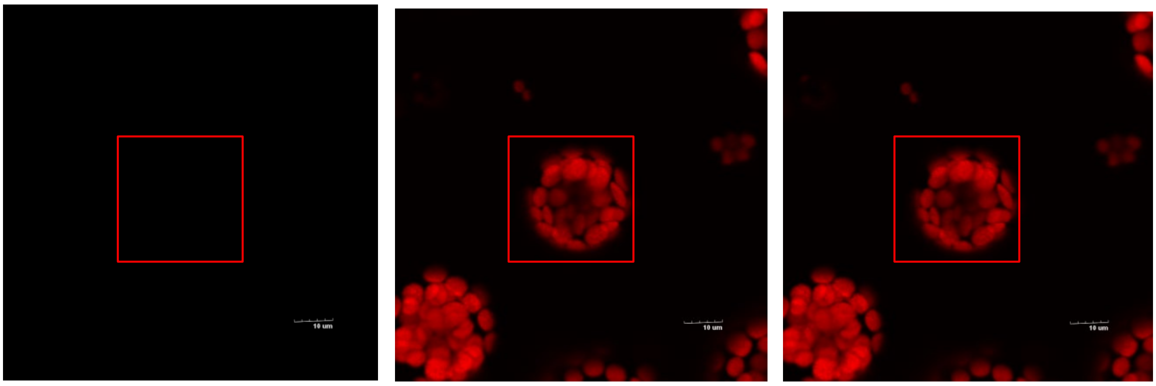

Figure S6b

Left

YFP Chl. Merged

YFP^{N}+FPB1-YFP^{C}

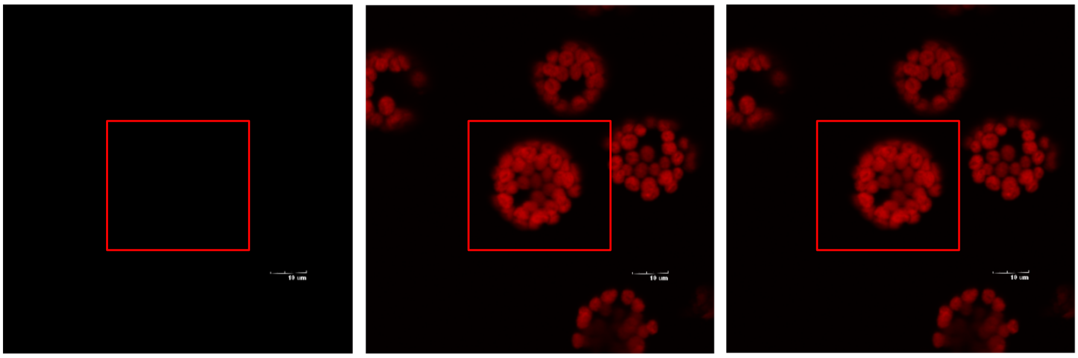

YFP^{N}+PAM68-YFP^{C}

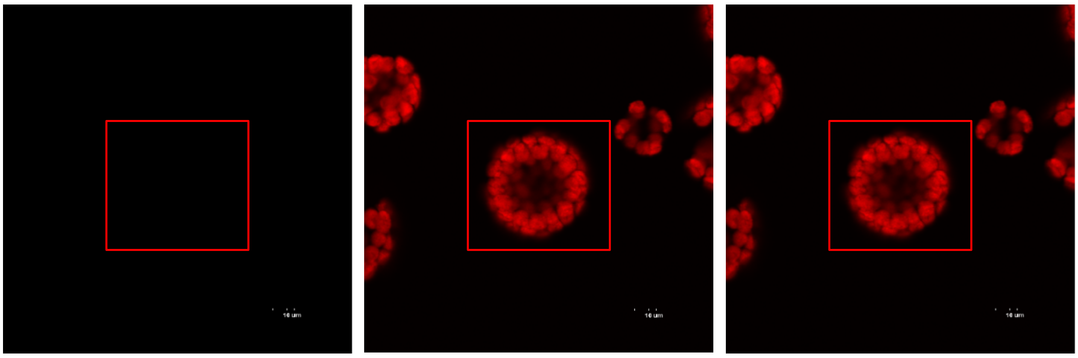

YFP^N +LPA1-YFP^C

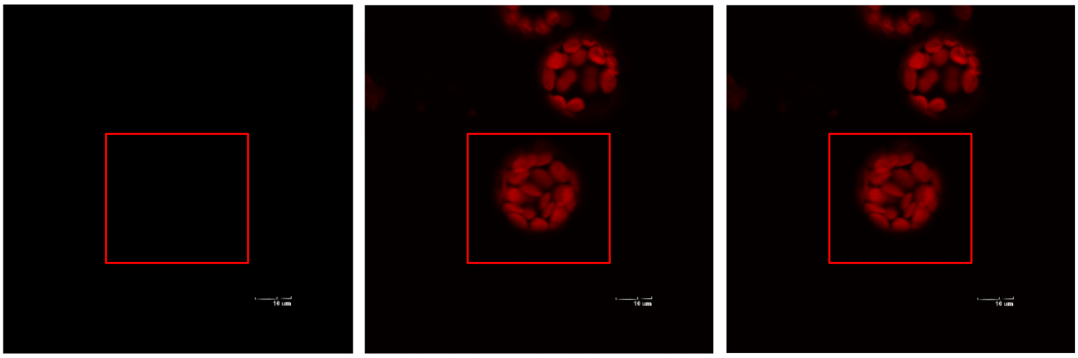

YFP^N +LPA2-YFP^C

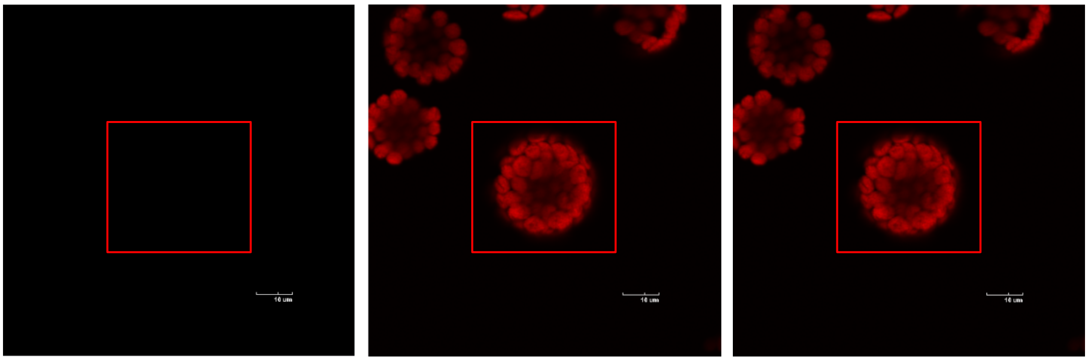

YFP^N +YFP^C-SecY1

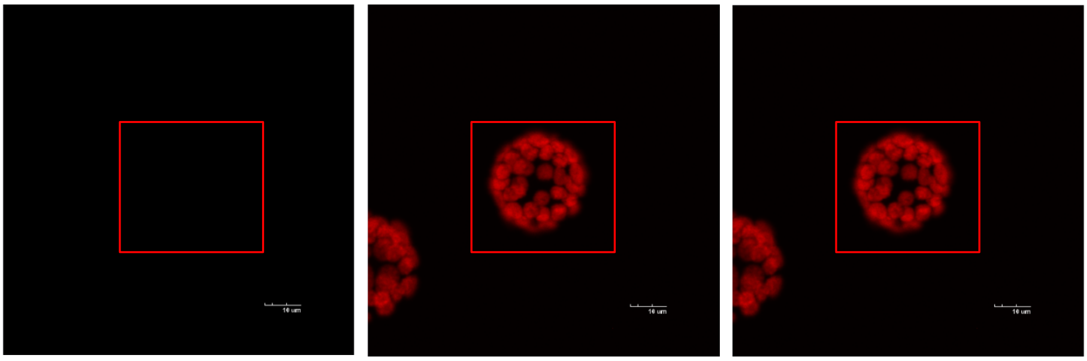

YFP^N +YFP^C-SecE1

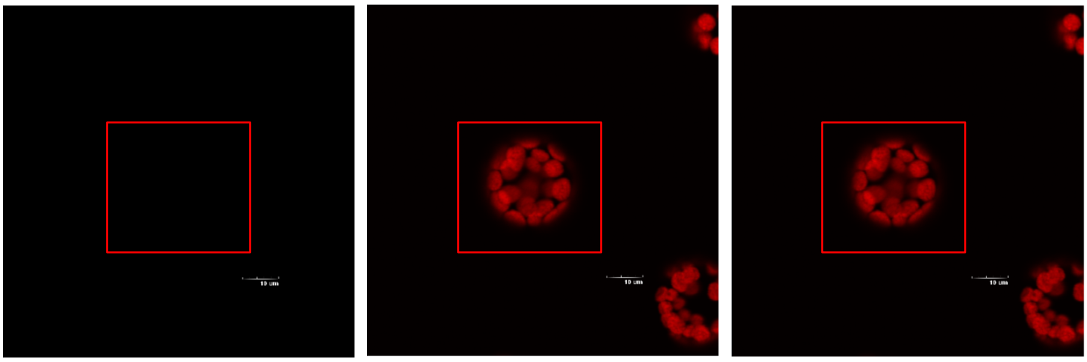

YFP^{N} +FtsY-YFP^{C}

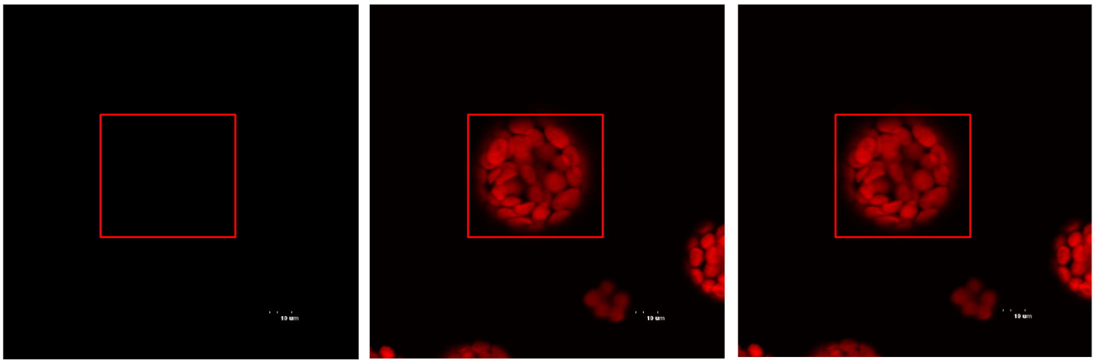

YFP^{N} +cpSPR54-YFP^{C}

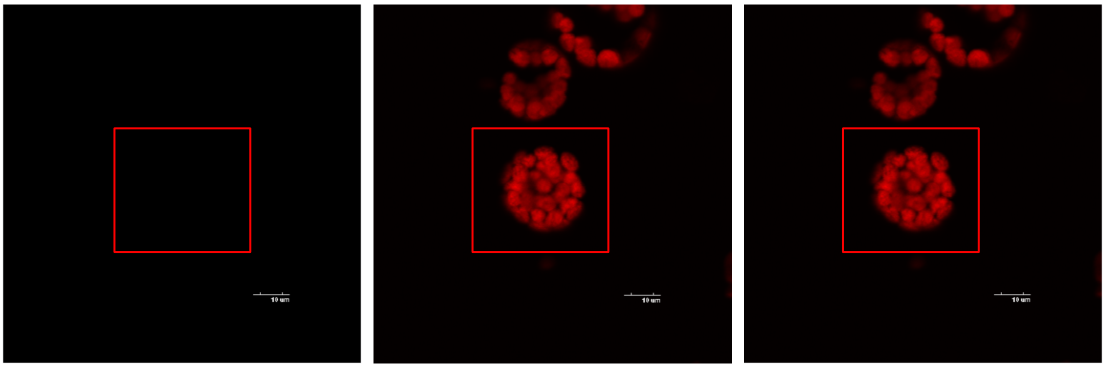

YFP^{N} +Alb3-YFP^{C}

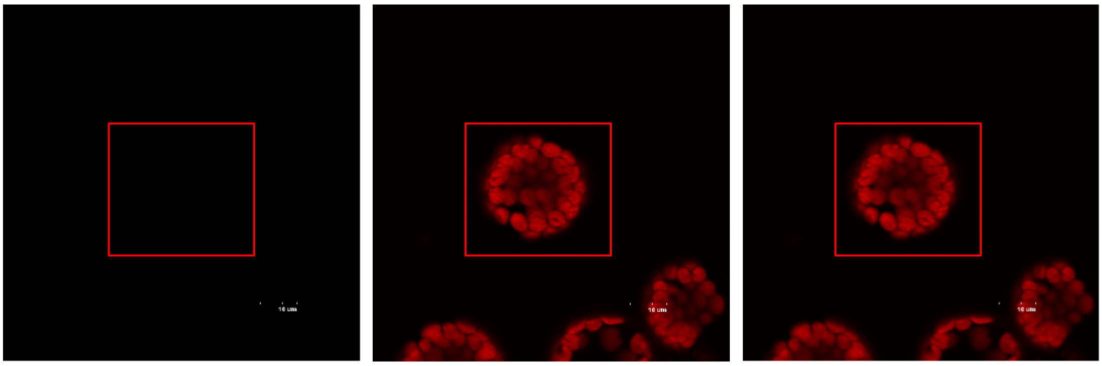

YFP^{N} +SecA1-YFP^{C}

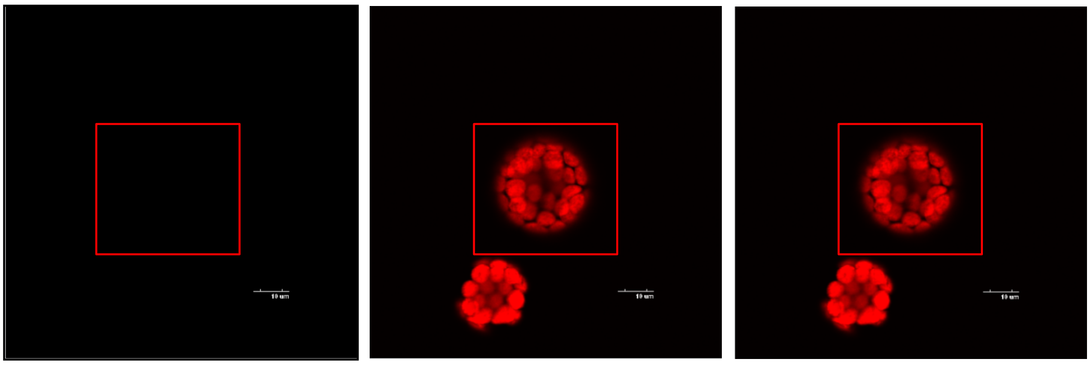

YFP^{N}+YCF4-YFP^{C}

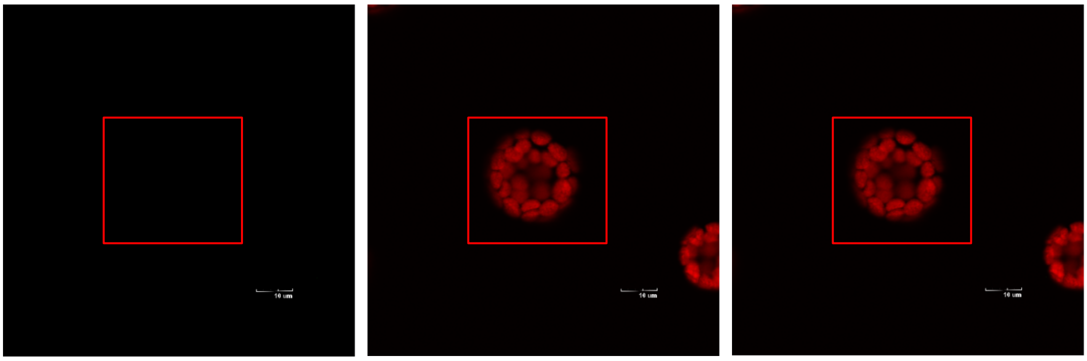

Figure S6b
right

YFP Chl. Merged

YCF4-YFP^{N}/FPB1-YFP^{C}

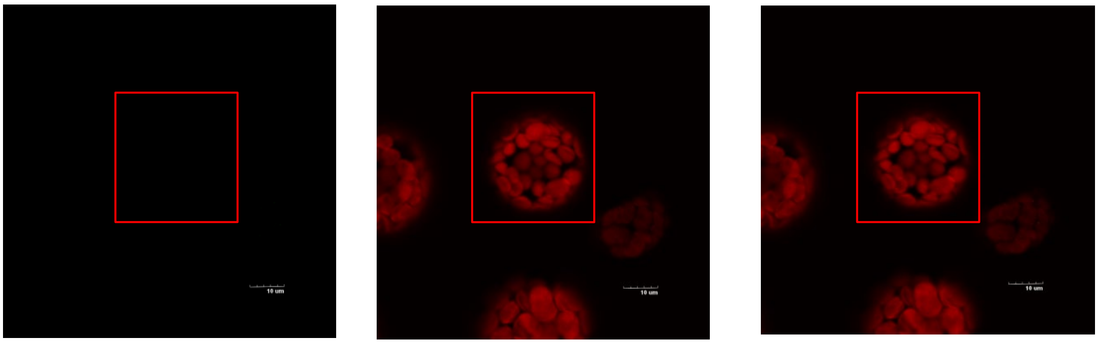

YCF4-YFP^{N}/PAM68-YFP^{C}

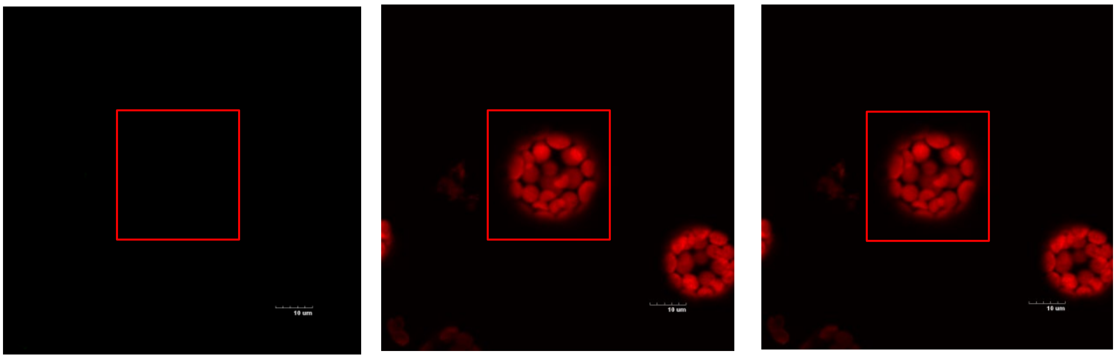

YCF4-YFP^{N}/LPA1-YFP^{C}

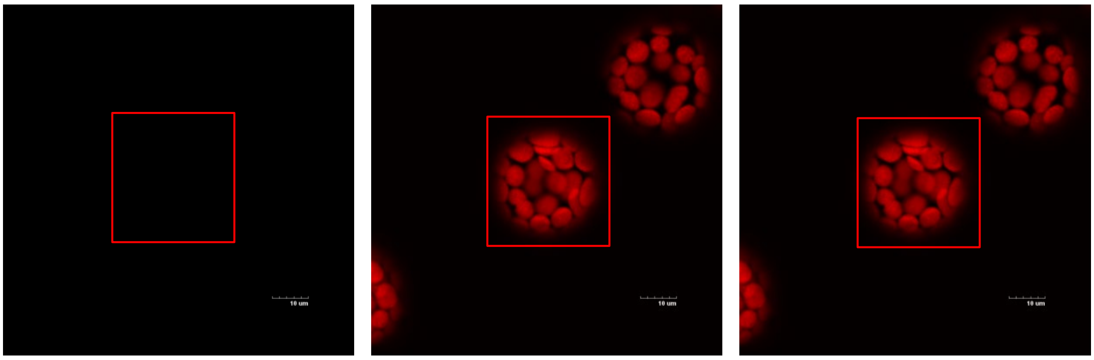

YCF4-YFP^{N}/LPA2-YFP^{C}

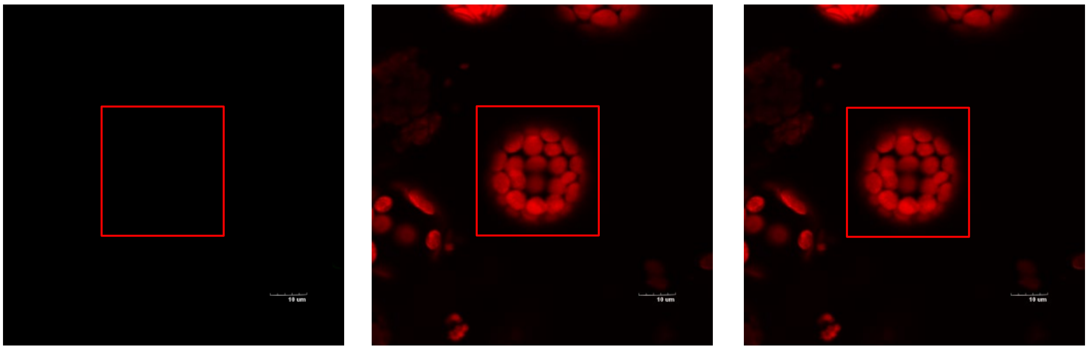

YCF4-YFP^{N}/YFP^{C}-SecY1

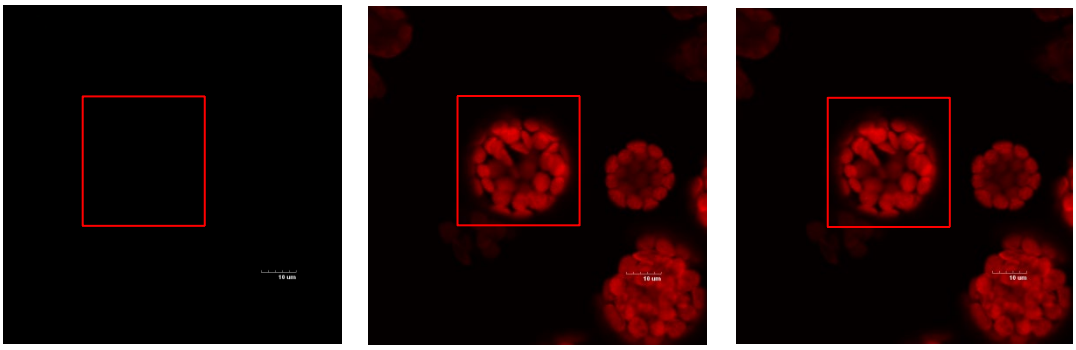

YCF4-YFP^{N}/YFP^{C}-SecE1

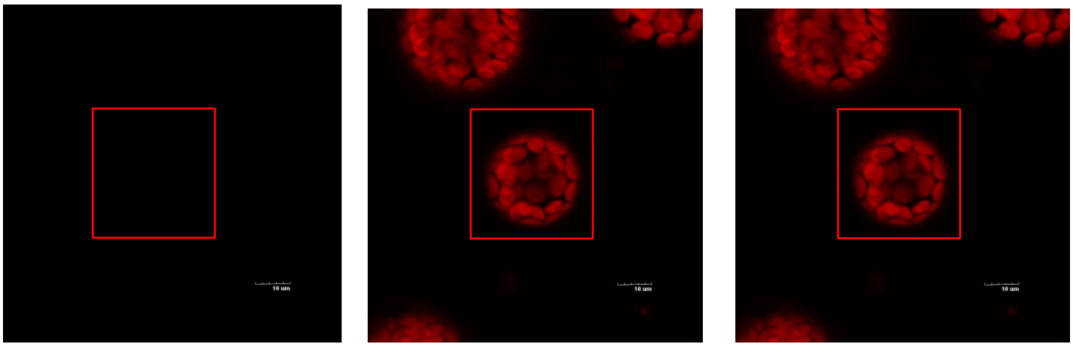

YCF4-YFP^{N}/FtsY-YFP^{C}

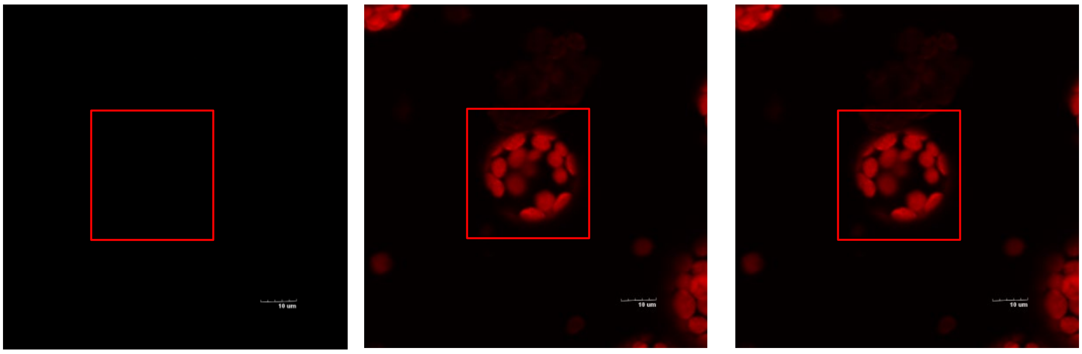

YCF4-YFP^{N}/cpSRP54-YFP^{C}

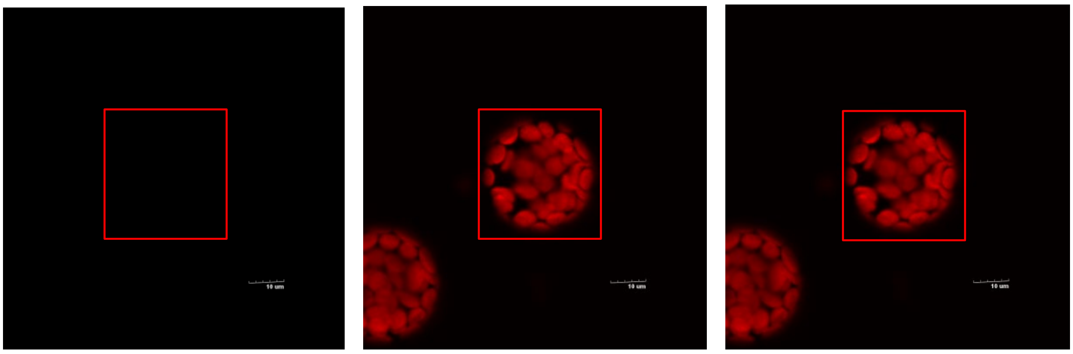

YCF4-YFP^{N}/Alb3-YFP^{C}

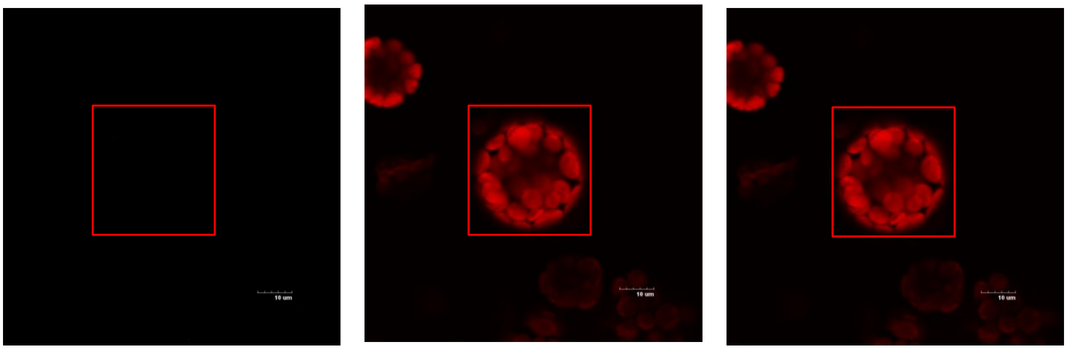

YCF4-YFP^{N}/SecA1-YFP^{C}

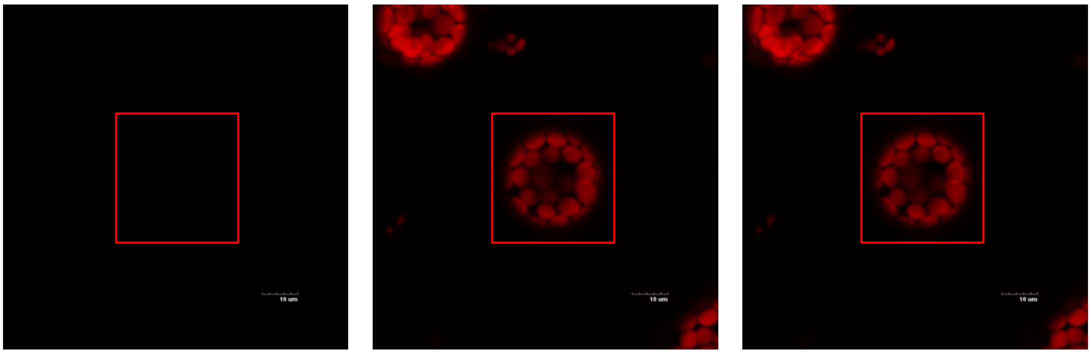

YCF4-YFP^{N}/PYG7-YFP^{C}

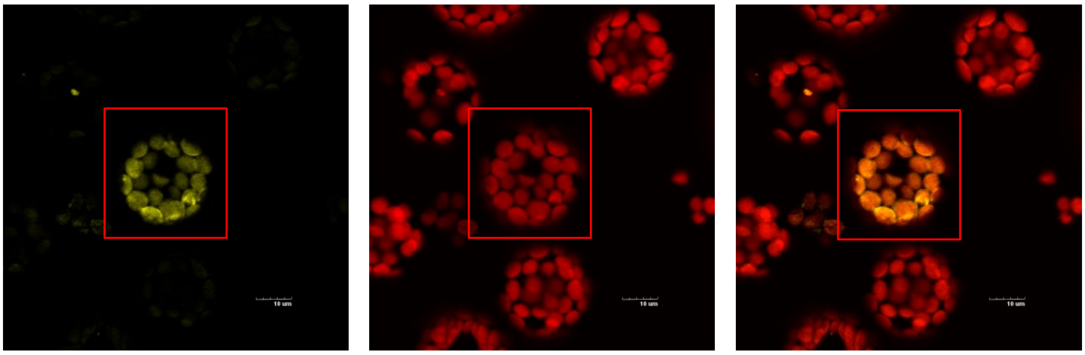

Figure S6c

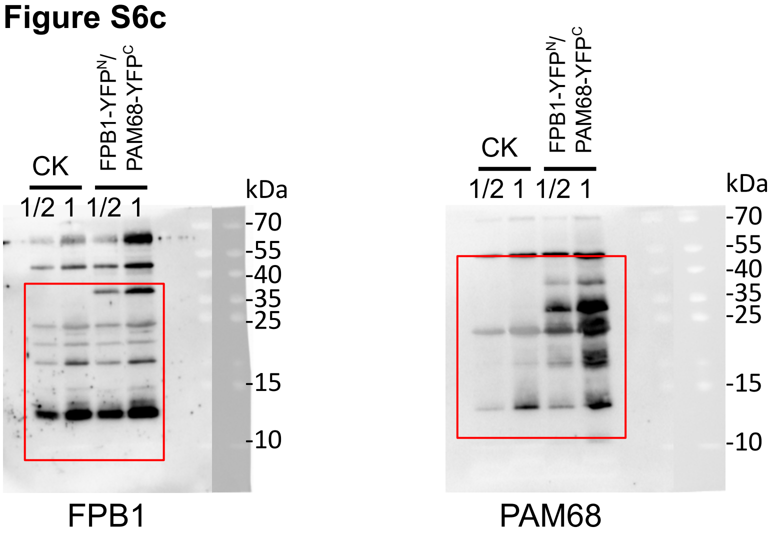

Figure S6d

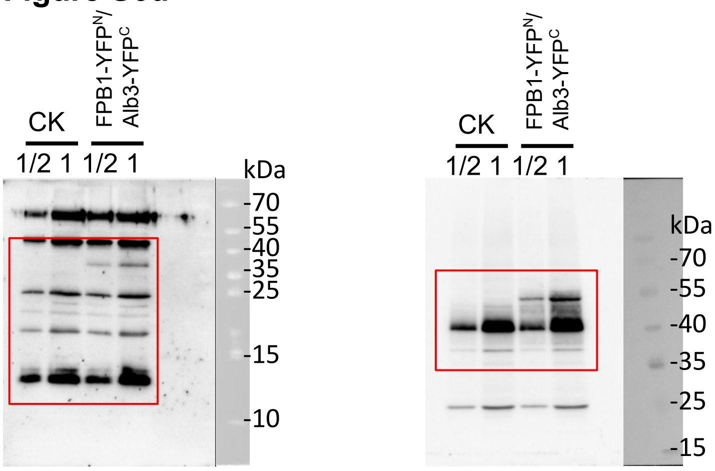

FPB1 Alb3

Figure S11

Figure S11a

AtpsbB (*ATCG00680*)

ATGGGTTTGCCTTGGTATCGTGTTCATACTGTTGTATTGAATGATCCCGGTCGTTTACTTGCTGTTCATATAATGCATACT
GCTCTAGTTGCTGGTTGGGCTGGTTCGATGGCTCTATATGAATTAGCAGTTTTTGATCCCTCCGACCCCGTTCTTGATC
CAATGTGGAGACAAGGTATGTTCGTTATACCTTTCATGACTCGTTTAGGAATAACCAATTCTTGGGGCGGTTGGAATATT
ACAGGGGGGACTATAACGAATCCGGGTCTTTGGAGTTACGAAGGGGTAGCCGGAGCACATATAGTGTTTTCTGGTTTG
TGCTTCTTGGCAGCTATTTGGCATTGGGTATATTGGGATCTAGAAATTTTTTGTGATGAACGTACAGGAAAACCTTCTTT
GGATTTGCCCAAGATTTTTGGAATTCATTTATTTCTTTCAGGAGTGGCTTGCTTCGGTTTTGGCGCATTTCATGTAACAG
GATTATATGGTCCTGGAATATGGGTATCCGACCCTTATGGACTAACCGGAAAGGTACAACCCGTAAACCCGGCGTGGG
GCGTGGAGGGTTTTGACCCTTTTGTTCCGGGAGGAATAGCCTCTCATCATATTGCAGCAGGGACGTTGGGTATATTAG
CGGGCCTATTCCATCTTAGTGTTCGTCCGCCTCAACGTCTATACAAAGGATTACGTATGGGCAATATTGAAACCGTCCT
TTCCAGTAGTATTGCTGCTGTCTTTTTTGCAGCTTTTGTTGTTGCTGGAACTATGTGGTATGGTTCTGCAACTACTCCCA
TCGAATTATTTGGTCCTACTCGTTATCAATGGGATCAGGGATACTTTCAACAAGAAATATATCGAAGAGTTAGTGCTGGA
CTGGCTGAAAATCAAAGTTTATCAGAAGCTTGGGCGAAAATTCCTGAAAAATTAGCTTTTTATGATTATATTGGTAATAAT
CCAGCAAAAGGGGGGTTATTCCGAGCGGGTTCAATGGACAATGGGGATGGAATAGCTGTTGGATGGTTAGGCCACCC
CGTCTTTAGAAATAAAGAAGGGCGTGAACTTTTTGTACGCCGCATGCCTACTTTTTTTGAAACTTTTCCGGTAGTTTTG
GTAGACGGAGACGGAATTGTTAGAGCTGACGTCCCGTTTAGAAGGGCAGAATCAAAATATAGTGTCGAACAAGTAGGT
GTAACTGTTGAGTTTTATGGTGGTGAACTCAATGGAGTGAGTTATAGTGATCCCGCAACTGTGAAAAAATATGCTAGAC
GGGCTCAATTGGGTGAGATTTTTGAATTAGATCGTGCGACTTTGAAATCTGATGGTGTTTTTCGTAGCAGCCCAAGAG
GTTGGTTTACGTTTGGACATGCTTCGTTTGCTCTACTTTTCTTCTTTGGACACATTTGGCATGGTGCTAGAACCCTCTT
CAGAGATGTTTTTGCTGGTATTGATCCAGATTTGGATGCTCAAGTGGAATTCGGGGCATTCCAAAAACTTGGAGATCCA
ACTACAAAACGACAAGCAGTCTGA

ZmpsbB (*NC_001666.2*)

ATGGGTTTGCCTTGGTATCGTGTTCATACTGTCGTATTGAATGATCCGGGTCGATTGCTTTCGGTGCATATAATGCACAC
AGCTCTAGTTTCTGGTTGGGCTGGTTCGATGGCTTTATACGAATTAGCGGTTTTTGATCCCTCTGATCCTGTTCTGGAT
CCAATGTGGAGACAAGGTATGTTCGTCATCCCCTTCATGACTCGTTTAGGAATAACGAATTCGTGGGGTGGTTGGAGT
ATTTCAGGAGGAACTGTAACGAATCCGGGTATTTGGAGTTATGAAGGTGTGGCAGGTGCGCATATTGTGTTTTCTGGC
TTGTGTTTCTTGGCAGCTATCTGGCATTGGGTATATTGGGACCTAGAAATATTCTGTGATGAGCGGACGGGAAAACCTT
CTTTGGATTTGCCCAAGATCTTTGGAATTCATTTATTTCTTGCAGGGGTGGCTTGTTTTGGCTTTGGTGCATTTCATGTA
ACGGGTTTATATGGCCCTGGGATATGGGTGTCCGATCCTTACGGACTCACTGGAAAAGTACAAGCTGTAAATCCTGCG
TGGGGTGCAGAAGGTTTTGATCCTTTCGTTCCGGGAGGAATAGCTTCGCATCATATTGCTGCGGGTACTTTGGGCATA
TTAGCGGGCCTATTCCATCTAAGTGTCCGTCCGCCTCAACGTCTATACAAAGGGTTACGTATGGGCAATATTGAAACTG
TACTTTCCAGTAGTATCGCTGCTGTTTTTTTTGCTGCTTTCGTAGTTGCCGGAACTATGTGGTATGGATCAGCAACTACC
CCAATCGAATTATTTGGGCCTACTCGTTATCAGTGGGATCAGGGATACTTTCAGCAAGAAATATATCGAAGAGTTAGCGA

TGGGTTAGCCGAAAATCTTAGTTTATCAGAAGCTTGGTCTAAAATTCCCGAAAAATTAGCCTTTTATGATTATATTGGTAA
TAATCCGGCAAAGGGGGGATTATTCAGAGCAGGCTCAATGGACAATGGGGATGGCATAGCTGTTGGATGGTTAGGACA
TCCCGTCTTTAGAGATAAAGAAGGACGCGAGCTTTTTGTACGTCGTATGCCTACTTTTTTTGAAACATTTCCGGTCGTTT
TGGTAGATGAAGAGGGAATTGTGAGAGCGGACGTTCCTTTTAGAAGAGCAGAATCCAAATATAGTGTTGAACAAGTAG
GCGTAACGGTGGAGTTCTATGGTGGCGAACTTAATGGAGTAAGTTATTCTGATCCTGCTACTGTAAAAAAATATGCGCG
GCGTGCTCAATTAGGGGAAATTTTTGAATTAGATCGAGCTACTTTGAAATCAGATGGTGTTTTTCGCAGCAGTCCAAGG
GGTTGGTTCACTTTTGGTCATGCTACCTTTGCTTTGCTCTTCTTTTTCGGACACATTTGGCATGGCGCTCGAACCTTGT
TCCGAGATGTTTTTGCTGGTATTGATCCAGACTTGGATGCTCAAGTGGAATTTGGAACATTCCAAAAAGTTGGAGATCC
AACTACAAGGAGACAGGCAGCCTGA

Figure S11b

AtCP47 (ATCG00680)

MGLPWYRVHTVVLNDPGRLLAVHIMHTALVAGWAGSMALYELAVFDPSDPVLDPMWRQGMFVIPFMTRLGITNSWGGWN
ITGGTITNPGLWSYEGVAGAHIVFSGLCFLAAIWHWVYWDLEIFCDERTGKPSLDLPKIFGIHLFLSGVACFGFGAFHVTGLY
GPGIWVSDPYGLTGKVQPVNPAWGVEGFDPFVPGGIASHHIAAGTLGILAGLFHLSVRPPQRLYKGLRMGNIETVLSSSIAA
VFFAAFVVAGTMWYGSATTPIELFGPTRYQWDQGYFQQEIYRRVSAGLAENQSLSEAWAKIPEKLAFYDYIGNNPAKGGLF
RAGSMDNGDGIAVGWLGHPVFRNKEGRELFVRRMPTFFETFPVVLVDGDGIVRADVPFRRAESKYSVEQVGVTVEFYGG
ELNGVSYSDPATVKKYARRAQLGEIFELDRATLKSDGVFRSSPRGWFTFGHASFALLFFFGHIWHGARTLFRDVFAGIDPD
LDAQVEFGAFQKLGDPTTKRQAV

ZmCP47 (NP_043049.1)

MGLPWYRVHTVVLNDPGRLLSVHIMHTALVSGWAGSMALYELAVFDPSDPVLDPMWRQGMFVIPFMTRLGITNSWGGW
SISGGTVTNPGIWSYEGVAGAHIVFSGLCFLAAIWHWVYWDLEIFCDERTGKPSLDLPKIFGIHLFLAGVACFGFGAFHVTG
LYGPGIWVSDPYGLTGKVQAVNPAWGAEGFDPFVPGGIASHHIAAGTLGILAGLFHLSVRPPQRLYKGLRMGNIETVLSSSI
AAVFFAAFVVAGTMWYGSATTPIELFGPTRYQWDQGYFQQEIYRRVSDGLAENLSLSEAWSKIPEKLAFYDYIGNNPAKGG
LFRAGSMDNGDGIAVGWLGHPVFRDKEGRELFVRRMPTFFETFPVVLVDEEGIVRADVPFRRAESKYSVEQVGVTVEFY
GGELNGVSYSDPATVKKYARRAQLGEIFELDRATLKSDGVFRSSPRGWFTFGHATFALLFFFGHIWHGARTLFRDVFAGID
PDLDAQVEFGTFQKVGDPTTRRQAA
